# Impact of Online Interactive Decision Tools on Women’s Decision-Making Regarding Breast Cancer Screening: Systematic Review and Meta-Analysis

**DOI:** 10.2196/65974

**Published:** 2025-01-29

**Authors:** Patricia Villain, Laura Downham, Alice Le Bonniec, Charlotte Bauquier, Olena Mandrik, Tom Nadarzynski, Lorie Donelle, Raúl Murillo, Eleni L Tolma, Sonali Johnson, Patricia Soler-Michel, Robert Smith

**Affiliations:** 1 International Agency for Research on Cancer World Health Organization Lyon France; 2 Pôle de Psychologie Sociale, Inserm U1296 Université Lumière Lyon 2 Lyon France; 3 Sheffield Centre for Health and Related Research The University of Sheffield Sheffield United Kingdom; 4 School of Social Sciences University of Westminster London United Kingdom; 5 College of Nursing University of South Carolina Columbia, SC United States; 6 Centro Javeriano de Oncología Hospital Universitario San Ignacio Bogota Colombia; 7 Department of Primary Care and Population Health University of Nicosia Medical School Nicosia Cyprus; 8 Union for International Cancer Control Geneva Switzerland; 9 Centre Régional de Coordination des Dépistages des Cancers Auvergne-Rhône-Alpes Site Rhône & Métropole de Lyon Lyon France; 10 Cancer Screening, American Cancer Society Atlanta, GA United States

**Keywords:** breast cancer screening, decision-making, online interactive, decision aid, average risk, shared decision-making, screening participation, cognitive determinants, women

## Abstract

**Background:**

The online nature of decision aids (DAs) and related e-tools supporting women’s decision-making regarding breast cancer screening (BCS) through mammography may facilitate broader access, making them a valuable addition to BCS programs.

**Objective:**

This systematic review and meta-analysis aims to evaluate the scientific evidence on the impacts of these e-tools and to provide a comprehensive assessment of the factors associated with their increased utility and efficacy.

**Methods:**

We followed the 2020 PRISMA (Preferred Reporting Items for Systematic Reviews and Meta-Analyses) guidelines and conducted a search of MEDLINE, PsycINFO, Embase, CINAHL, and Web of Science databases from August 2010 to April 2023. We included studies reporting on populations at average risk of breast cancer, which utilized DAs or related e-tools, and assessed women’s participation in BCS by mammography or other key cognitive determinants of decision-making as primary or secondary outcomes. We conducted meta-analyses on the identified randomized controlled trials, which were assessed using the revised Cochrane Risk of Bias 2 (RoB 2) tool. We further explored intermediate and high heterogeneity between studies to enhance the validity of our results.

**Results:**

In total, 22 different e-tools were identified across 31 papers. The degree of tailoring in the e-tools, specifically whether the tool was fully tailored or featured with tailoring, was the most influential factor in women’s decision-making regarding BCS. Compared with control groups, tailored e-tools significantly increased women’s long-term participation in BCS (risk ratio 1.14, 95% CI 1.07-1.23, *P*<.001, *I*^2^=0%). Tailored-to-breast-cancer-risk e-tools increased women’s level of worry (mean difference 0.31, 95% CI 0.13-0.48, *P*<.001, *I*^2^=0%). E-tools also improved women’s adequate knowledge of BCS, with features-with-tailoring e-tools designed and tested with the general population being more effective than tailored e-tools designed for or tested with non-BCS participants (χ^2^_1_=5.1, *P*=.02). Features-with-tailoring e-tools increased both the rate of women who intended not to undergo BCS (risk ratio 1.88, 95% CI 1.43-2.48, *P*<.001, *I*^2^=0%) and the rate of women who had made an informed choice regarding their intention to undergo BCS (risk ratio 1.60, 95% CI 1.09-2.33, *P*=.02, *I*^2^=91%). Additionally, these tools decreased the proportion of women with decision conflict (risk ratio 0.77, 95% CI 0.65-0.91, *P*=.002, *I*^2^=0%). Shared decision-making was not formally evaluated. This review is limited by small sample sizes, including only a few studies in the meta-analysis, some with a high risk of bias, and high heterogeneity between the studies and e-tools.

**Conclusions:**

Features-with-tailoring e-tools could potentially negatively impact BCS programs by fostering negative intentions and attitudes toward BCS participation. Conversely, tailored e-tools may increase women’s participation in BCS but, when tailored to risk, they may elevate their levels of worry. To maximize the effectiveness of e-tools while minimizing potential negative impacts, we advocate for an “on-demand” layered approach to their design.

## Introduction

Breast cancer constitutes 11.6% (2.309 million new cases) of global cancer incidence and 6.9% (665,684 deaths) of global cancer mortality [1].

Mammography is the most widely used screening method in breast cancer screening (BCS) programs for detecting breast cancer. These programs primarily target women at average risk of the disease. A woman is considered at average risk if she lacks risk factors associated with a significantly increased likelihood of breast cancer, such as a personal history of the disease, a strong family history, a genetic mutation known to elevate risk (eg, a *BRCA* mutation), or prior high-dose radiation therapy to the chest at a young age [2-4]. Conversely, a woman is considered at high or very high risk of breast cancer if she has 1 or more of these risk factors. Such high-risk women do not participate in standard BCS programs; instead, they undergo more personalized screening options [2,5].

Despite the proven efficacy of mammography-based BCS and national health policies promoting regular screening for women at average risk, participation in BCS remains suboptimal in many countries, falling well below the recommended rates set by the European Union and other organizations [6-8].

Numerous barriers to women undergoing BCS have been identified in the literature, such as fears of a breast cancer diagnosis or the mammography procedure itself [9-11]. For some women, participation in a BCS program may also be perceived as a dilemma due to ongoing debates surrounding BCS. Indeed, scientific and medical discussions continue about specific aspects of BCS, including the optimal screening interval [12,13]. Furthermore, public discourse often highlights controversies and potential risks associated with BCS, such as overdiagnosis and overtreatment [14,15].

The use of decision aids (DAs), either independently or as part of a shared decision-making (SDM) process, appears particularly suitable and important for both women and BCS programs. These tools help women make an “informed choice” about their participation in BCS, enabling them to make a reasoned and justifiable decision after receiving reliable and sufficient information about the procedure or examination and its associated risks [16]. DAs are evidence-based cognitive tools designed to elucidate the decision-making process and help patients clarify their values and preferences [17-19]. Similarly, SDM is described as an interactive, balanced, step-by-step discussion between health professionals (HPs) and patients, often incorporating DAs to support the dialogue [20,21].

Patients faced with decisions about cancer screening (including BCS) who are exposed to DAs tend to be better informed and more aligned with their values [19,22-26]. Although promising, evidence regarding the effectiveness of SDM remains limited, particularly in real-world settings where its implementation is often hindered by the busy and demanding schedules of HPs [27-30]. Additionally, there is insufficient evidence on when it is more beneficial for patients to engage in an SDM discussion with an HP rather than relying solely on a DA [20,31-34].

Global interest in digital health and eHealth tools, including telehealth, has surged in recent years, largely driven by the COVID-19 pandemic [35-37]. Online delivery offers the potential to enhance access to DAs for patients and HPs, particularly for those in underserved communities, while also enabling more integrated implementation of SDM. As a result, incorporating online DAs and related tools into BCS programs could support women in making informed decisions. A review of web-based DAs found these tools to be effective in increasing women’s knowledge and ability to make informed choices while reducing decisional conflict or dilemmas about BCS [38].

However, to better inform future BCS programs about the utility and implementation of online tools for women’s decision-making, it is necessary to go beyond Yu et al’s review [38] and provide additional evidence. First, it is important to evaluate not just web-based DAs but also all types of online interactive tools (e-tools) to fully explore their potential for supporting women’s decision-making about BCS [38-40]. An overall assessment of the effect of these e-tools, including web-based DAs, on women’s participation in BCS and important cognitive determinants in decision-making (eg, women’s attitudes or perceptions of risk) was lacking [19,22,25,26,38]. Additionally, there was a need for a comprehensive evaluation of factors associated with the greater utility and effectiveness of these e-tools to support more informed BCS programs. This includes exploring aspects related to information delivery (eg, mobile vs computer-based e-tools, degree of message tailoring), user characteristics (eg, nonadherent to BCS vs the general population), and the methods used to evaluate these e-tools (ie, the outcomes assessed to measure their impact on decision-making). Finally, evidence of the effect of these e-tools on the SDM process was also missing.

Through a combined systematic review and meta-analysis, our research aimed to address these evidence gaps and synthesize the scientific literature on e-tools designed to support decision-making in the context of BCS by mammography. The primary objective of this review was to inform future BCS programs about the utility and implementation of such e-tools.

## Methods

### Review Design

This review was registered with the International Prospective Register of Systematic Reviews (PROSPERO; CRD42020164479) [41]. The main deviation from the original protocol was the inclusion of studies without comparator arms. This change was made for 2 reasons: (1) a preliminary review of the databases indicated that the total number of studies might be limited if only those with comparators were included, and (2) to provide the most comprehensive assessment of the effects of e-tools, including web-based DAs, on women’s participation in BCS and important cognitive determinants in decision-making. The type of design for each study included in this review was considered when assessing the overall certainty of the evidence using the GRADE (Grading of Recommendations Assessment, Development, and Evaluation) methodology (outlined below). A workshop held in December 2019, organized by PV, on developing and implementing e-tools as DAs to support women’s decision-making about BCS, played a significant role in shaping the development of this review. We conducted our systematic review in accordance with the 2020 PRISMA (Preferred Reporting Items for Systematic Reviews and Meta-Analyses) guidelines [42] (see Table S1 in Multimedia Appendix 1).

### Study Search, Selection, and Data Extraction

We systematically searched the MEDLINE (via PubMed), PsycINFO, Embase, CINAHL, and Web of Science databases from August 2010 to August 2020, with an update in April 2023 (see Appendix S1 in Multimedia Appendix 1). We limited the search to articles published from 2010 onward to minimize the risk of including e-tools that may have become technologically obsolete—those using outdated technology and no longer considered useful, efficient, or functioning well compared with newer alternatives [43]. Additional websites (eg, the International Patient Decision Aid Standards [IPDAS] website) were also searched nonsystematically. The following inclusion criteria were applied: (1) women considered to be at average risk of breast cancer, that is, those without risk factors associated with a significantly increased risk of the disease [2-4]; (2) the tool, whether identified as a DA or not, was used for women’s decision-making about BCS, aimed to inform users at least about BCS by mammography, and was an e-tool defined as one that runs on the internet, computer, phone/tablet, or other electronic device and is interactive (ie, allowing users to “participate in modifying the form and content of a mediated environment in real time”) [39,40]; and (3) the study, of any design, reported as primary or secondary outcomes any of the following: women’s participation in BCS by mammography (behavior), intention to participate, knowledge, attitudes, self-efficacy, worry, perceptions of risk, regrets, decisional conflict, and informed choice—measures shown to reflect decision-making and behavior and to be important in decision-making [19,22,25,26]. Exclusion criteria were as follows: (1) participants at high risk of breast cancer, that is, those with 1 or more risk factors associated with a significantly increased risk of the disease [2-4]; (2) screening limited to breast examination by an HP or self-examination; (3) non–peer-reviewed publications; and (4) studies focused on BCS cessation.

The search yielded 16,061 records, which were managed using Covidence software [44]. Three independent reviewers (PV, ALB, and CB) assessed titles and abstracts, followed by full-text evaluation against the study inclusion criteria. A total of 227 papers were selected for full-text eligibility assessment, and 196 were subsequently excluded. A data extraction form was used (see Appendix S2 in Multimedia Appendix 1); it was completed by a main reviewer (PV or ALB) and independently assessed by 2 additional reviewers (PV, LD, ALB, or CB).

### Qualitative and Quantitative Syntheses

Extracted data were synthesized both qualitatively and quantitatively, as detailed later.

The qualitative synthesis aimed to provide an overview of the characteristics of the e-tools and a description of the reported outcomes and the instruments used to measure them. These are reported in the Results section. Additionally, the characteristics of the studies included in this review and the study populations are reported in Tables S2 and S3, respectively, in Multimedia Appendix 1.

Quantitative synthesis was conducted to report the effect of the e-tools on women’s decision-making about BCS. Main results are reported in the Results section, with additional results reported in Appendix S4 and Figures S1-S8 in Multimedia Appendix 1.

### Description of the e-Tools

The main descriptive variables were identified through discussions and consensus among the authors. These variables were considered important as they could potentially influence the effects of e-tools on women’s decision-making about BCS. The e-tools identified in this review were classified as either “tailored”—when the e-tools provided individualized or personalized notifications, messages, or information based on an individual’s assessment [40,45,46]—or “features with tailoring”—when the e-tools presented some degree of tailored information but not based on an individual assessment [40].

### Effect of the e-Tools on Women’s Decision-Making About BCS

#### Overview

The effect of the e-tools was assessed through the following outcomes: women’s participation in BCS by mammography (behavior), intention to participate, knowledge, attitudes, self-efficacy, worry, perceptions of risk, regrets, decisional conflict, and informed choice. These outcomes have been shown to reflect decision-making and behavior and are particularly important in the context of BCS [19,22-26,38]. The effects of the e-tools on SDM or communication with HPs were also evaluated.

For each outcome, quantitative synthesis was conducted and reported through a 2-step process. First, where possible, we performed meta-analyses with the randomized controlled trials (RCTs) identified in this review, following Cochrane guidance [47]. Additionally, the synthesis without meta-analysis (SWiM) guideline was used to synthesize evidence from RCTs not included in the meta-analyses or other study designs (see Table S2 in Multimedia Appendix 1) [48]. Second and finally, for each outcome, the overall certainty of the evidence collected through the meta-analyses and SWiM exercise was independently evaluated by PV and LD using the GRADE methodology [49-51]. Results from the risk-of-bias assessment (discussed later) were used to downgrade the overall certainty of the evidence, if necessary. Because of the low total number of studies included in this review, we did not downgrade the overall certainty of the evidence, assessed with GRADE, based on the imprecision (number of studies, SD) criteria, except when only 2 studies (meta-analyses) or 1 study were available. This applied to the “intention not to participate,” “worry,” “perception of risk,” and “decisional conflict” outcomes assessed through meta-analyses, as well as the “self-efficacy,” “regret,” and “discussion/SDM” outcomes. Any discrepancies between the 2 independent reviewers were resolved through the intervention of a third reviewer (RS).

#### Meta-Analyses and Risk of Bias

Meta-analyses were conducted with RCTs following Cochrane guidance [47] (see also Part A in Appendix S3 in Multimedia Appendix 1).

Data were extracted from the original publications, and additional information was obtained by directly contacting the authors when necessary. Only data related to BCS by mammography, as reported by the authors for “women,” were extracted.

PV and LD, along with ALB or CB, independently performed the risk-of-bias assessment for all RCTs included in this review using the revised Cochrane Risk-of-Bias 2 (RoB 2) tool for RCTs [52]. Any discrepancies between the 2 independent reviewers were resolved through the intervention of a third reviewer. As a result of the low number of studies, none were excluded based on RoB 2 results. The level of risk for each RoB 2 domain (A: randomization process; B: deviations from the intended protocol; C: missing data; D: outcome measurement; E: reporting results) and overall risk (F) were evaluated for each RCT and reported on the forest plots (discussed below) as low (green), moderate (yellow), or high (red).

To minimize sources of heterogeneity between studies, we chose to include only RCTs that reported the most similar outcomes based on their definitions and the instruments used to measure them in the meta-analyses described in this review. Therefore, contrary to Yu et al’s review [38], we reported the decisional conflict outcome only for RCTs using the “SURE” scale and did not report the pooled effect for the “regret” outcome.

We used Review Manager software (RevMan, version 5.4.1; Cochrane) [53] to conduct meta-analyses, perform subgroup analyses, and display forest plots, including risk-of-bias assessments.

We reported pooled estimates as mean difference (MD) for continuous variables (adequate knowledge, worry, and accurate perception of risk) and relative risk/risk ratio (RR) for dichotomous variables (other outcomes). Heterogeneity or inconsistency between studies was assessed using the *I*^2^ statistic. A fixed-effect (FE) model was applied when low heterogeneity was found (*I*^2^≤30), while a random-effects (RE) model was used in other cases. Results from meta-analyses with intermediate or high heterogeneity between studies (*I*^2^≥35) were further explored to strengthen the validity of our findings.

#### Exploration of Heterogeneity and Subgrouping Analysis

Where meta-analyses showed intermediate or high heterogeneity between studies (*I*^2^≥35), further analyses were conducted. The main results are reported in the text (see also Figures S1-S8 and Appendix S4 in Multimedia Appendix 1). Two approaches were used to explore the causes of intermediate/high heterogeneity [47]: (1) exclusion of 1 study when it differed from the others in the meta-analysis based on either the type of e-tool used (eg, web application or mobile app) or the control used (eg, a website or a video). We also explored the exclusion of studies with a high risk of bias(es). Exclusion of Lee et al’s [54] study, which used a mobile app downloaded on a mobile phone, or Roberto et al’s study [55], which used a website as a control, in the meta-analysis assessing short-term participation or adequate knowledge, respectively, was effective in reducing heterogeneity (reported); (2) additionally, subgroup analyses were conducted based on various variables that could have explained the source of heterogeneity, for example, characteristics of the e-tools such as the type or degree of tailoring, the type of outcomes or instruments, or characteristics of the study populations such as age, ethnicity/rurality, and nonparticipation in previous BCS by mammography (see also Table S3 in Multimedia Appendix 1).

Besides exploring heterogeneity, subgroup analysis was also conducted to examine the effect of different variables (described above) on the reported outcomes.

## Results

### Overview of Included Studies and e-Tools

**Figure 1:**
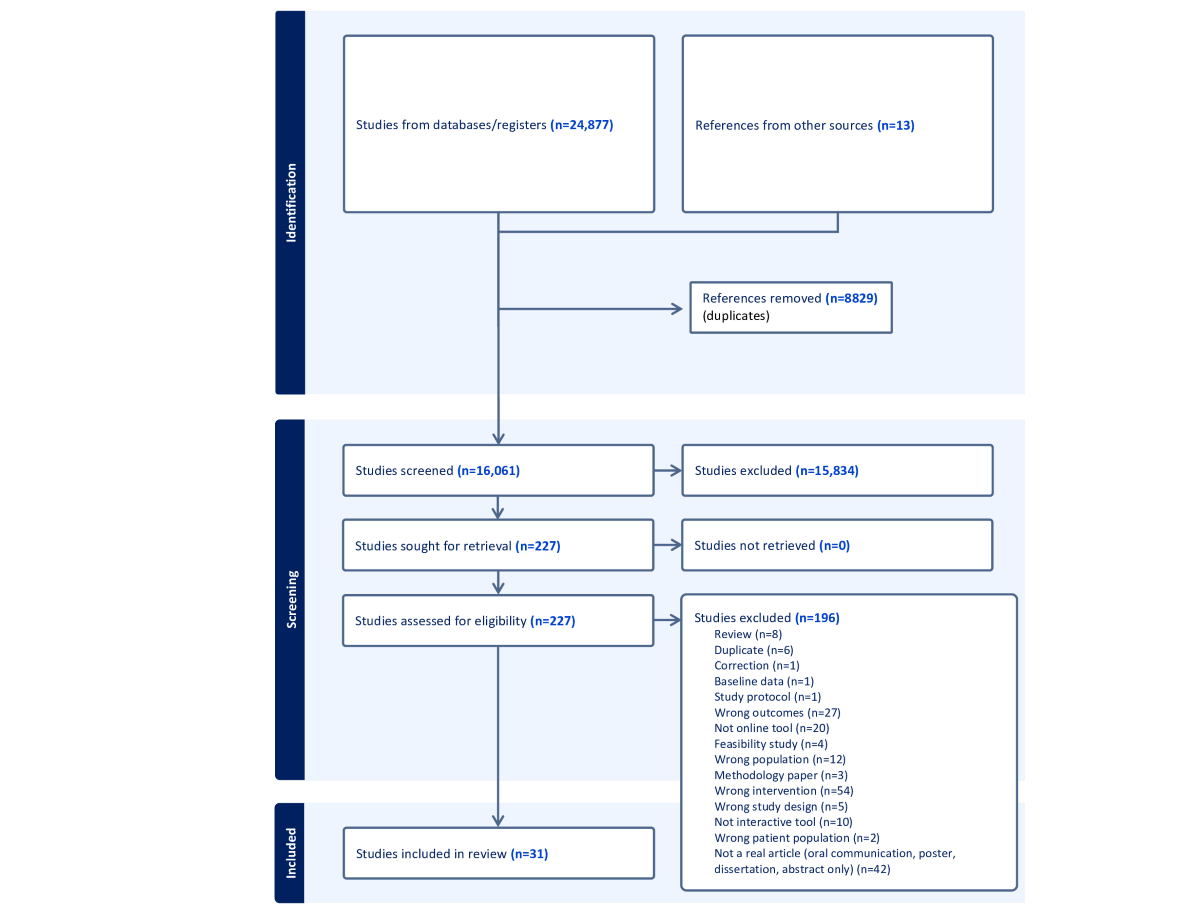
PRISMA (Preferred Reporting Items for Systematic Reviews and Meta-Analyses) 2020 flow diagram. The diagram was generated using the Covidence software [44].

We identified 31 published papers/studies (Figure 1; also see Table S2 in Multimedia Appendix 1) [54-84], representing 22 different e-tools [54-75]. The majority of these were designed and evaluated in the United States (n=16) and tested through RCTs (n=14). Study populations are detailed in Table S3 in Multimedia Appendix 1.

### Description of the e-Tools

Among the 22 e-tools, the majority (n=16) were web applications, either standalone or integrated into patient portals [55,56,58,59,61,63,65,67-75] (Table 1; see also Table S4 in Multimedia Appendix 1).

Two e-tools used artificial intelligence (AI)–based virtual doctors [58,64].

Most of the e-tools (16/22; Table 1) were tailored to the individual’s (1) breast cancer risk (n=7) [63,65,71-75]; (2) barriers, beliefs, or screening status identified at baseline (n=8) [56,57,59,61,64,66-68]; or (3) preferences regarding the content, number, and timing of daily messages [54]. Of these 16 tailored e-tools, 8 targeted nonparticipants in BCS (see Table S3 in Multimedia Appendix 1) [54,56,57,59,63,66-68]; 11 out of the 16 tailored e-tools included specific features designed to facilitate or enhance opportunities and the quality of discussions about BCS between women and HPs (Table 1) [54,59,61,63-65,67,72-75].

The remaining 6 e-tools (out of 22) were not tailored to individual users and were classified as “features-with-tailoring” e-tools (Table 1) [55,58,60,62,69,70]. None of these tools were evaluated specifically with BCS nonattenders (see Table S3 in Multimedia Appendix 1). The tailoring features in these tools were designed to either (1) address BCS screening barriers for specific subpopulations (n=2) [60,62] or adapt the communication style of an AI-based virtual HP to meet the needs of specific user groups [58], or (2) prompt users to identify their personal beliefs and values regarding BCS (Table 1) [55,69,70].

**Table 1:** Description of the e-tools^a^ used to help women’s decision-making about breast cancer screening by mammography.

Descriptive variables	Characteristics of e-tool
Theoretical model^b^	Not reported [55,58,59,61,62,67,69,70,74,75] The Transtheoretical Model of Behavior Change [56,57,64,66,68], the Health Belief Model [54,56,57,66], the Decisional Conflict Theory [72,73], the Exemplification Theory [63,71], the Elaboration Likelihood Model of Persuasion [71], the Self-Regulation Model [65], the Theory of Planned Behavior [56], the Conceptual Framework to Measure Engagement [60], and the Fogg Behavioral Model [54]
Decision aids^c^	Identified as decision aids [55,61,63,67,69-75] All except 2 e-tools [61,71] were reported to be developed based on international standards: either the International Patient Decision Aid Standards Framework [55,63,69,70,72-74] or the Ottawa Decision Support Framework [67,75] Quality assessment against International Patient Decision Aid Standards criteria was performed by the authors themselves [63,75] or by others and through a systematic review [85] in which 4 e-tools were evaluated and assessed to be of either high overall quality [69,70,75] or below the overall mean quality [72] Not identified as decision aids [54,56-60,62,64-66,68]
Type^d^	Standalone web-based applications [55,56,58,63,65,68-71,74,75], which were optimized for smartphones [72]; Fissler et al’s e-tool [58] used an artificial intelligence–based virtual doctor Patient portal: either a new one [61] or “embedded” or “linked” to an existing one [59,67,73] A mobile app either on a smartphone with a global positioning system [54] or on an iPad [64]; Walsh et al’s e-tool [64] used an artificial intelligence–based virtual doctor Interactive DVD [57,66] Social media–based: either Facebook [60] or WhatsApp [62]
Information provided	Breast cancer screening and breast cancer [54,55,57,58,60,62,63,68-75] Breast cancer screening and breast cancer with additional information^e^, such as other cancer screening [56,59,61,64,66,67], or other prevention messages (eg, diet) [59,61,64,65]
Degree of tailoring^f^	Tailored To address woman’ breast cancer risk^g^ [63,65,71-75]; some e-tools also allowing to clarify value^h^ [63,65,72-75] To address individuals’ barriers, beliefs, or screening status at baseline [56,57,59,61,64,66-68], including the stage of change based on the Transtheoretical Model [68] To adapt the content, number, and timing of daily messages to each individual [54] Features-with-tailoring To address the screening barriers specific to a group of the targeted population [60,62] To target the communication style of the factious health professional to the needs of a specific group of patients [58] To allow individuals to clarify their values^h^ [55,69,70]
Access to health professionals^i^	The e-tools were part of clinical pathways or referred to actual health care services [55,59,61,63,65,67,70,72,73,75] Being a patient portal or embedded in an existing one [59,61,67,73] patients with medical preappointments were automatically invited to use the tool [67] patient being directly informed of a care gap (eg, delay in getting breast cancer screening), and could directly book an appointment through the portal [59] both patients and health professionals were informed automatically when the former were at a higher risk of breast cancer [73] individual recommendations targeting 18 preventive health services were provided [61] Being available before/after a booked medical appointment: before [72,73], or most probably only or at the first instance at clinics [63,75], or in advance of a prescreening appointment made by breast cancer screening programs [55,70] Offering counseling sessions online or discussions with health professionals [65] Used artificial intelligence–based virtual doctor to simulate an appointment between a health professional and their patient [58,64], designed to encourage the user to continue [64] or to improve [58] discussion with a health professional Providing a personalized report All being downloadable [55,63,64,67,70,72-75] or probably being downloadable [61] Either a summary with a list of questions [61,63,64,67,72-75] or the results of the value clarification exerciseh [55,70] When the tools were part of clinical pathways, reports were put directly in the patient folder [63,64], a specific appointment to discuss was offered [72], or the report was sent to the health professional through the patient portal [61,67,73] Women were encouraged to either share or discuss this report with their health professional [61,63,67,72-75], or with people they trust [55], or to talk with a health professional about any concerns [64,70] or in case of indecision about screening [70] Being tailored e-tools based on risk: women at higher risk receive automatic messages to follow-up including talking with health professionals [65,72-75], and health professionals are informed automatically [73] Includes an option to contact a health navigator for assistance in navigating through cancer screening information, addressing technical problems, and providing transportation and interpretation services [54] With a global positioning system facilitating cancer screening clinics localization and other information [54]

^a^The e-tools were categorized into groups and subgroups based on the descriptive variables as indicated (see also Table S4 in Multimedia Appendix 1).

^b^Any psycho-theoretical model reported to be used to develop the e-tool.

^c^We classified the e-tools as “identified as decision aids” when the authors explicitly used “DA” or “decision-making” to describe the e-tool, or when the e-tool contained a decision aid/decision-making module or a link to decision aids. The remaining tools were classified as “not identified as decision aids.” A rapid assessment of these tools against the 5 previously described decision aids criteria [86], that is, (1) information about options, decisions, and outcomes, including benefits and harms; (2) evidence-based information on options; (3) probabilities; (4) value clarification; and (5) decision guidance, did not provide sufficient information to determine whether or not these tools could be classified as decision aids.

^d^The tools were classified as previously described [40,87].

^e^In this group of e-tools, the study population was either only women [56,65,66] or both men and women [59,61,64,67].

^f^Tools were classified as either “tailored” or “features-with-tailoring” e-tools, as previously defined [40,45,46]. Tailored tools provided individualized or personalized notifications based on an individual’s assessment. This assessment could be conducted through participants’ responses to questionnaires, which were either external to the e-tool and completed before its use or integrated into the e-tool itself, addressing factors such as the individual’s breast cancer risk or other characteristics. By contrast, “features-with-tailoring” e-tools offered some degree of personalization or tailoring, but it is not based on individual assessments.

^g^Risk was estimated using 2 validated instruments: the Breast Cancer Risk Assessment Tool for personal risk estimates and the Breast Cancer Genetics Referral Screening Tool for assessing familial breast cancer risk. In 1 study, a different instrument was used, and it was unclear whether the risk estimation was communicated to the women involved [74]. Among the 7 tailored-to-risk tools, 4 utilized risk assessment to exclude women with above-average breast cancer risk from accessing the decision aid, instead directing them to receive messages for appropriate follow-up based on best practices [72-75]. For the remaining 3 tools, risk assessment was conducted online to personalize the features of the tools according to each woman’s individual risk [63,65,71]. One tool provided 6 levels of tailored-to-risk messages, and its effects were evaluated [71].

^h^The tools aimed to encourage users to identify their personal beliefs and values regarding breast cancer screening. The methods used were all explicit, meaning they required active user participation—such as moving sliders, assigning weights to scales, or inputting numbers—to express their values, preferences, and concerns [88].

^i^Specific features aimed to facilitate access to or communication with health professionals.

### Effect of the e-Tools on Women’s Decision-Making About BCS

#### Overview of the Outcomes

The effect of the e-tools on women’s decision-making about BCS was assessed through various outcomes in the studies included in this review. A comprehensive overview of these outcomes and the instruments used to measure them are provided in Table 2.

The synthesized results regarding the effects of the e-tools are presented in the following paragraphs, with the outcomes listed in the same order as in Table 2. We encourage readers to refer to this table for the definitions of each outcome.

**Table 2:** Outcomes and instruments to assess the effect of e-tools on women’s decision-making about breast cancer screening by mammography.

Outcomes^a^		Definitions^b^ and instruments^c^
Participation (behavior) [54-57,59,61,63-67,70]		Authors reported the rates of women who have participated in breast cancer screening by mammography. Participation in breast cancer screening was reported by the authors as “participation rate” [55], “adherence” [57], “uptake” [54,63,70], “receipt” [54,56], “screening behavior” [65], “getting screened” [67], “being up to date” [64,66,67], or “screening gap closure” [59]. Instruments used to measure women’s participation were self-report questionnaires/surveys [54,55,65,70], health records [59,63,67], or both, with self-reports used to supplement missing medical records [56,57,61,64,66]. In 1 study [70], the authors differentiated participation in the breast cancer screening program, which was reported, and opportunistic screening. Depending on the study, participation was assessed as follows: At short-term (up to 6 months): measured at 0.5-2 months [55], at 3 months [59,67,70], at 4 months [61], or at 6 months [54,56,57]. In Krist et al [67], a non–randomized controlled trial, measures were done after each invitation phase. At long-term (12-16 months): measured at 12 months [63,65,66], at 14 months [64], or at 16 months [61]. It must be highlighted that in Champion et al’s [66] study, participation after the 12-month window was classified as “no screening.”
Intention to undergo breast cancer screening [54,55,60,68-74,76]		Intention, as defined in the Theory of Planned Behavior [89], is assumed to capture the motivational factors that influence a behavior; it indicates how hard people are willing to try or how much effort they are planning to exert to perform the behavior. Intention assessment was reported as the rates of women with intention/willingness to undergo breast cancer screening. Reder and Kolip [70] differentiated the breast cancer screening program and opportunistic screening and reported intention to participate in the breast cancer screening program. In the randomized controlled trials, intention was assessed just after using the e-tool [54,68-74] or 7-10 days after [55]. Generally, intention was assessed through 1 or 2 items in a questionnaire/survey with anchors defined by the authors themselves to be either cross-checked [54,55,68,70-74,76] or evaluated with a Likert scale [60,69]. Intention was formulated either as a general statement [55,73] or as a more specific one and anchored to a specific time or age. Specific time(s) was/were linked to the near future either now [69], in the next 3 months [70], in the coming/next year [60,68], or at different possible times (within 1 month, 3 months, or up to 1 year) [54]. In 1 study it was evaluated for a more distant future (in the next 1-2 years) [74]. When anchored to age, women’s intention was evaluated to start or continue in their forties [71,72], or to start at 50 years [71]. We used the cumulative value of intention to assess Lee et al’s [54] and Seitz et al’s [71] tools in the meta-analysis (see Part A in Appendix S3 in Multimedia Appendix 1): intention “to get screened within 3 months to 1 year” and intention “to start or continue in the forties, or to start at 50 years old” were reported.
Intention *not* to undergo breast cancer screening (negative intention) [69,70]		It was assessed in 2 randomized controlled trials and reported as the rates of women with intention/willingness not to undergo breast cancer screening [69,70].
Knowledge [54,55,62-65,67,69,70,72,77]		Knowledge was assessed about breast cancer screening (most studies) and, in some studies, about breast cancer. With Walsh et al’s [64] and Krist et al’s [67] e-tools, participants were asked to evaluate whether the tool improved their own knowledge; the authors reported rates of participants presenting different degrees of agreement [64,67,77]. With Eden et al’s [72] e-tool, women rated how much new information they learned from the e-tool on a scale from 1 (lowest) to 10 (highest), with the authors reporting median and IQR. In the remaining 7 e-tools (ie, 6 randomized controlled trials [54,55,63,65,69,70] and 1 mixed method study [62]), the instrument used to measure knowledge was more complex and heterogeneous; the instrument was a series of 5-18 items created or adapted by the authors to assess participants’ knowledge accompanied by anchors. The format of the anchors was either multichoice responses [62,63], both multichoice and numerical responses [55,69,70], true/false responses [54], or response scales of 1-4 [65]. Lee et al [54] used a validated knowledge instrument, which was revised to reflect the current American Cancer Society’s breast cancer screening guidelines. Adequate knowledge was reported as a quantitative value to reflect the number of items the participants answered correctly [54,55,62,63,65,69,70]. In some studies, the percentage of women with adequate knowledge was also reported; those were defined as women providing correct answers to at least half plus 1 of the total number of questions [55,69,70].
Attitudes about breast cancer screening [55,58,69,70]		Refers to the degree to which a woman has a favorable or unfavorable evaluation or appraisal of an undergoing breast cancer screening program [16,89]. In 4 randomized controlled trials [55,58,69,70], the instrument to measure attitudes toward undergoing breast cancer screening was adapted from Marteau et al’s [16] definition and measure. It was either 4 items [55,58,70] or 6 items from Dormandy et al’s scale [69,90]; all these items were linked to a Likert scale. In 1 of those randomized controlled trials [58], the authors reported attitudes score, with higher scores reflecting higher positive attitudes toward undergoing breast cancer screening. In the other 3 randomized controlled trials [55,69,70], Marteau et al’s [16] method was used. A predefined threshold was set to differentiate women with positive attitudes [55,70] or values [69], that is, women scoring higher than the threshold, from women with negative attitudes [55,70] or values [69], and the percentage of women with positive attitude was reported.
Self-efficacy [54,72]		Confidence in one’s ability to take action as described in the Health Belief Model [91]. Also includes the concept of perceived behavioral control in the Theory of Planned Behavior models, referring to the perceived ease or difficulty of performing the behavior and assuming to reflect past experience as well as anticipated impediments and obstacles [89]. Instruments used to measure self-efficacy were validated instruments. They were either the 8 related items from the Champion’s Health Belief Model Scale [54] or the Decision Self-Efficacy Scale [72]. The level of self-efficacy was reported. One component of the Decision Self-Efficacy scale measures self-confidence or belief in one’s ability to make decisions and participate in shared decision-making.
Worry [63,67,69,71,75,78]		Assessment of emotional reactions related to breast cancer was exclusively based on assessing changes in anxiety, worry, or fear either in general, “feeling fear and worry” [69,71,78], or specifically breast cancer [63,67,75]. In Krist et al’s [67] study, the level of fear and worry was assessed through a series of questions related to breast cancer prompted by the e-tool. The authors then evaluated whether discussion with health professionals was helpful to reduce women’s fears and worries if fears/worries were identified. For other e-tools, instruments used to measure worry were heterogeneous. It was a validated instrument: either the Lerman Breast Cancer Worry Scale [63] or adapted from the McCaul Breast Cancer Worry Scale [75]; a Likert scale was used to rate the answers, and the means of scoring was reported. In other cases, it was created by the authors themselves and was generally 1-2 questions linked to a Likert scale [69,71,78]. Seitz et al [71,78] adapted a previous instrument.
Risk perceptions [54,63,71,75]		Personal risk estimation to develop a breast cancer. It could also include “perceived susceptibility” from the Health Belief Model (ie, beliefs about the chances of experiencing a risk or getting a condition or disease) [91]. In the randomized controlled trial by Lee et al [54], risk perception was reported through “perceived susceptibility” from the HBM model. In 3 additional e-tools, all tailored-to-risk (Table 1), the authors evaluated women’s risk perceptions differently [63,71,75]. In Elkin et al’s [75], the participants were all at low-to-average risk based on their personalized risk estimates; the authors assessed the rates of women with this adequate “accurate” perception of their own risk. To evaluate the 2 other e-tools, instruments were adapted from previous studies/scales. Participants were asked to evaluate their chance of getting breast cancer as a percentage (0% being no chance and 100% being sure of getting cancer) [71], or as frequency (ie, a number out of 1000) [63,71]. The difference between the risk perception evaluated by the women itself and objective risk estimates was measured and reported by the authors, with a decrease of difference reflecting an increase in women’s accurate perception of risk.
**Quality of decision**		
	Regret [63,70]	The effect of the e-tool was evaluated on decision regret [70] or on anticipated regret [63]. Decision regret describes regret associated with self-recrimination around having made a bad decision or regret associated with the knowledge that another choice would have resulted in a better outcome. Anticipated regret measure is future oriented; anticipated inaction regret was associated with engaging in protective health behaviors, while anticipated action regret was associated with nonengaging. Findings suggest that anticipated inaction regret is more strongly felt and has more reliable associations with behavioral intentions and health behaviors.
	Decisional conflict [55,63,70,72,74,75]	Personal uncertainty about which option to choose. Decisional conflict is a multidimensional outcome measuring either 4 dimensions (ie, being “SURE^d^”) or 5 (previous dimensions plus “effective decision-making”), and measured with the SURE scale and the Decisional Conflict Scale, respectively. These 2 instruments are validated and negatively correlated scales [92]. With the SURE scale, a score of ≤3 indicates decisional conflict and a score equal to 4 an absence of conflict; the authors reported the number of women with a decisional conflict. This instrument was used in 2 randomized controlled trials [55,70]; whereas in the study by Reder and Kolip [70], the instrument was specifically about the intention to undergo breast cancer screening, this was not the case in the study by Roberto et al’s [55] in which the instrument was more general and about “decision.” The Decisional Conflict Scale used was either the low literacy version (ie, including 10 questions with 3 response categories) [72,74] or the traditional one (ie, including 16 statements with 5 response categories) [63,75]; Decisional Conflict Scale scoring ranged from 0 (no decisional conflict) to 100 (ie, extremely high decisional conflict or poor decision process); means of scoring was reported to reflect the decisional conflict.
	Informed choice [55,69,70]	Based on Marteau et al’s [16] definition: “an informed choice to undergo a screening test occurs when an individual has a positive attitude towards undergoing a test, has relevant knowledge about the test and undergoes it. An informed choice to decline a test occurs when an individual holds a negative attitude towards undergoing a test, has relevant knowledge about the test and does not undergo it. The choices that occur when individuals do not have relevant knowledge or when their attitudes are not reflected in their behavior, are uninformed.” Following Marteau et al’s [16] definition, only 1 study [70] reported informed choice regarding participation in breast cancer screening. Informed choice was usually reported toward intention to undergo breast cancer screening [55,69,70]. “Informed choice about intention” was then reported in our meta-analysis as the measure of informed choice. Except for Roberto et al [55], results regarding informed choice were reported in the original scientific publications only for participants who had made a decision about their intention to participate, excluding those who remained undecided [69,70]. This was made to strictly follow Marteau et al’s [16] definition (ie, measure of informed choice only among those who made either a positive or a negative decision). In our meta-analysis, we reported informed choice over the total number of women for whom data related to informed choice measurement (ie, knowledge, attitude, and intention) were available.
Shared decisions and communications with health professionals [64,67,72,75]		Eden et al [72] assessed the readiness of women to make a shared decision using the Graham Preparation for Decision Making scale. A higher level of scoring indicated that the women felt the decision aid prepared them to make a shared decision with clinical providers. Among the 22 e-tools, including the 6 identified as preparing for or facilitating shared decision-making [63,64,72-75], none was evaluated against whether a shared decision-making discussion occurred. Instead, the tools were assessed by measuring women’s readiness to discuss mammography [64], whether this discussion occurred [64,75], or the characteristics of the discussion including its quality [67]. The perspective of the health professionals was also evaluated regarding the 2 latest variables [67,75].
Other outcomes		Few studies assessed outcomes related to the stage of adoption or stage of decision other than behavior or intention, such as “having made a decision” [70] or “readiness to be tested” [64]; data were not reported here. Perceived quality of life was reported in 1 study [65]. Satisfaction with the decision was not reported. Among implementation outcomes, “Reach” (ie, how the tool reached target populations) was the most reported after efficacy/effectiveness (detailed above) and through a variety of instruments.

^a^The outcomes are reported in the following order: first, we present participation in breast cancer screening (behavior), followed by related determinants/factors based on the main theories, models, and concepts used to explain or understand cancer screening behavior [89,91]. Among those, intention, identified as the most proximal determinant of behavior in the Theory of Planned Behavior [89], is reported first. Next, we include outcomes related to the “quality of decision,” such as “regret” and the composite outcomes “decisional conflict” and “informed choice.” The outcomes reported last are those that were less frequently assessed in the included studies.

^b^Although reported by the authors, we did not include some studies; the reasons are detailed in Part B in Appendix S3 in Multimedia Appendix 1.

^c^“Instruments” were any item (eg, scale, questionnaire, or medical records) used to measure the outcomes.

^d^SURE: Sure of myself (Uncertainty), Understand information, Risk-benefit ratio (value clarity), and Encouragement.

#### Participation in BCS (Behavior)

Our meta-analysis found that e-tools, including a mix of tailored-not-to-risk e-tools (ie, tailored e-tools but not to the individual’s breast cancer risk; n=4) and features-with-tailoring tools (n=2), did not significantly increase women’s participation in BCS up to 6 months after using the e-tools (RR 1.09, 95% CI 0.97-1.23, *P*=.16, *I*^2^=78%, RE model) compared with the control group, which included either usual care [54,56,57,61,70] or a control website [55] (see Figure S1A in Multimedia Appendix 1). Excluding Lee et al’s study [54] significantly reduced heterogeneity (see the “Methods” section) without altering the overall pooled result (RR 1.03, 95% CI 0.98-1.08, *P*=.28, *I*^2^=14%, FE model; see Figure S1B and S1C in Multimedia Appendix 1). No subgroup differences were observed between tailored and features-with-tailoring e-tools (*χ*^2^_1_=1.2, *P*=.28; see Figure S1D in Multimedia Appendix 1). Two interventional studies without concurrent controls that assessed tailored-not-to-risk e-tools reported an increase in BCS participation among users compared with nonusers [59,67].

**Figure 2:**
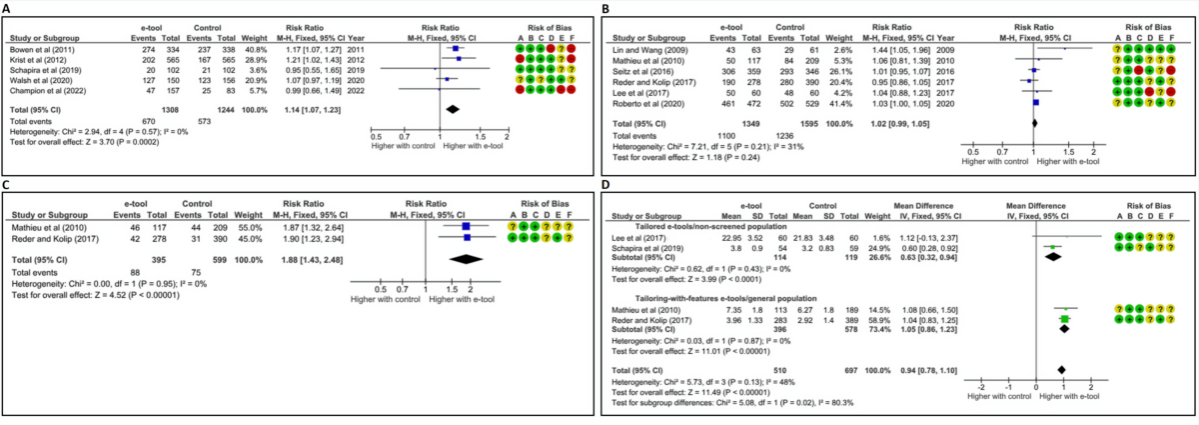
Forest plots of studies comparing the effect of e-tools with that of control on women’s (A) participation in breast cancer screening (BCS) by mammography at long term [61,63-66], (B) intention to undergo BCS [54,55,68-71], (C) intention *not* to undergo BCS [69,70], and (D) adequate knowledge about BCS [54,63,69,70]. We reported in (B) the data corresponding to Lin et al.’s “tailored message intervention” (TMI) [68] and Seitz et al.’s “extended information with untailored exemplars” e–tools [71]. Data for the other Lin et al.’s [68] and Seitz et al’s [71] e-tools are presented in in Appendix S4 in Multimedia Appendix 1. In (D) subgrouping was based on tailoring complexity of the e–tools (ie, tailored e-tools compared with features–with–tailoring e–tools), which end up being identical to subgrouping based on previous breast cancer screening status (ie, nonparticipants in BCS [tailored e–tools] compared with general population [features–with–tailoring e–tools]). Control was usual care, except in the studies by Lin and Wang [68], Roberto et al [55], and Seitz et al [71] (website), and Walsh et al [64] (video). The risk of bias was assessed using the Risk-of-Bias 2 (RoB 2) tool [52]. The risk level for each domain of RoB 2 (A: randomization process, B: deviations from the intended protocol, C: missing data, D: outcome measurement, and E: reporting results) and the (F) overall risk were evaluated as low (green), moderate (yellow), or high (red). M-H: Mantel-Haenszel.

Our meta-analysis further demonstrated that e-tools (n=5), all tailored to either risk (n=2) or other variables (n=3), increased women’s participation in BCS assessed at long-term follow-up (12-16 months after tool use; RR 1.14, 95% CI 1.07-1.23, *P*<.001, *I*^2^=0%, FE model) compared with the control group, which included usual care [61,63,65,66] or a control video [64] (Figure 2A). Subgroup analysis indicated a significant difference in effect between tailored-to-risk e-tools and other tailored e-tools, while highlighting a high heterogeneity within one subgroup (*χ*^2^_1_=23.54, *P*<.001, risk-based group: *I*^2^=0%, non-risk–based group: *I*^2^=98%; see Figure S2 in Multimedia Appendix 1).

#### Intention

Our meta-analysis, which incorporated 6 RCTs, showed that a mix of tailored (n=3, with 2 not based on risk) and features-with-tailoring (n=3) e-tools did not affect the rate of women intending to undergo BCS (RR 1.02, 95% CI 0.99-1.05, *P*=.24, *I*^2^=31%, FE model) compared with the control group, which included either usual care [54,69,70] or a control website [55,68,71] (Figure 2B; also see Appendix S4 in Multimedia Appendix 1). No subgroup differences were observed between tailored and features-with-tailoring e-tools (*χ*^2^_1_=0.6, *P*=.43; see Figure S3 in Multimedia Appendix 1). Similarly, no significant difference in intention was reported in 2 pre-post studies evaluating tailored-to-risk e-tools [38,72,74]. In 2 other studies with no control and small sample sizes (N=8 or 49), participants either expressed an intent to undergo BCS [60] or showed no change in intention [73].

Moreover, in 2 RCTs [69,70], the effect of 2 features-with-tailoring e-tools on the rate of women with negative intention (willingness not to undergo BCS) was assessed. Our meta-analysis showed that these tools increased the rate compared with usual care (RR 1.88, 95% CI 1.43-2.48, *P*<.001, *I*^2^=0%, FE model; Figure 2C).

#### Knowledge

One RCT evaluating a tailored e-tool reported a positive correlation between increased knowledge and participation in BCS [65]. Our meta-analysis, conducted with 5 RCTs assessing a mix of tailored (n=3) and features-with-tailoring (n=2) e-tools, demonstrated that the e-tools increased women’s adequate knowledge about BCS (MD 0.75, 95% CI 0.31-1.19, *P*<.001, *I*^2^=89%, RE model; see Figure S4A in Multimedia Appendix 1) compared with the control group, which consisted of either usual care [54,63,69,70] or a control website [55]. We conducted subanalyses to explore the sources of heterogeneity, with all analyses not altering the overall result (see Figure S4B and S4C in Multimedia Appendix 1). Excluding Roberto et al’s [55] study (see the “Methods” section) and subgroup analysis based on the degree of tailoring of the e-tools successfully reduced heterogeneity. Our findings indicated that adequate knowledge was higher with the features-with-tailoring e-tools, which were designed and tested with the general population, compared with the tailored e-tools, which were designed for and tested with women who were nonparticipants in BCS (*χ*^2^_1_=5.1, *P*=.02; Figure 2D) [54,63,69,70]. In a pre-post study [62], a significant increase in adequate knowledge was reported with 1 features-with-tailoring e-tool (*P*=.001). For 2 tailored e-tools, with no reported control group, 48%-64% of users indicated increased knowledge or reported receiving new information, while approximately 15% either disagreed or had some level of disagreement with that statement [64,67,77].

#### Attitudes

Our meta-analysis showed that features-with-tailoring e-tools, compared with the control group (either usual care [69,70] or a control website [55]), had no significant effect on the rate of women with positive attitudes toward undergoing BCS (ie, being favorable to undergo BCS; RR 0.98, 95% CI 0.95-1.01, *P*=.19, *I*^2^=0%, FE model; see Figure S5 in Multimedia Appendix 1). In another RCT with a 2×2 design that tested a features-with-tailoring e-tool featuring a virtual AI physician, the communication style of the physician and whether the women’s needs were made salient were found to influence women’s attitudes toward undergoing BCS [58].

#### Self-Efficacy

The impact of e-tools on women’s level of self-efficacy regarding BCS was reported for 2 tailored e-tools. One study, an RCT, found no significant difference in self-efficacy compared with a brochure [54]. By contrast, a pre-post study [72] demonstrated a significant increase in self-efficacy.

#### Worry

**Figure 3:**
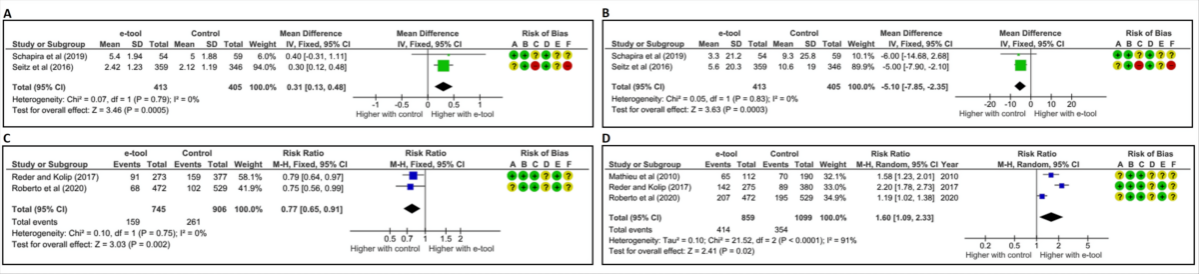
Forest plots of studies comparing the effect of e-tools with that of control on women's (A) level of worry [63,71], (B) accuracy of perception of individual breast cancer risk [63,71], (C) decisional conflict [55,70], and (D) informed choice [55,69,70]. We reported in (A) and (B) the data corresponding to Seitz et al’s [71] “extended information with untailored exemplars” e-tool, which was the most comparable to Schapira et al’s [63] e-tool. Data for the other Seitz et al’s [71] e-tools are presented in Figures S6 and S7 in Multimedia Appendix 1. The control group was usual care, except for Seitz et al’s [71] and Roberto et al’s [55] e-tools, which used a website as the control. The risk of bias was assessed using the Risk-of-Bias 2 (RoB 2) tool [52]. The risk level for each domain of RoB 2 (A: randomization process, B: deviations from the intended protocol, C: missing data, D: outcome measurement, and E: reporting results) and the (F) overall risk were evaluated as low (green), moderate (yellow), or high (red). M-H: Mantel-Haenszel.

Emotional changes, including anxiety, worry, or fear, were assessed either in general [69,71,78] or specifically related to breast cancer [63,67,75] (Table 2). Our meta-analysis, conducted with 2 RCTs evaluating tailored-to-risk tools, showed that compared with the control group (usual care [63] or a control website [71]), the e-tools increased women’s level of worry (MD 0.31, 95% CI 0.13-0.48, *P*<.001, *I*^2^=0%, FE model; Figure 3A), irrespective of the complexity of the tailored messages (see Figure S6 in Multimedia Appendix 1). In a prospective single-arm study, a tailored-to-risk tool that predicted breast cancer risk for women also increased their frequency of worry, which correlated with their level of risk (*P*<.01 by analysis of variance) [75]. In 2 other studies not comparing with controls [67,69], women reported not being particularly worried about breast cancer after using a features-with-tailoring tool [69]. Additionally, 80.9% of users of a tailored-not-to-risk tool indicated that the clinician helped reduce their fears and worries [67].

#### Perceptions of Risk of Breast Cancer

Two tailored-to-risk e-tools were evaluated for their effect on women’s accuracy in perceiving their breast cancer risk. Accuracy was determined by measuring the difference between the risk perception assessed by the woman herself and her objective risk estimates provided by the e-tool. Our meta-analysis showed a significant decrease in this difference, indicating that women’s accurate estimation of breast cancer risk improved with the use of the e-tools compared with the control group, which consisted of usual care [63] or a control website [71]. A significant improvement in the accuracy of breast cancer risk perception was observed when extended information with untailored examples was used in the e-tool (MD –5.10, 95% CI –7.85 to 2.35, *P*<.001, *I*^2^=0%, FE model; Figure 3B), but not with other types of tailored messages (see Figure S7 in Multimedia Appendix 1). In another study using a tailored-to-risk tool without a control group, 70% of users reported having an “accurate perception” of their breast cancer risk, correctly identifying themselves as at low or average risk [75]. Additionally, the perceived susceptibility of breast cancer was assessed using a tailored-not-to-risk e-tool, which showed no significant difference compared with a control brochure (*F*_1,118_=0.73, *P*=.01, effect size = 0.01) [54].

#### Regret

One RCT [70] assessed women’s regret about their decision to undergo BCS. The study found no significant difference in regret between the group using the features-with-tailoring e-tool and the usual care group. Additionally, anticipated regret for decisions to delay or initiate mammography was evaluated using a tailored-to-risk e-tool in another RCT [63], which also showed no significant difference compared with usual care.

#### Decisional Conflict

Our meta-analysis, which included 2 RCTs comparing 2 features-with-tailoring e-tools with either usual care [70] or a control website [55], found that the e-tools significantly reduced the rate of women experiencing decisional conflict (ie, uncertainty regarding the choice to be made or the decision to undergo BCS). The results showed an RR of 0.77 (95% CI 0.65-0.91, *P*=.002, *I*^2^=0%, FE model; Figure 3C).

Other results obtained with tailored-to-risk tools were more mixed [63,72,74,75]. One RCT reported no change in the average decisional conflict (RR –0.34, 95% CI –0.71 to 0.03, *P*=.07) [63]. For 2 other e-tools tested through pre-post study designs [72,74], Yu et al [38] reported a significant decrease in decisional conflict. In a prospective single-arm study, women were found not to have difficulties in implementing decisions, as indicated by their average decisional conflict score [75].

#### Informed Choice

Our meta-analysis showed that, compared with the control group (either usual care [69,70] or a control website [55]), 3 features-with-tailoring tools increased the rate of women who made an informed choice regarding their intention to undergo BCS by mammography (RR 1.60, 95% CI 1.09-2.33, *P*=.02, *I*^2^=91%; RE model; Figure 3D). Subanalyses to reduce heterogeneity were conducted but did not yield a significant decrease (data not shown).

#### Shared Decision-Making and Communications With Health Professionals

The effect of the tools on discussions about BCS during appointments between HPs and women was evaluated with 4 tailored tools [64,67,72,75]. One study reported that an additional 17.2% of women discussed mammography with their HPs compared with a control video group (*P*<.01) [64]. Another study found that women felt well-prepared to make shared decisions with clinical providers [72]. In 2 other studies comparing e-tool users with nonusers, HPs reported no significant difference in the rates of mammography discussions (90% vs 92%) [75], and the rates of medical “wellness” appointments did not increase (16.3% vs 21.5%) [67].

#### Grading of the Available Evidence

We summarized and graded the evidence detailed above in Table 3.

With a moderate to high level of certainty, the evidence supports the findings that e-tools (1) increased women’s long-term participation in BCS, intention not to participate, adequate knowledge, worry, and informed choice, while decreasing decisional conflict, and (2) had no effect on short-term participation in BCS, intention, and positive attitudes toward undergoing BCS (Table 3). Additionally, evidence indicates that the tailoring nature of the e-tools is the most influential factor in driving their effectiveness (Table 3).

**Table 3:** Grade summary of certainty of evidence related to the outcomes used to assess the effects of e-tools.

Outcome and e-tool effect (compared with control)		Main source of evidence (number of e-tools and type of control)	Certainty of evidence (GRADE^a^)^b^ (reasons for downgrading)	E-tool or population main characteristics
Participation at short term in BCS^c^				
	No effect	Meta-analysis with RCTs^d^ (5 tools, control was either usual care [4 tools] or website [1 tool])	⊗⊗⊗○ Moderate^e^ (downgrading due to the presence of 2 high-risk of bias studies in the meta-analysis)	Mix of tailored-not-to-risk and features-with-tailoring e-tools, no differences between the 2 types of e-tools
Participation at long term in BCS^c^				
	Increased	Meta-analysis with RCTs (5 tools, control was either usual care [4 tools] or video [1 tool])	⊗⊗⊗○ Moderate^e^ (downgrading due to 3 high-risk of bias RCTs)	All tailored tools, but not only based on risk. Results suggest a difference between tailored-to-risk and other tailored e-tools
Intention to undergo BCS^c^				
	No effect	Meta-analysis with RCTs (6 tools, control was either usual care [3 tools] or website [3 tools]) Yu et al’s [38] meta-analysis with pre-post study design (2 tools)	⊗⊗⊗⊗ High^f^	Mix of tailored and features-with-tailoring e-tools, no differences between the 2 types of tools, further suggested by Yu et al’s results with 2 tailored-to-risk e-tools
Intention not to participate (negative intention) in BCS^c^				
	Increased	Meta-analysis with RCTs (2 tools, control was usual care [2 tools])	⊗⊗⊗○ Moderate^e^ (downgrading due to very low number of RCTs [2 studies])	Features-with-tailoring e-tools
Adequate knowledge				
	Increased	Meta-analysis with RCTs (4 tools, control was usual care [4 tools])	⊗⊗⊗⊗ High^f^	The increase is independent of whether the e-tool is tailored or featured-with-tailoring; however, adequate knowledge is higher with features-with-tailoring e-tools assessed with the general population compared with tailored tools assessed with the nonscreened population
Positive attitude				
	No effect	Meta-analysis with RCTs (3 tools, control was usual care [2 tools] or website [1 tool])	⊗⊗⊗⊗ High^f^	Features-with-tailoring e-tools
Self-efficacy				
	No effect	1 RCT and 1 pre-post study	⊗○○○ Very low^g^ (downgrading due to very low number of studies, contradictory results obtained with 1 RCT and 1 pre-post study)	Tailored e-tools
Worry				
	Increased	Meta-analysis with RCTs (2 tools, control was either usual care [1 tool] or website [1 tool]) Prospective single-arm study showing correlation with risk and worry [75]	⊗⊗⊗○ Moderate^e^ (downgrading due to low number of RCTs [2 studies] including 1 with high bias; upgrading due to correlation result)	Tailored-to-risk e-tools
Accurate risk perception				
	Increased	Meta-analysis with RCTs (2 tools, control was either usual care [1 tool] or website [1 tool])	⊗⊗○○ Low^h^ (downgrading due to low number of RCTs [2 studies], including 1 with a high risk of bias)	Tailored-to-risk e-tools but with specific types of tool messages. The increase seems to be specific to tailored-to-risk e-tools as no effect on perceived susceptibility was obtained with tailored-not-to-risk e-tool
Regret (decision regret or anticipated regret)				
	No effect	1 RCT for each type of regret (1 tool, control was usual care)	⊗○○○ Very low^g^ (downgrading due to very low number of RCTs [n=1] for each type of regret)	1 features-with-tailoring and 1 tailored e-tool
Decisional conflict				
	Decreased	Meta-analysis with RCTs (2 tools, control was either usual care [1 tool] or website [1 tool])	⊗⊗⊗○ Moderate^e^ (downgrading due to 2 RCTs only)	Features-with-tailoring e-tools
	No effect	1 RCT and Yu et al’s [38] meta-analysis with a pre-post study design (2 tools)	⊗○○○ Very low^g^ (downgrading due to the very low number of RCTs [1 study] and contradictory results with Yu et al’s [38] meta-analysis with pre-post studies)	Tailored-to-risk e-tools
Informed choice about the intention to undergo BCS^c^				
	Increased	Meta-analysis with RCTs (3 tools, control was either usual care [2 tools] or website [1 tool])	⊗⊗⊗○ Moderate^e^ (downgrading due to inconsistency, ie, high *I*^2^)	Features-with-tailoring e-tools
Discussion/SDM^i^				
	Increase women’s discussion about mammography	1 RCT 1 tool	⊗○○○ Very low^g^ (Downgrading due to the very low number of RCT^d^ [1 study])	1 tailored e-tool
	No evidence about SDM	Not applicable	○○○○ No formal evidence	Not applicable

^a^GRADE: Grading of Recommendations, Assessment, Development, and Evaluation.

^b^We adopted the GRADE Working Group methodology of grading evidence [49-51]. Our baseline statement was high for meta-analyses with RCTs (our results), moderate for RCTs without meta-analysis, and low for non-RCTs. Where indicated, we decreased the certainty in the evidence to at least one level based on the assessment of the following domains: risk of bias, imprecision (number of studies, SD), inconsistency (inconsistent effect between several studies, *I*^2^), indirectness, and publication bias; additionally, downgrading and reasons why it was performed are indicated. As a result of the low total number of studies selected in this review, we did not downgrade based on imprecision (number of studies, SD) criteria except where only 2 studies were available and as detailed in the table. We graded evidence as in footnotes e, f, g, and h.

^c^BCS: breast cancer screening

^d^RCT: randomized controlled trial.

^e^Moderate certainty: we are moderately confident in the effect estimate: the true effect is likely to be close to the estimate of the effect, but there is a possibility that it is substantially different.

^f^High certainty: we are very confident that the true effect lies close to that of the estimate of the effect.

^g^Very low certainty: we have very little confidence in the effect estimate; the true effect is likely to be substantially different from the estimate of effect.

^h^Low certainty: our confidence in the effect estimate is limited; the true effect may be substantially different from the estimate of the effect.

^i^SDM: shared decision-making.

## Discussion

### Principal Findings

This is the first systematic review to provide a comprehensive overview of the effects of e-tools on women’s decision-making about BCS. The findings indicate that e-tools increase women’s long-term participation in BCS, intention not to participate, adequate knowledge, worry, and informed choice. They also reduce women’s decisional conflict. However, e-tools have no effect on women’s short-term participation in BCS, intention, or positive attitudes toward undergoing BCS. Additionally, the review identifies variables that could influence these effects. Among all the variables explored, the degree of tailoring in e-tools (ie, whether the tools were fully “tailored” or “featured with tailoring”) appeared to be the most influential (Table 3).

While all e-tools were shown to increase knowledge about BCS, the extent of the increase depended on the degree of tailoring or the study population. The increase was greater with “featured-with-tailoring” e-tools assessed in the general population than with tailored e-tools assessed in non-BCS participants. However, the observation that 1 tailored e-tool was more effective in less-educated women [57,79] supports the notion that the degree of tailoring plays a key role in driving a greater or lesser effect on knowledge. Our results suggest that complex e-tools (ie, tailored tools) are not necessarily more effective in ensuring that women are adequately informed about BCS than less complex e-tools (ie, features-with-tailoring). This finding aligns with recent studies, particularly those involving women from low socioeconomic backgrounds, which indicate that complex information about cancer screening may not be essential for women to be adequately informed [93,94].

Our results indicate that features-with-tailoring e-tools influence women’s decisions about undergoing BCS. These tools reduce decisional conflict regarding BCS as well as increase both the intention not to undergo BCS and informed choice about this intention (Table 3). However, they have no effect on positive attitudes toward BCS. The reduction in decisional conflict concerning BCS aligns with findings from a previous meta-analysis of web-based tools [38]. By contrast, a meta-analysis that included both e-tools and printed DAs did not report this effect [23], which may be attributable to the quality of one of the assessed printed DAs [95,96]. An increase in women’s informed choice regarding BCS has been consistently reported in all meta-analyses conducted to date on DAs used for BCS, regardless of whether the tools were web-based or printed [23,24,38]. Similar to our findings, these reported effects were associated with features-with-tailoring tools, as defined in our study [23,24,38]. However, a more detailed analysis of our results suggests that this increase in informed choice may stem from a rise in both negative intentions and negative attitudes toward BCS. As defined by Marteau et al [16], individuals are considered to have made an informed choice about BCS if they possess adequate knowledge and hold either positive attitudes and intentions or negative attitudes and intentions. Our results showed that while the e-tools by Reder and Kolip [70], Mathieu et al [69], and Roberto et al [55] increased informed choice (Figure 3D) and knowledge (see Figure S8A in Multimedia Appendix 1), they did not increase either positive attitudes toward BCS (see Figure S5 in Multimedia Appendix 1) or the intention to undergo BCS (see Figure S8B in Multimedia Appendix 1). It is likely that the observed increase in informed choice among women using these e-tools was driven by an increase in negative intentions, as evidenced by 2 of the 3 tools (Figure 2C), and possibly by negative attitudes toward participation in BCS, as reported in other studies [24]. Interestingly, these 3 e-tools are among the highest-rated BCS DAs based on the IPDAS criteria. This was formally evaluated for the e-tools by Reder and Kolip [70] and Mathieu et al [69] in a recent systematic review [85]. Although Roberto et al’s [55] e-tool was not formally assessed in the same review, it is expected to be of similarly high quality due to its strong resemblance to Reder and Kolip’s [70] tool [85]. Our results suggest that even high-quality e-tools, as assessed by IPDAS criteria, and their ability to achieve higher levels of adequate knowledge, increase informed choice, and reduce decisional conflict, do not guarantee the promotion of positive intentions and attitudes toward undergoing BCS. On the contrary, these tools may increase negative intentions and attitudes. BCS programs should take this finding into consideration.

Our results on participation in BCS (Table 3) strongly suggest that only tailored e-tools have a significant effect on participation. Two factors may explain this finding regarding tailored-to-risk e-tools. First, these e-tools, when appropriately designed, increase women’s accurate perception of their own breast cancer risk (as shown by our results). Second, they may also heighten women’s levels of worry (Table 3), which is likely a key driver of increased participation in BCS. Indeed, while fear of breast cancer has been reported as a barrier, it has also been identified as a facilitator of BCS participation [9,10,97]. BCS programs should address women’s feelings of worry if they aim to implement tailored-to-risk e-tools. However, further studies are needed to assess women’s acceptability of feeling worried in comparison to their perceived benefits of using these e-tools. In addition, it is important to note that the tailored e-tools evaluated in this review incorporate multiple features that allow for their effective integration into clinical pathways or health care services (Table 1). These features can be seen as opportunities to reduce barriers and increase access to BCS participation, such as by facilitating helpful discussions with health care providers (HPs) or simplifying the appointment process [9,10,97]. Additionally, these features could help mitigate the impact of the tools on increasing worry [67].

Among the 22 e-tools investigated in this research, including the 6 identified as preparing for or facilitating SDM [63,64,72-75], none were evaluated using any validated SDM instruments [98-106]. There was no evidence to indicate whether the e-tools had an effect on SDM. Although this review suggests that e-tools, particularly tailored e-tools, may improve SDM by enhancing the quality of appointments with health care providers (HPs), this is likely due to their multiple features, which enable effective integration into clinical pathways or health care services (Table 1).

### Recommendations for Future Developments

While all types of e-tools appeared effective in increasing users’ knowledge about BCS, our results highlight important dilemmas for BCS programs that are using or planning to use e-tools to support women at average risk of breast cancer [2-4] in making decisions about BCS. Although features-with-tailoring e-tools can potentially increase informed choice and reduce decisional conflict, they may also be perceived as negatively impacting BCS programs by fostering negative intentions and attitudes toward undergoing BCS. Conversely, tailored e-tools, despite lacking evidence to support their effects on informed choice and decisional conflict, would increase women’s participation in BCS but, when tailored to risk, these tools may also heighten their levels of worry. One potential approach to minimize risks to both women’s well-being (worry) and BCS programs would be an “on-demand” model. In this model, women would “tailor” their own information needs, deciding on the nature and amount of information they feel is necessary. In this approach, we recommend that (1) all women be provided with a minimum of important, carefully assessed information about BCS in both text and audio formats, with multiple language options; and (2) women who do not wish to participate in BCS be given the option, through the e-tool, to obtain estimates of their breast cancer risk or, at the very least, to have a discussion with health care providers (HPs) about breast cancer risk in general. Some women may be unaware of the average breast cancer risk for women or of their own increased risk [2-4]. We also suggest that e-tools be well integrated into clinical pathways or health care services in various ways to reduce access-related barriers, especially those specific to low socioeconomic groups, such as health literacy. Notably, embedding e-tools in a health portal (Table 1) would facilitate appointments and communication with health care providers (HPs). AI tools, which have not been fully explored in the context of BCS according to this review, could support the “on-demand” approach. However, both the efficacy of such AI-based tools and potential ethical issues or biases need to be carefully assessed [107-110].

### Limitations

This study has several limitations that should be considered in future research. Caution is needed when generalizing our results due to (1) the limited number of studies included, particularly in the meta-analyses, with some outcomes assessed by only 2 RCTs (eg, “intention not to undergo BCS,” “level of worry,” “accuracy of perception of individual breast cancer risk,” and “decisional conflict”), some of which were at high risk of bias; (2) the high heterogeneity between the studies and e-tools; (3) the fact that almost all studies were conducted in high-income countries, mostly in the United States, which may limit the applicability of the results to other settings or low- and middle-income countries; and (4) the lack of assessment of implementation factors (eg, comparing users vs nonusers or completers vs noncompleters) and their impact on decision-making outcomes. Additionally, (1) we did not thoroughly review the content of the BCS information provided in the e-tools evaluated in this review, so we cannot draw conclusions about the tangible value of the increase in knowledge [95]; and (2) we did not have enough information to determine whether some of the e-tools could be classified as DAs. However, we applied a robust methodology to minimize some of these limitations, notably by comprehensively exploring the variables that could explain the heterogeneity of the results, using RoB 2 and GRADE assessments. A review is currently underway to explore implementation outcomes.

### Conclusions

Although no evidence was available to assess the efficacy of the tools in supporting SDM, this review demonstrates that the e-tools designed to assist women’s decision-making regarding BCS do impact this process. The degree of tailoring of the e-tools (ie, whether they are tailored, particularly to risk, or include features with tailoring) appears to be the most significant factor influencing decision-making. This review provides valuable insights for BCS programs when implementing such e-tools and offers directions for future development.

## Appendix

Multimedia Appendix 1 PRISMA (Preferred Reporting Items for Systematic Reviews and Meta-Analyses) checklist and additional methods and analysis.

## Abbreviations

AI: artificial intelligence
BCS: breast cancer screening
DA: decision aid
FE: fixed effect
GRADE: Grading of Recommendations, Assessment, Development, and Evaluation
HP: health professional
IPDAS: International Patient Decision Aid Standards
MD: mean difference
PRISMA: Preferred Reporting Items for Systematic Reviews and Meta-Analyses
PROSPERO: International Prospective Register of Systematic Reviews
RCT: randomized controlled trial
RE: random effect
RoB 2: Risk-of-Bias 2
RR: relative risk/risk ratio
SDM: shared decision-making
SWiM: synthesis without meta-analysis

## Data Availability

Multimedia Appendix 1, which cites additional references [111-115], contains extra information about the methods used and reports additional meta-analyses and subanalyses. The complete qualitative data sets generated or analyzed during this study are available from the corresponding author upon reasonable request.
