# Supplementary material for: Impact of Online Interactive Decision Tools on Women’s Decision-Making Regarding Breast Cancer Screening: Systematic Review and Meta-Analysis
Source: J Med Internet Res. 2025 Jan 29;27:e65974. doi: 10.2196/65974 (PMC11822326; doi:10.2196/65974)
Supplement: Multimedia Appendix 1 [file jmir_v27i1e65974_app1.pdf]

This is a Multimedia Appendix to a full manuscript published in the J Med Internet Res. For full copyright and citation information see http://dx.doi.org/10.2196/jmir.65974

Note: The references cited in this “Multimedia Appendix 1” and listed page 48, are numbered as in the full JMIR manuscript.

Appendix S1: Databases search criteria. **Page 3**

Appendix S2: Data extraction. **Page 10**

Appendix S3: Supplementary information regarding meta-analyses. **Page 12**

Appendix S4: Intention, exploration of effects of variables and other Lin and Wang [68] and Seitz et al’s [71] e-tools. **Page 14**

Table S1. PRISMA 2020 checklist. **Page 16**

Table S2. Characteristics of the included studies. **Page 21**

Table S3. Characteristics of the study populations. **Page 29**

Table S4. Detailed characteristics of the e-tools. **Page 31**

Figure S1. Participation in breast cancer screening (BCS) assessed at short-term. **Page 36**

Figure S2. Participation in BCS assessed at long-term: effect of subgroup analysis. **Page 38**

Figure S3. Intention subgroup analysis (ie, tailored tools vs features-with-tailoring e-tools). **Page 39**

Figure S4. Adequate knowledge. **Page 40**

Figure S5. Attitudes: rates of women with positive attitude toward undergoing BCS. **Page 41**

Figure S6. Worry when using different Seitz et al's e-tools/tailored messages [71]. **Page 42**

Figure S7. Accuracy of risk when using different Seitz et al's e-tools/tailored messages [71]. **Page 44**

Figure S8. Informed choice-related dimensions. Meta-analyses were conducted with the e-tools shown to increase informed choice, to evaluate their effects on adequate knowledge and positive intention to undergo BCS. Effect of those e-tools on positive attitudes toward undergoing BCS are reported in Figure S5. **Page 46**

References **Page 48**

Appendix S1: Databases search criteria

Last update 10 April 2023

<u>MEDLINE VIA pubmed</u>

(((“breast cancer”[Title/Abstract] OR “breast cancers”[Title/Abstract] OR “breast tumor”[Title/Abstract] OR “breast tumors”[Title/Abstract] OR “breast tumour”[Title/Abstract] OR “breast tumours”[Title/Abstract] OR “breast carcinoma*”[Title/Abstract] OR “mammary cancer”[Title/Abstract] OR “mammary cancers”[Title/Abstract] OR “breast neoplasm*”[Title/Abstract] OR “mammary neoplasm*”[Title/Abstract] OR “mammary carcinoma*”[Title/Abstract] OR “mammary tumor”[Title/Abstract] OR “mammary tumour”[Title/Abstract] OR “mammary tumors”[Title/Abstract] OR “mammary tumours”[Title/Abstract] OR “breast neoplasms”[MeSH Terms]) AND (“Secondary Prevention”[MeSH Terms] OR “prevention”[Title/Abstract] OR “screening”[Title/Abstract] OR “early diagnosis”[Title/Abstract] OR “early detection*”[Title/Abstract] OR “mass screening”[MeSH Terms] OR “early detection of cancer”[MeSH Terms])) OR (“mammography”[MeSH Terms] OR “ultrasonography, mammary”[MeSH Terms] OR “mammo*”[Title/Abstract]))

AND (“decid*”[Title/Abstract] OR “decision*”[Title/Abstract] OR “choose”[Title/Abstract] OR “chosen”[Title/Abstract] OR “choosing”[Title/Abstract] OR “choice*”[Title/Abstract] OR “guide”[Title/Abstract] OR “preferen*”[Title/Abstract] OR “tailor*”[Title/Abstract] OR “personal*”[Title/Abstract] OR “option*”[Title/Abstract] OR “assist*”[Title/Abstract] OR “aid”[Title/Abstract] OR “help*”[Title/Abstract] OR “computer-assisted”[Title/Abstract] OR “decision support techniques”[MeSH Terms] OR “decision making”[MeSH Terms] OR “decision making, computer assisted”[MeSH Terms] OR “decision theory”[MeSH Terms] OR “risk assessment”[MeSH Terms] OR “decision support systems, clinical”[MeSH Terms] OR “decision making, shared”[MeSH Terms] OR “risks assessment”[Title/Abstract] OR “risks appraisal”[Title/Abstract] OR “risk assessment”[Title/Abstract] OR “risk appraisal”[Title/Abstract] OR “risk evaluat*”[Title/Abstract] OR “risk 3ide rag*”[Title/Abstract] OR “uncertainty”[Title/Abstract])

AND (“online”[Title/Abstract] OR “social media*”[Title/Abstract] OR “forum*”[Title/Abstract] OR “platform*”[Title/Abstract] OR “chat”[Title/Abstract] OR “chats”[Title/Abstract] OR “chatbot*”[Title/Abstract] OR “social network*”[Title/Abstract] OR “information technolog*”[Title/Abstract] OR “crowdsourcing”[Title/Abstract] OR “web”[Title/Abstract] OR “webinar*”[Title/Abstract] OR “website*”[Title/Abstract] OR “digital”[Title/Abstract] OR “mobile app*”[Title/Abstract] OR “e-health”[Title/Abstract] OR “eHealth”[Title/Abstract] OR “podcast*”[Title/Abstract] OR “messaging”[Title/Abstract] OR “eHealth”[Title/Abstract] OR “Skype”[Title/Abstract] OR “Facetime”[Title/Abstract] OR “Twitter”[Title/Abstract] OR “facebook”[Title/Abstract] OR “virtual”[Title/Abstract] OR “virtuali*”[Title/Abstract] OR “e-tool”[Title/Abstract] OR “eTool”[Title/Abstract] OR “web-based”[Title/Abstract] OR “information display*”[Title/Abstract] OR “computing method*”[Title/Abstract] OR “blog*”[Title/Abstract] OR “Data Display”[Title/Abstract] OR “mobile health”[Title/Abstract] OR “m-health”[Title/Abstract] OR “digital health”[Title/Abstract] OR “Internet”[Title/Abstract] OR “computer*”[Title/Abstract] OR “interactive”[Title/Abstract] OR “tablet”[Title/Abstract] OR “Patient Portals”[MeSH Terms] OR “Blogging”[MeSH Terms] OR “Internet”[MeSH Terms] OR “Social Networking”[MeSH Terms] OR “Information Technology”[MeSH Terms] OR “Data Display”[MeSH Terms] OR “Computing Methodologies”[MeSH Terms] OR “Online

Systems”[MeSH Terms] OR “telemedicine”[MeSH Terms] OR “DVD”[Title/Abstract]) AND (2022/10/1:2023/4/30[pdat])

EMBASE

‘breast cancer’:ti,ab OR ‘breast cancers’:ti,ab OR ‘breast tumor’:ti,ab OR ‘breast tumors’:ti,ab OR ‘breast tumour’:ti,ab OR ‘breast tumours’:ti,ab OR ‘breast carcinoma’:ti,ab OR ‘breast carcinomas’:ti,ab OR ‘mammary cancer’:ti,ab OR ‘mammary cancers’:ti,ab OR ‘breast neoplasm’:ti,ab OR ‘breast neoplasms’:ti,ab OR ‘mammary neoplasm’:ti,ab OR ‘mammary neoplasms’:ti,ab OR ‘mammary carcinoma*’:ti,ab OR ‘mammary tumor’:ti,ab OR ‘mammary tumour’:ti,ab OR ‘mammary tumors’:ti,ab OR ‘mammary tumours’:ti,ab OR ‘breast tumor’/exp OR breast*:ti,ab OR ‘breast’/exp

AND ‘secondary prevention’/exp OR prevention:ti,ab OR screening:ti,ab OR ‘early diagnosis’:ti,ab OR ‘early detection’:ti,ab OR ‘mass screening’/exp OR ‘early detection of cancer’/exp

OR ‘mammography’/exp OR ‘ultrasonography, mammary’/exp OR mammo*:ti,ab

AND

decid*:ti,ab OR decision*:ti,ab OR choose:ti,ab OR chosen:ti,ab OR choosing:ti,ab OR choice*:ti,ab OR guide:ti,ab OR preferen*:ti,ab OR tailor*:ti,ab OR personal*:ti,ab OR option*:ti,ab OR assist*:ti,ab OR aid*:ti,ab OR help*:ti,ab OR ‘computer assisted’:ti,ab OR ‘risks assessment’:ti,ab OR ‘risks appraisal’:ti,ab OR ‘risk assessments’:ti,ab OR ‘risk appraisals’:ti,ab OR (risk:ti,ab AND evaluat*:ti,ab) OR (risk:ti,ab AND 4ide rag*:ti,ab) OR uncertainty:ti,ab OR ‘decision support system’/exp OR ‘decision making’/exp OR ‘decision theory’/exp OR ‘risk assessment’/exp

AND

online:ti,ab OR ‘social media’:ti,ab OR forum*:ti,ab OR platform*:ti,ab OR chat:ti,ab OR chats:ti,ab OR chatbot*:ti,ab OR ‘social network’:ti,ab OR ‘social networks’:ti,ab OR ‘information technology’:ti,ab OR ‘information technologies’:ti,ab OR crowdsourcing:ti,ab OR web:ti,ab OR webinar*:ti,ab OR website*:ti,ab OR digital:ti,ab OR ‘mobile application’:ti,ab OR ‘mobile applications’:ti,ab OR ‘e-health’:ti,ab OR podcast*:ti,ab OR messaging:ti,ab OR ehealth:ti,ab OR skype:ti,ab OR facetime:ti,ab OR twitter:ti,ab OR facebook:ti,ab OR virtual:ti,ab OR virtuali*:ti,ab OR ‘e tool’:ti,ab OR etool:ti,ab OR ‘web based’:ti,ab OR ‘information display’:ti,ab OR ‘computing method’:ti,ab OR ‘computing methods’:ti,ab OR blog*:ti,ab OR ‘data display’:ti,ab OR ‘mobile health’:ti,ab OR ‘m-health’:ti,ab OR ‘digital health’:ti,ab OR internet:ti,ab OR computer*:ti,ab OR interactive:ti,ab OR tablet*:ti,ab OR ‘blogging’/exp OR ‘internet’/exp OR ‘social network’/exp OR ‘information technology device’/exp OR ‘information technology’/exp OR ‘computer analysis’/exp OR ‘online system’/exp OR ‘telemedicine’/exp OR telehealth:ti,ab OR mhealth:ti,ab OR telemonitoring:ti,ab OR interface*:ti,ab OR ‘educational technology’/exp OR ‘devices’/exp OR ‘devices’:ti,ab OR ‘DVD’:ti,ab

CINAHL

MM (“Computers and Computerization+”)) OR MM (“Social Media” OR “Social Networks” OR “Social Networking” OR Internet OR “Online Social Networking” OR “Web Browsers” OR “Mobile Applications” OR “Telehealth” OR Blogs OR Computer Communication Networks OR “Information Technology” OR Telecommunications OR Health Information Networks OR Online Systems OR Patient Portals) OR TI (online OR “social media*” OR forum* OR platform* OR chat OR chats OR chatbot* OR “social network*” OR “information technolog*” OR crowdsourcing OR web OR webinar* OR website* OR digital OR mobile app* OR e-health OR ehealth OR Podcast* OR messaging OR eHealth OR Skype OR Facetime OR Twitter OR facebook OR virtual OR virtuali* OR e-tool OR eTool OR “web-based” OR “information display*” OR “computing method*” OR blog* OR “data display” OR “mobile health” OR m-health OR “digital health” OR Internet OR computer* OR interactive OR tablet OR “Patient Portals” OR “Blogging” OR Internet OR “Social Networking” OR “Information Technology” OR “Data Display” OR “Computing Methodologies” OR “Online Systems” OR Telemedicine OR telehealth OR “information network*” OR DVD) OR AB (online OR “social media*” OR forum* OR platform* OR chat OR chats OR chatbot* OR “social network*” OR “information technolog*” OR crowdsourcing OR web OR webinar* OR website* OR digital OR mobile app* OR e-health OR ehealth OR Podcast* OR messaging OR eHealth OR Skype OR Facetime OR Twitter OR facebook OR virtual OR virtuali* OR e-tool OR eTool OR “web-based” OR “information display*” OR “computing method*” OR blog* OR “data display” OR “mobile health” OR m-health OR “digital health” OR Internet OR computer* OR interactive OR tablet OR “Patient Portals” OR “Blogging” OR Internet OR “Social Networking” OR “Information Technology” OR “Data Display” OR “Computing Methodologies” OR “Online Systems” OR Telemedicine OR telehealth OR “information network*” OR DVD)

AND

TI (decid* OR decision* OR choose OR chosen OR choosing OR choice* OR guide OR Preferen* OR tailor* OR personal* OR option* OR assist* OR aid* OR help*OR “computer-assisted” OR “risks assessment” OR “risks appraisal” OR “risk assessment” OR “risk appraisal” OR “risk evaluat*” OR “risk 5ide rag*” OR uncertainty OR “Decision Support Techniques” OR “Decision Making” OR “Decision Theory” OR “Decision Support Systems” OR “Shared Decision Making” OR Thinking OR “Risk perception” OR “attitude to risk*”) OR AB (decid* OR decision* OR choose OR chosen OR choosing OR choice* OR guide OR Preferen* OR tailor* OR personal* OR option* OR assist* OR aid* OR help*OR “computer-assisted” OR “risks assessment” OR “risks appraisal” OR “risk assessment” OR “risk appraisal” OR “risk evaluat*” OR “risk 5ide rag*” OR uncertainty OR “Decision Support Techniques” OR “Decision Making” OR “Decision Theory” OR “Decision Support Systems” OR “Shared Decision Making” OR Thinking OR “Risk perception” OR “attitude to risk*”) OR MM (“Decision Making” OR “Decision Support Techniques” OR Thinking OR “Risk Assessment” OR “Attitude to Risk” OR “Decision Making, Computer Assisted” OR “Decision Trees” OR “Decision Making, Patient” OR “Decision Making, Shared”)

AND

TI (“Secondary Prevention” OR prevention OR screening OR “early diagnosis” OR “early detection*” OR “Mass Screening” OR “Early Detection of Cancer”) OR AB (“Secondary Prevention” OR prevention OR screening OR “early diagnosis” OR “early detection*” OR “Mass Screening” OR “Early Detection of Cancer”) OR MM (“Health screening” OR “Cancer screening”)

AND

TI (“breast cancer” OR “breast cancers” OR “breast tumor” OR “breast tumors” OR “breast tumour” OR “breast tumours” OR “breast carcinoma*” OR “mammary cancer” OR “mammary cancers” OR “breast

neoplasm*” OR “mammary neoplasm*” OR “mammary carcinoma*” OR “mammary tumor” OR “mammary tumour” OR “mammary tumors” OR “mammary tumours” OR breast*) OR AB (“breast cancer” OR “breast cancers” OR “breast tumor” OR “breast tumors” OR “breast tumour” OR “breast tumours” OR “breast carcinoma*” OR “mammary cancer” OR “mammary cancers” OR “breast neoplasm*” OR “mammary neoplasm*” OR “mammary carcinoma*” OR “mammary tumor” OR “mammary tumour” OR “mammary tumors” OR “mammary tumours” OR breast*) OR MM (“Breast Neoplasms”)
OR
TI (“Ultrasonography, Mammary” OR mammo*) OR AB (“Ultrasonography, Mammary” OR mammo*) OR MM (Mammography)

Web of science editions science citations/social science/art/emerging (NO proceeding NO book)

TI=(“breast cancer” OR “breast cancers” OR “breast tumor” OR “breast tumors” OR “breast tumour” OR “breast tumours” OR “breast carcinoma*” OR “mammary cancer” OR “mammary cancers” OR “breast neoplasm*” OR “mammary neoplasm*” OR “mammary carcinoma*” OR “mammary tumor” OR “mammary tumour” OR “mammary tumors” OR “mammary tumours” OR breast* OR “Breast Neoplasms”) OR AB=(“breast cancer” OR “breast cancers” OR “breast tumor” OR “breast tumors” OR “breast tumour” OR “breast tumours” OR “breast carcinoma*” OR “mammary cancer” OR “mammary cancers” OR “breast neoplasm*” OR “mammary neoplasm*” OR “mammary carcinoma*” OR “mammary tumor” OR “mammary tumour” OR “mammary tumors” OR “mammary tumours” OR breast* OR “Breast Neoplasms”) OR AK=(“breast cancer” OR “breast cancers” OR “breast tumor” OR “breast tumors” OR “breast tumour” OR “breast tumours” OR “breast carcinoma*” OR “mammary cancer” OR “mammary cancers” OR “breast neoplasm*” OR “mammary neoplasm*” OR “mammary carcinoma*” OR “mammary tumor” OR “mammary tumour” OR “mammary tumors” OR “mammary tumours” OR breast* OR “Breast Neoplasms”)

AND

TI=(“Secondary Prevention” OR prevention OR screening OR “early diagnosis” OR “earlydetection*” OR “Early Detection of Cancer” OR “Screening Test*”) OR AK=(“Secondary Prevention” OR prevention OR screening OR “early diagnosis” OR “early detection*” OR “Early Detection of Cancer” OR “Screening Test*”) OR AB=(“Secondary Prevention” OR prevention OR screening OR “early diagnosis” OR “early detection*” OR “Early Detection of Cancer” OR “Screening Test*”)

__OR TI=(Mammography OR “Ultrasonography, Mammary” OR mammo*) OR AB=(Mammography OR “Ultrasonography, Mammary” OR mammo*) OR AK=(Mammography OR “Ultrasonography, Mammary” OR mammo*)

AND

TI=(decid* OR decision* OR choose OR chosen OR choosing OR choice* OR guide OR Preferen* OR tailor* OR personal* OR option* OR assist* OR aid* OR help*OR “computer-assisted” OR “risks assessment” OR “risks appraisal” OR “risk assessment” OR “risk appraisal” OR “risk evaluat*” OR “risk 6ide rag*” OR uncertainty OR “Decision Making” OR “Decision Theory” OR “Decision Support” OR “Shared Decision Making” OR Thinking OR “Risk perception”) OR AB=(decid* OR decision* OR choose OR chosen OR choosing OR choice* OR guide OR Preferen* OR tailor* OR personal* OR option* OR assist* OR aid* OR

help*OR “computer-assisted” OR “risks assessment” OR “risks appraisal” OR “risk assessment” OR “risk appraisal” OR “risk evaluat*” OR “risk 7ide rag*” OR uncertainty OR “Decision Making” OR “Decision Theory” OR “Decision Support” OR “Shared Decision Making” OR Thinking OR “Risk perception”) OR AK=(decid* OR decision* OR choose OR chosen OR choosing OR choice* OR guide OR Preferen* OR tailor* OR personal* OR option* OR assist* OR aid* OR help*OR “computer-assisted” OR “risks assessment” OR “risks appraisal” OR “risk assessment” OR “risk appraisal” OR “risk evaluat*” OR “risk 7ide rag*” OR uncertainty OR “Decision Making” OR “Decision Theory” OR “Decision Support” OR “Shared Decision Making” OR Thinking OR “Risk perception”)

AND

TI=(online OR “social media*” OR forum* OR platform* OR chat OR chats OR chatbot* OR “social network*” OR “information technolog*” OR crowdsourcing OR web OR webinar* OR website* OR digital OR mobile app* OR e-health OR ehealth OR Podcast* OR messaging OR eHealth OR Skype OR Facetime OR Twitter OR facebook OR virtual OR virtuali* OR e-tool OR eTool OR “web-based” OR “information display*” OR “computing method*” OR blog* OR “data display” OR “mobile health” OR m-health OR “digital health” OR Internet OR computer* OR interactive OR tablet OR “Patient Portal*” OR “Blogging” OR Internet OR “Social Networking” OR “Information Technology” OR “Data Display” OR Telemedicine OR DVD) OR AB=(online OR “social media*” OR forum* OR platform* OR chat OR chats OR chatbot* OR “social network*” OR “information technolog*” OR crowdsourcing OR web OR webinar* OR website* OR digital OR mobile app* OR e-health OR ehealth OR Podcast* OR messaging OR eHealth OR Skype OR Facetime OR Twitter OR facebook OR virtual OR virtuali* OR e-tool OR eTool OR “web-based” OR “information display*” OR “computing method*” OR blog* OR “data display” OR “mobile health” OR m-health OR “digital health” OR Internet OR computer* OR interactive OR tablet OR “Patient Portal*” OR “Blogging” OR Internet OR “Social Networking” OR “Information Technology” OR “Data Display” OR Telemedicine OR DVD) OR AK=(online OR “social media*” OR forum* OR platform* OR chat OR chats OR chatbot* OR “social network*” OR “information technolog*” OR crowdsourcing OR web OR webinar* OR website* OR digital OR mobile app* OR e-health OR ehealth OR Podcast* OR messaging OR eHealth OR Skype OR Facetime OR Twitter OR facebook OR virtual OR virtuali* OR e-tool OR eTool OR “web-based” OR “information display*” OR “computing method*” OR blog* OR “data display” OR “mobile health” OR m-health OR “digital health” OR Internet OR computer* OR interactive OR tablet OR “Patient Portal*” OR “Blogging” OR Internet OR “Social Networking” OR “Information Technology” OR “Data Display” OR Telemedicine OR DVD)

Psychinfo

MJMAINSUBJECT.EXACT(“Breast Neoplasms”) OR ab(“breast cancer” OR “breast cancers” OR “breast tumor” OR “breast tumors” OR “breast tumour” OR “breast tumours” OR “breast carcinoma*” OR “mammary cancer” OR “mammary cancers” OR “breast neoplasm*” OR “mammary neoplasm*” OR “mammary carcinoma*” OR “mammary tumor” OR “mammary tumour” OR “mammary tumors” OR “mammary tumours” OR breast*) OR ti(“breast cancer” OR “breast cancers” OR “breast tumor” OR “breast tumors” OR “breast tumour” OR “breast tumours” OR “breast carcinoma*” OR “mammary cancer” OR “mammary cancers” OR “breast neoplasm*” OR “mammary neoplasm*” OR “mammary carcinoma*” OR “mammary tumor” OR “mammary tumour” OR “mammary tumors” OR “mammary tumours”)

AND

ab(“Secondary Prevention” OR prevention OR screening OR “early diagnosis” OR “early detection*” OR “Mass Screening” OR “Early Detection of Cancer”) OR MJMAINSUBJECT.EXACT(“Cancer Screening”) OR MJMAINSUBJECT.EXACT(“Screening”) OR MJMAINSUBJECT.EXACT(“Screening Tests”) OR ti(“Secondary Prevention” OR prevention OR screening OR “early diagnosis” OR “early detection*” OR “Mass Screening” OR “Early Detection of Cancer”)

OR ab(mammography) OR ti(mammography) OR MJMAINSUBJECT.EXACT(“Mammography”)

AND

MJMAINSUBJECT.EXACT(“Risk Perception”)OR MAINSUBJECT.EXACT(“Decision Support Systems”) OR MJMAINSUBJECT.EXACT(“Decision Making”) OR MAINSUBJECT.EXACT(“Risk Assessment”) OR MAINSUBJECT.EXACT(“Critical Thinking”)) OR ti(decid* OR decision* OR choose OR chosen OR choosing OR choice* OR guide OR Preferen* OR tailor* OR personal* OR option* OR assist* OR aid* OR help*OR “computer-assisted” OR “risks assessment” OR “risks appraisal” OR “risk assessment” OR “risk appraisal” OR “risk evaluat*” OR “risk estimat*” OR uncertainty OR “Decision Support Techniques” OR “Decision Making” OR “Decision Theory” OR “Decision Support Systems” OR “Shared Decision Making” OR Thinking OR “Risk perception) OR ab(decid* OR decision* OR choose OR chosen OR choosing OR choice* OR guide OR Preferen* OR tailor* OR personal* OR option* OR assist* OR aid* OR help*OR “computer-assisted” OR “risks assessment” OR “risks appraisal” OR “risk assessment” OR “risk appraisal” OR “risk evaluat*” OR “risk estimat*” OR uncertainty OR “Decision Support Techniques” OR “Decision Making” OR “Decision Theory” OR “Decision Support Systems” OR “Shared Decision Making” OR Thinking OR “Risk perception)

AND

MJMAINSUBJECT.EXACT(“Social Media”) OR MAINSUBJECT.EXACT(“Internet”) OR MAINSUBJECT.EXACT(“Social Interaction”) OR MAINSUBJECT.EXACT(“Online Community”) OR MAINSUBJECT.EXACT(“Blog”) OR MAINSUBJECT.EXACT(“Websites”) OR MAINSUBJECT.EXACT(“Computer Mediated Communication”) OR MJMAINSUBJECT.EXACT(“Online Social Networks”) OR MJMAINSUBJECT.EXACT(“Social Networks”) OR MJMAINSUBJECT.EXACT(“Mobile Health”) OR MAINSUBJECT.EXACT(“Wearable Devices”) OR MAINSUBJECT.EXACT(“Mobile Technology”) OR MAINSUBJECT.EXACT(“Mobile Applications”) OR MAINSUBJECT.EXACT(“Mobile Devices”) OR MJMAINSUBJECT.EXACT(“Digital Interventions”) OR MAINSUBJECT.EXACT(“Social Media”) OR MAINSUBJECT.EXACT(“Virtual Classrooms”) OR MAINSUBJECT.EXACT(“Online Community”) OR MAINSUBJECT.EXACT(“Messages”) OR MAINSUBJECT.EXACT(“Internet Usage”) OR MAINSUBJECT.EXACT(“Online Social Networks”) OR MAINSUBJECT.EXACT(“Computer Applications”) OR MAINSUBJECT.EXACT(“Distance Education”) OR MJMAINSUBJECT.EXACT(“Computer Mediated Communication”) OR MAINSUBJECT.EXACT(“Text Messaging”) OR MAINSUBJECT.EXACT(“Mobile Devices”) OR ti(online OR “social media*” OR forum* OR platform* OR chat OR chats OR chatbot* OR “social network*” OR “information technolog*” OR crowdsourcing OR web OR webinar* OR website* OR digital OR mobile app* OR e-health OR ehealth OR Podcast* OR messaging OR eHealth OR Skype OR Facetime OR Twitter OR facebook OR virtual OR virtuali* OR e-tool OR eTool OR “web-based” OR “information display*” OR

“computing method*” OR blog* OR “data display” OR “mobile health” OR m-health OR “digital health” OR Internet OR computer* OR interactive OR tablet OR “Patient Portals” OR “Blogging” OR Internet OR “Social Networking” OR “Information Technology” OR “Data Display” OR “Computing Methodologies” OR “Online Systems” OR Telemedicine OR DVD) OR ab(online OR “social media*” OR forum* OR platform* OR chat OR chats OR chatbot* OR “social network*” OR “information technolog*” OR crowdsourcing OR web OR webinar* OR website* OR digital OR mobile app* OR e-health OR ehealth OR Podcast* OR messaging OR eHealth OR Skype OR Facetime OR Twitter OR facebook OR virtual OR virtuali* OR e-tool OR eTool OR “web-based” OR “information display*” OR “computing method*” OR blog* OR “data display” OR “mobile health” OR m-health OR “digital health” OR Internet OR computer* OR interactive OR tablet OR “Patient Portals” OR “Blogging” OR Internet OR “Social Networking” OR “Information Technology” OR “Data Display” OR “Computing Methodologies” OR “Online Systems” OR Telemedicine OR DVD)

Appendix S2: Data extraction

Multiple reports of the same study were collated [111]. Studies reporting about identical e-tool were grouped together and the main source of data (ie, used in our review and meta-analysis) was highlighted [111,112]; for study report(s) other than the main ones, type of sub-analysis and/or nature of additional outcomes reported by authors are indicated, pilot studies being also identified. In Table S2, details about studies and intervention designs are provided.

A data extraction form was used to collect the following information:

A. *General description of the study*

- Authors and year
- Country
- Population (number + age + gender)
- Objectives of the study (primary)
- Study design
- Study method (s)
- Study timeframe
- Study funders
- Comparison
- Previous stage of development

B. *The template for intervention description and replication checklist (TIDIeR) checklist (https://www.equator-network.org/wp-content/uploads/2014/03/TIDieR-Checklist-PDF.pdf)*

- Why: Brief name Rationale: Framework/theory used Aim of the tool
- What?
    - Decision aid (DA) or shared decision making (SDM), where clearly reported
    - Online interactive component(s)
    - Present harms and benefits
    - Risk estimates
    - Choice awareness
    - Option clarification
    - Patient preferences deliberation
    - Help making the decision
    - Others (to complete)
- Who provided?
- Where?
- When and how much?
- Tailoring
- Modifications

C. *Intervention assessment*

- Outcomes to be measured
- Measures/scales used to measure outcomes
- Type of outcomes: Patient outcomes, Physician outcomes, System outcomes

D. *Knowledge and Cognitive determinants*

- Knowledge /tailored information received, feeling informed about:

- Breast cancer (BC) and breast cancer screening (BCS) including harms and benefits, statistics, risk factors, risk estimates
- Options, features
- Awareness (ie, knowing that a decision needed to be made)

- Cognitive determinants
    - Risk perceptions
    - Clarity about value and preference
    - Informed choice as defined by the authors
    - Self-efficacy, perceived barriers or other perceived behavioural control items
    - Attitudes and beliefs towards BC and BCS
    - others

E. *Process of the decision making and feelings related to the decision*

- Values-choice congruence
- Decisional conflict
- Satisfaction with the decision
- Decision regret
- Other feelings associated with the decision: (anxiety, depression, emotional distress, confidence…) Patient-clinician communication
- Participation in decision making
- Stage of the decision (e.g. precontemplation, contemplation… Prochaska's model)
- Others (in case a study used different constructs such as personal empowerment, social competency, etc.)

F. *Screening intention and behaviour*

- Intention to be screened (yes, no, not sure)
- Behaviour regarding whether or not to be screened (including real data, screening coverage, screening uptake, self-reported data) – Including any data by subgroups-any data on long term_
- Adherence with the decision to be screened or not (on the long term)
- Undecided regarding screening
- Other behavioural outcomes (ie, to seek more information about screening, or seek advice from someone else besides the physician), that are not involving the physician, which are reported elsewhere regarding communication and participation

G. *Implementation*

- Resource use: e.g. Costs or cost-effectiveness, resource requirements, Impact (on services, public health etc), costs of e-tools including the impact on health care system indicators (consultation time)
- Usability/Acceptability: participants (uptake/engagement/disengagement in SDM use), health professionals (e.g. consultation length, Litigation rates, distress of physicians, confidence of physicians, satisfaction of physicians)
- Feasibility (for participants and/or health professionals)
- Equity and rights: e.g. uptake/engagement in SDM use by subgroups (deprivation, ethnicity, age, etc)
- Fidelity and Sustainability (alterations have been made to the original tool or intervention in order to be adopted first and then become sustainable)
- Sustainability costs (to maintain the tool after its implementation)

Appendix S3: Supplementary information regarding meta-analyses.

PART A. General rules

Data were collected from the studies reports and we contacted directly the authors where additional data or clarifications were required. Meta-analyses (Intention to treat analysis) were performed and were limited to the RCTs included in the review, following Cochrane guidance [47]. We applied the following rules:

1. When an RCT was used to assess different e-tools through different arms, we restricted the extraction and analysis to the arm of interest, excluding mixed intervention [47, 112]. For example, in Champion et al's RCTs [56,57], we extracted and analysed data related to the "DVD" or "web" arms. In Seitz et al's study [71], in which several control arms were available, we chose to report the control which was the most similar to "usual care" (See Table S2).

2. For each outcome of interest (See Table 2 in the full JMIR manuscript), the decision to combine the results of the selected RCT studies reporting the outcome, in a meta-analysis, was based on the following criteria:

    i. When one outcome was available both as a dichotomous or a continuous variable, we chose to report the type of variable that would allow the combination of a larger amount of studies;

    ii. To minimize source of heterogeneity between studies, RCTs reported in the meta-analyses had to report both a similar definition of the outcome and a similar instrument used to measure the outcomes. This approach is different to those applied in Yu et al's study to report on the effect of web-based tools [38]. Therefore, we reported decisional conflict outcome with RCTs using the "Sure" scale only, and we did not report on the pooled effect regarding "the regret" outcome (See Table 2 in the full JMIR manuscript);

    iii. Two original study reported results stratified by groups based either on individual risk (Seitz et al, [71]) or income (Champion et al [57]). In meta-analyses and for each of those studies, original stratified data were combined [47], to both increase sample sizes and report on a population more similar to a population to be screened (ie, invited to participate by BCS programmes). Regarding Seitz et al's e-tool [71], combining risk groups was possible due to the fact that women invited to use the tool were not at higher risk of breast cancer based on eligibility criteria (ie, at no previous history of breast cancer or presence of BRCA1 or BRCA2 mutation); intention was then defined as intention to continue or start screening in their forties or to start or have the next screening at 50 (Seitz et al [71]).

3. In Seitz et al's [71] and Lin and Wang's [68] studies, different e-tools presenting different degree of complexity of tailored messaged were tested through different arms. Where meta-analyses were performed with any of those studies' data (ie, to assess tools' effects on intention, worry or accuracy of risks) all the different e-tools were tested. We reported, in the results section of the full JMIR manuscript, only the meta-analyses conducted with the Seitz et al's "extended information with untailored exemplars" (Seitz et al [71]) and the Lin and Wang's "tailored message intervention" (TMI) e-tools data [68]; those tools were the most similar to the other tools included in the corresponding meta-analyses and drove lowest heterogeneity between results. Results obtained with the other Seitz et al's [71] and Lin and Wang's [68] e-tools are reported in Appendix S4 and Figures S6 and S7.

Some of the included studies involved both men and women populations, as the e-tools were providing information on other type of cancer screening and/or prevention (Results, Table 1 in the full JMIR manuscript) (Henry et al [59]; Klippert et Schaper [60]; Krist et al [61,67]; Walsh et al [64]). Regarding data related to

BCS, authors reported results for women only. For example, Krist et al [61, 67] reported data restricted women aged 50-75 and eligible to BCS.

PART B. Reasons to exclude some studies when reporting specific outcomes (Supplementary data to Table 2 in the full JMIR manuscript)

Behaviour, exclusion of Fissler et al's study [58]: the two items that were used to assess women decision after using the tool ("to consider participation in mammography screening to be very reasonable" and "I would participate in breast cancer screening using mammography") were more related to "attitudes" and we did not reported them as behaviour outcome

Attitudes about BCS, exclusion of Lin and Wang [68] /Lin and Effken [76] and Lee et al's studies [54, 81]: these studies assessed women's perception toward BCS using the Decisional Balance for Mammography Inventory instrument that measured not only attitudes but also knowledge, personal experience, others' experience and feelings. In Lee et al's study [54], attitudes were measured through fatalism, modesty, social support and fear of discovery.

Risk perceptions, exclusion of Eden et al's study [72, 82]: in a follow-up analysis of the parent study (Eden et al [72]), authors evaluated perception of breast cancer risk associated with abnormal mammograms of a woman in a similar age (ie, 40) (Klein et al [82]).

Appendix S4: Intention, exploration of effects of variables and other Lin and Wang's [68] and Seitz et al's [71] e-tools.

We explored heterogeneity through different variables, e.g. when intention was measured in relation to either to age or time (see Table 2 in the full JMIR manuscript): no decrease of heterogeneity was observed (not shown). We also explored the effect of using different Lin and Wang's [68] and Seitz et al's [71] e-tools (below), and effect of subgrouping based on the nature of the e-tools: tailored vs features-with-tailoring e-tools (Figure S3).

PART A. Intention when using Li and Wang's "tailored message intervention" (TMI) e-tool [68] and with different Seitz et al.'s e-tool [71] as listed below: low or intermediate heterogeneity between studies and no statistical differences.

Brief information plus expository (no exemplars):
Heterogeneity: $Chi^2 = 6.81$, df = 5 (P = 0.23); $I^2 = 27\%$
Test for overall effect: Z = 1.70 (P = 0.09)

Brief information plus untailored exemplars:
Heterogeneity: $Chi^2 = 7.25$, df = 5 (P = 0.20); $I^2 = 31\%$
Test for overall effect: Z = 1.17 (P = 0.24)

Brief information plus tailored exemplars:
Heterogeneity: $Chi^2 = 8.74$, df = 5 (P = 0.12); $I^2 = 43\%$
Test for overall effect: Z = 0.83 (P = 0.41)

Extended information plus expository (no exemplars):
Heterogeneity: $Chi^2 = 7.20$, df = 5 (P = 0.21); $I^2 = 31\%$
Test for overall effect: Z = 1.89 (P = 0.06)

Extended information plus tailored exemplars:
Heterogeneity: $Chi^2 = 8.48$, df = 5 (P = 0.13); $I^2 = 41\%$
Test for overall effect: Z = 0.88 (P = 0.38)

PART B. Intention when using Li and Wang's "complete tailored intervention" (CTI) e-tool [68] and with different Seitz et al's e-tool [71] as listed below: high heterogeneity (I2 ≥67%) between studies and no statistical differences.

Brief information plus expository (no exemplars):
Heterogeneity: $Chi^2 = 15.51$, df = 5 (P = 0.008); $I^2 = 68\%$
Test for overall effect: Z = 2.15 (P = 0.03)

Brief information plus untailored exemplars:
Heterogeneity: $Chi^2 = 15.59$, df = 5 (P = 0.008); $I^2 = 68\%$
Test for overall effect: Z = 1.62 (P = 0.11)

Brief information plus tailored exemplars:
Heterogeneity: $Chi^2 = 16.80$, df = 5 (P = 0.005); $I^2 = 70\%$
Test for overall effect: Z = 1.28 (P = 0.20)

Extended information plus expository (no exemplars)
Heterogeneity: $Chi^2 = 16.00$, df = 5 (P = 0.007); $I^2 = 69\%$
Test for overall effect: Z = 2.34 (P = 0.02)

Extended information plus untailored exemplars:
Heterogeneity: $Chi^2 = 15.27$, df = 5 (P = 0.009); $I^2 = 67\%$

Test for overall effect: $Z = 1.95$ ($P = 0.05$)

Extended information plus tailored exemplars:
Heterogeneity: $Chi^2 = 16.58$, $df = 5$ ($P = 0.005$);
$I^2 = 70\%$ Test for overall effect: $Z = 1.32$ ($P = 0.19$)

Table S1. PRISMA 2020 checklist.

Our systematic reviews and meta-analyses adhere to the PRISMA Statement [42]

Section and Topic	Item #	Checklist item	Location where item is reported *(note: page may have changed during copyediting)*
**TITLE**			
Title	1	Identify the report as a systematic review.	**Page 1**
**ABSTRACT**			
Abstract	2	See the PRISMA 2020 for Abstracts checklist.	Pages 3-4: We used PRISMA for abstracts (see below)
**INTRODUCTION**			
Rationale	3	Describe the rationale for the review in the context of existing knowledge.	Pages 4-6
Objectives	4	Provide an explicit statement of the objective(s) or question(s) the review addresses.	Page 6: Through a combined systematic review and meta-analysis approach, our research aimed to address these evidence gaps and synthesise scientific literature regarding e-tools in the context of BCS by mammography
**METHODS**			
Eligibility criteria	5	Specify the inclusion and exclusion criteria for the review and how studies were grouped for the syntheses.	Page 7
Information sources	6	Specify all databases, registers, websites, organisations, reference lists and other sources searched or consulted to identify studies. Specify the date when each source was last searched or consulted.	Page 7: We systematically searched Medline (via PubMed), PsycINFO, Embase, CINAHL and Web of Science databases from August 2010 to August 2020 with was updated in April 2023 (Appendix S1).
Search strategy	7	Present the full search strategies for all databases, registers and websites, including any filters and limits used.	Appendix S1
Selection process	8	Specify the methods used to decide whether a study met the inclusion criteria of the review, including how many reviewers screened each record and each report retrieved, whether they worked independently, and if applicable, details of automation tools used in the process.	Page 7: Three independent (PV, ALB, CB) reviewers assessed titles and abstracts and then full texts against study inclusion criteria.
Data collection process	9	Specify the methods used to collect data from reports, including how many reviewers collected data from each report, whether they worked independently, any processes for obtaining or confirming data from study investigators, and if applicable, details of automation tools used in the process.	Page 7: A data extraction form …..<br>Page 9, line 222: Data were extracted from the original publication and, where necessary, additional information was obtained by directly contacting the authors.
Data items	10a	List and define all outcomes for which data were sought. Specify whether all results that were compatible with each outcome domain in each study were sought (e.g. for all measures, time points, analyses),	Page 7 and page 8

Section and Topic	Item #	Checklist item	Location where item is reported *(note: page may have changed during copyediting)*
		and if not, the methods used to decide which results to collect.	
	10b	List and define all other variables for which data were sought (e.g. participant and intervention characteristics, funding sources). Describe any assumptions made about any missing or unclear information.	Page 8: A data extraction form was used …..
Study risk of bias assessment	11	Specify the methods used to assess risk of bias in the included studies, including details of the tool(s) used, how many reviewers assessed each study and whether they worked independently, and if applicable, details of automation tools used in the process.	Page 9, Line 225: PV and CB, ALB or LD, performed risk-of-bias assessment of all the RCTs ….
Effect measures	12	Specify for each outcome the effect measure(s) (e.g. risk ratio, mean difference) used in the synthesis or presentation of results.	Page 10, Line 238: We reported pooled estimates as 'difference in means (MD)' ….
Synthesis methods	13a	Describe the processes used to decide which studies were eligible for each synthesis (e.g. tabulating the study intervention characteristics and comparing against the planned groups for each synthesis (item #5)).	Page 9, Line 231: To minimize source of heterogeneity between studies, and for each outcome, ….
	13b	Describe any methods required to prepare the data for presentation or synthesis, such as handling of missing summary statistics, or data conversions.	Page 9, Line 236: We used the Review Manager software (Revman) ….
	13c	Describe any methods used to tabulate or visually display results of individual studies and syntheses.	Page 9, Line 236: We used the Review Manager software (Revman) ….
	13d	Describe any methods used to synthesize results and provide a rationale for the choice(s). If meta-analysis was performed, describe the model(s), method(s) to identify the presence and extent of statistical heterogeneity, and software package(s) used.	Page 9, Line 236: We used the Review Manager software (Revman) …Heterogeneity or inconsistency between studies was evaluated using the I^2 statistic. Fixed effect (FE) model was applied when low heterogeneity was found ($I^2 \leq 30$); a random-effects (RE) model was used in the other cases. Results from the meta-analyses presenting intermediate or high heterogeneity between studies (ie, $I^2 \geq 35$), were further explored to strengthen the validity of our results.
	13e	Describe any methods used to explore possible causes of heterogeneity among study results (e.g. subgroup analysis, meta-regression).	Page 10, Line 241: To reduce heterogeneity, …..<br>Page 10, line 249. Two approaches were used to explore the causes of intermediate/high heterogeneity ……
	13f	Describe any sensitivity analyses conducted to assess robustness of the synthesized results.	Page 9, line 236: We used the Review Manager software (Revman)…
Reporting bias assessment	14	Describe any methods used to assess risk of bias due to missing results in a synthesis (arising from reporting biases).	Page 9, Line 225: PV and CB, ALB or LD, performed risk-of-bias assessment of all the RCTs …… Level of risk for each domain of RoB 2 (A: randomization process, B: deviations from the intended protocol; C: Missing data; D: outcome measurement; E: selection of reported result) or overall risk (F) was evaluated for each RCT and

Section and Topic	Item #	Checklist item	Location where item is reported ***(note: page may have changed during copyediting)***
			reported on the forest plots as low (green), moderate (yellow) of high (red).
Certainty assessment	15	Describe any methods used to assess certainty (or confidence) in the body of evidence for an outcome.	Page 9, Line 212: Finally, for each outcome, the overall certainty of the evidence collected through the meta-analyses and SwiM exercise was evaluated independently by PV and LD using the GRADE methodology
**RESULTS**			
Study selection	16a	Describe the results of the search and selection process, from the number of records identified in the search to the number of studies included in the review, ideally using a flow diagram.	Figure 1
	16b	Cite studies that might appear to meet the inclusion criteria, but which were excluded, and explain why they were excluded.	Figure 1
Study characteristics	17	Cite each included study and present its characteristics.	Table S1
Risk of bias in studies	18	Present assessments of risk of bias for each included study.	In Forest plots (Figures 2-3, Appendix S4 and Figures S1-S8)
Results of individual studies	19	For all outcomes, present, for each study: (a) summary statistics for each group (where appropriate) and (b) an effect estimate and its precision (e.g. confidence/credible interval), ideally using structured tables or plots.	Described in result section and in Figures 2 and 3, Appendix S4 and Figures S1-S8
Results of syntheses	20a	For each synthesis, briefly summarise the characteristics and risk of bias among contributing studies.	Figures 2 and 3, Appendix S4 and Figures S1-S8
	20b	Present results of all statistical syntheses conducted. If meta-analysis was done, present for each the summary estimate and its precision (e.g. confidence/credible interval) and measures of statistical heterogeneity. If comparing groups, describe the direction of the effect.	Described in result section Pages 18-24, Figures 2 and 3, Appendix S4 and Figures S1-S8
	20c	Present results of all investigations of possible causes of heterogeneity among study results.	Described in result section Pages 18-24, Figures 2 and 3, Appendix S4 and Figures S1-S8
	20d	Present results of all sensitivity analyses conducted to assess the robustness of the synthesized results.	Figures 2 and 3, Appendix S4 and Figures S1-S8: forest plots with Chi2 and test for overall effect
Reporting biases	21	Present assessments of risk of bias due to missing results (arising from reporting biases) for each synthesis assessed.	Level of risk for RoB 2 domain C (Missing data) is reported in Figures 2 and 3 and in Figures S1-S8
Certainty of evidence	22	Present assessments of certainty (or confidence) in the body of evidence for each outcome assessed.	Pages 18-24, Figures 2 and 3, Appendix S4 and Figures S1-S8: results were presented 95% CI and p value. In forest plots: Chi2 and test for overall effect.<br>Page 25, Grading of the available evidence….
**DISCUSSION**			

Section and Topic	Item #	Checklist item	Location where item is reported *(note: page may have changed during copyediting)*
Discussion	23a	Provide a general interpretation of the results in the context of other evidence.	Page 28, Line 549: This is the first systematic review providing a comprehensive overview …..
	23b	Discuss any limitations of the evidence included in the review.	Pages 31-32: 'Limitations' paragraph
	23c	Discuss any limitations of the review processes used.	Page 31, Line 651: In addition, we (i) did not carefully review the content of the BCS information provided in the e-tools …..
	23d	Discuss implications of the results for practice, policy, and future research.	Page 30: Recommendations for future developments
**OTHER INFORMATION**			
Registration and protocol	24a	Provide registration information for the review, including register name and registration number, or state that the review was not registered.	Page 4: PROSPERO, CRD42020164479
	24b	Indicate where the review protocol can be accessed, or state that a protocol was not prepared.	PROSPERO website
	24c	Describe and explain any amendments to information provided at registration or in the protocol.	Page 6, Line 153: The main deviation was …
Support	25	Describe sources of financial or non-financial support for the review, and the role of the funders or sponsors in the review.	Page 33: Funding information
Competing interests	26	Declare any competing interests of review authors.	Page 33: The authors declare no conflicts of interest.
Availability of data, code and other materials	27	Report which of the following are publicly available and where they can be found: template data collection forms; data extracted from included studies; data used for all analyses; analytic code; any other materials used in the review.	Page 33, Data availability

PRISMA 2020 for Abstracts Checklist

Section and Topic	Item #	Checklist item	Reported (Yes/No)
**TITLE**			
Title	1	Identify the report as a systematic review.	Yes
**BACKGROUND**			

Section and Topic	Item #	Checklist item	Reported (Yes/No)
Objectives	2	Provide an explicit statement of the main objective(s) or question(s) the review addresses.	Yes
**METHODS**			
Eligibility criteria	3	Specify the inclusion and exclusion criteria for the review.	Yes
Information sources	4	Specify the information sources (e.g. databases, registers) used to identify studies and the date when each was last searched.	Yes
Risk of bias	5	Specify the methods used to assess risk of bias in the included studies.	Yes
Synthesis of results	6	Specify the methods used to present and synthesise results.	Yes
**RESULTS**			
Included studies	7	Give the total number of included studies and participants and summarise relevant characteristics of studies.	Yes
Synthesis of results	8	Present results for main outcomes, preferably indicating the number of included studies and participants for each. If meta-analysis was done, report the summary estimate and confidence/credible interval. If comparing groups, indicate the direction of the effect (i.e. which group is favoured).	Yes
**DISCUSSION**			
Limitations of evidence	9	Provide a brief summary of the limitations of the evidence included in the review (e.g. study risk of bias, inconsistency and imprecision).	Yes
Interpretation	10	Provide a general interpretation of the results and important implications.	Yes
**OTHER**			
Funding	11	Specify the primary source of funding for the review.	No, as a specific paragraph is provided
Registration	12	Provide the register name and registration number.	Yes

Table S2. Characteristics of the included studies.

Authors, year[*]	Country, county	Study design and complexity of the intervention †	Intervention §<br><br>Comparison (s)§	Health care services involvement¶	Aim
**Bowen et al, 2011 [65]**	USA, Seattle metropolitan area	RCT<br>Complex: reminders to participants who did not log on; monthly newsletter by email or mail to intervention and control groups; phone call to high-risk group to enrol for counselling	WebApp<br><br>Sole usual care	Possible implementation	To increase screening behaviours, both mammography and breast self-examination
**Champion et al, 2016 [57]****<br><br>Gathirua-Mwangi et al, 2016 [79]<br>Sub-analysis on African American women<br>Skinner et al, 2011 [83]<br>Follow-up analysis about usability, exposure	USA, Indiana University and North Carolina	RCT<br>Simple	Interactive DVD§<br><br>Usual care: depending on the healthcare provider, it ranged from nothing to a post-card reminder when it was time to schedule an appointment	Recruitment	To increase mammography adherence
**Champion et al, 2020 [56]††**	USA, Louisiana.	RCT<br>Simple intervention (Reminder to complete the intervention)	WebApp§<br><br>Usual care: depending on the healthcare provider, it ranged from nothing to a post-card reminder when it was time to schedule an appointment	Recruitment	To provide tailored messages for both breast cancer and colorectal cancer screening
**Champion et al, 2022 [66] § §**	USA, rural counties from Indiana and Ohio	RCT<br>Simple (health-related newsletters at baseline and six months to both intervention and control groups)	Interactive DVD<br><br>Sole usual care	No	To increase breast, cervical and colorectal cancer screening among rural women

**Authors, year**[*]	**Country, county**	**Study design and complexity of the intervention** †	**Intervention** § <br>**Comparison (s)**§	**Health care services involvement**¶	**Aim**
**Eden et al, 2015 [72]**	USA, Oregon	Pre-post study<br>Complex	Optimised for mobile WebApp<br><br>Pre-intervention (“usual care”)	Recruitment and implementation	To help users gain deeper insights into their priorities for screening and prepare them to discuss mammography screening with their health care providers
Klein et al, 2016 [82]<br>Follow-up analysis; additional outcomes: tool users’ comprehension, memory, and impressions of risk communication messages	USA, Oregon	Follow-up study<br>(Mixed method)			
**Eden et al, 2020 [73]**	USA, Oregon	Prospective single arm<br>Simple (complex IT system that handling reminders)	Patient portal<br><br>No comparison	Recruitment and implementation (patient portal)	To help women understand their personal risks for breast cancer, priorities for screening, and screening options before discussing them with their physicians
**Elkin et al, 2017 [75]**	USA, New York,	Prospective single arm<br>Complex due to invitations<br>(Mailed and emailed 4–6 weeks before the scheduled visit, included a personalized letter signed by the woman’s physician with instructions for accessing the tool and a unique username and password. On site, access to the tool at each participating clinic was offered to women who did not have	WebApp<br><br>NA	Recruitment and partial implementation	To help women make decisions about screening mammography that are informed and consistent with their values and preferences, and to serve as a foundation for discussions about screening mammography between women and their health care providers

Authors, year*	Country, county	Study design and complexity of the intervention †	Intervention §<br><br>Comparison (s)§	Health care services involvement¶	Aim
		internet access elsewhere)			
**Fissler et al, 2015 [58]**	Germany, Tuebingen	RCT 2x2 factorial design<br>Simple	WebApp with AI-based virtual doctor; different e-tools designs based on using two doctor's communication style (oriented to patients' needs or not oriented (ie, fact-oriented) and two patients needs conditions (ie, patients' needs were made salient or not)<br><br>WebApp: IA-doctor with a non-oriented communication style and patients' needs were not made salient	No	To evaluate how advice seekers' salient needs and doctor's communication styles influenced advice seekers' attitudes toward mammography screening and their decision whether or not to participate in this procedure
**Henry et al, 2016 [59]**	USA, Pasadena,	Interventional study without concurrent control<br>Complex	Patient portal<br><br>Non-users	Recruitment and implementation (patient portal)	To better enable users to take charge of their care in order to improve the efficiency and efficacy of outreach efforts and minimize the need for panel management
**Klippert et Schaper, 2018 [60]**	USA, Idaho	Retrospective single arm<br>Complex: 6 posts	Social media-based intervention<br><br>NA	No (a public health department in Idaho posted the mammography campaign on its Facebook Page)	To promote breast cancer screening
**Krist et al, 2012 [61]**	USA, Virginia	RCT<br>Simple: up to 3 mailed invitations.	Patient portal<br><br>Usual care, with no mailings about the portal and unable to access the system	Recruitment and implementation (patient portal)	To better enable users to take charge of their care in order to improve the efficiency and efficacy of outreach efforts and minimize the need for panel management

Authors, year*	Country, county	Study design and complexity of the intervention †	Intervention §<br><br>Comparison (s)§	Health care services involvement¶	Aim
**Krist at al, 2017 [67]**	USA, Virginia	Interventional study without concurrent control<br>Complex by using complex method to invite participants; three invitation phases with 3 different invitation mechanisms, reminder emails	Patient portal<br><br>Non-users	Recruitment and implementation (patient portal)	To engage patients through an informed decision-making module regarding cancer screening (colon, breast, prostate)
**Lee et al, 2017[54]**<br><br>Lee et al, 2018 [81]<br>Follow-up analysis (Qualitative, focus groups). Additional outcomes obtained with tool's users: tool's perception, impact of the tool on perception, knowledge, attitude and motivation for screening, screening experience	USA, Minnesota	RCT (pilot)<br>Complex (assistance to upload app and navigation and intensity of the intervention)	Smartphone Mobile application with GPS<br><br>Usual care: printed brochure in the Korean language with contact information of health navigator for questions, community clinics, indicating those that offer low-cost or free mammography)	No	To promote mammograms among underserved immigrant women
**Lin and Wang, 2009 [68]¶¶**	Taiwan, Hualien	RCT<br>Simple	WebApp with complete tailored intervention (tailored messages plus tailored activities (CTI)) or tailored message intervention (tailored messages only, TI)	No	To increase Taiwanese women's perceptions of and intentions to obtain mammography

Authors, year[*]	Country, county	Study design and complexity of the intervention †	Intervention §<br><br>Comparison (s)§	Health care services involvement¶	Aim
			Website ('Standard Intervention'): online Decisional Balance for Mammography Inventory (DBMI) and access to a website with PDF of an educational brochure (developed by a Health Department and commonly distributed to the public)		
Lin and Effken, 2010 [76]<br>Same as above, reporting demographics data and reporting only partial results	Taiwan, Hualien				
Lin et al, 2011 [80]<br>pilot before RCT	Taiwan, Hualien				
**Mathieu et al, 2010 [69]**	Australia, Sydney	RCT<br>Simple	WebApp Intervention<br><br>Usual care: ie, no information provided as screening programme did not provide information to this age range	No (except for the trial advertisement)	To assist women to weigh up the outcomes and clarify their personal values and preferences before making a decision about breast cancer screening
**Pereira et al, 2020 [62]**	Brazil, Maringá	Pre-post study<br>Complex (difficulty of behaviours required by those delivering the intervention: social media group intensity and people involved (2 observers, one mediator responsible of sending messages and clarifications))	Social media-based<br><br>Pre-intervention ("usual care")	Recruitment	To enhance breast cancer knowledge in women
**Reder et Kolip, 2017 [70]**	Germany, Westphalia-Lippe	RCT	WebApp Intervention	Recruitment and implementation (ie, linked to the breast	To increase the proportion of women making informed

Authors, year[*]	Country, county	Study design and complexity of the intervention [†]	Intervention [§]<br><br>Comparison (s)[§]	Health care services involvement[¶]	Aim
		Simple: a reminder was e-mailed 10 days after each survey	Usual care: normal invitation letter plus brochure	cancer screening programme)	choices regarding mammography screening
Reder et al, 2019 [84]<br>Different analysis to assess role of ehealth literacy					
**Roberto et al, 2020 [55]**	Italy, different regions	RCT<br>Simple	WebApp Intervention<br><br>A web-based standard static brochure that was assembled on the basis of brochures used in the participant-organised screening programmes	Recruitment and implementation (ie, the intervention was embedded in breast cancer screening programmes)	To improve informed choice in organised breast cancer screening.
**Scariati et al, 2015 [74]**	USA, Oregon	Pre-post study (pilot)<br>Simple	WebApp<br><br>Pre-intervention ("usual care")	No	To empower women to make a screening decision that is right for them and to facilitate a shared decision-making process
**Schapira et al, 2019 [63]**	USA, Pennsylvani a	RCT<br>Complex including enrolment and training of health professionals	WebApp Intervention<br><br>Participants had to fill out a breast cancer risk assessment -not indicated whether online or not-then proceeded with usual care	Recruitment and partial implementation	To evaluate individual risk estimates for women 39 to 48 years of age regarding the decision of age of initiation of breast cancer screening
**Seitz et al, 2016 [71]*****	USA, Philadelphia	RCT<br>Simple	WebApp Intervention (2X3 design) tailored to the individual risk with two independent variables tested (ie, amount of information (brief vs extended) and arguments to follow the guidelines (with three possible formats: didactic, untailored exemplars, or exemplars tailored to the individual)).	No	To improve match between breast cancer risk and mammography intentions. Investigates how perceived susceptibility to breast cancer or emotion (worry or fear) predict mammography intentions

Authors, year[*]	Country, county	Study design and complexity of the intervention †	Intervention §<br><br>Comparison (s)§	Health care services involvement¶	Aim
Seitz et al, 2018 [78]<br>Follow-up analysis: different outcomes reported such as perceived susceptibility, worry and fear			Website with no info or basic info regarding mammography§		
**Walsh et al, 2020 [64]**<br><br>Arora et al, 2013 [77]<br>Feasibility-acceptability pilot study before RCT; knowledge	USA, San Francisco	RCT<br>Complex; involving presentations to clinicians at the participating clinics.	Mobile application on ipad Intervention<br><br>A video about healthy lifestyles	Recruitment and implementation	To increase rates of screening and screening discussions for all appropriate cancers

NA: non-applicable; BCS: breast cancer screening; CRC: colorectal cancer screening; HS: health system; AI: artificial intelligence; IT: information technology; RCT: randomized controlled trial

[*] Study reports are presented in alphabetical order (first author) and, where first authors are identical, in ascending year of publication. The scientific publications used in our review and meta-analysis are highlighted. Those reporting about identical e-tool were grouped together and the main source of data (ie, used in our review and meta-analyses) was highlighted [112]; for study report(s) other than the main ones, type of sub-analysis and/or nature of additional outcomes reported by authors are indicated, pilot studies being also identified.

† Design: either RCT or any other quantitative design (pre-post, single arm or interventional study without concurrent control). Nature of complexity of the implementation of intervention was specified; "complex" interventions were those requiring i) a number of interacting components within the experimental and/or control interventions or/and ii) a number and difficulty of behaviours required by those delivering or receiving the intervention [113]. We did not consider complexity of IT system. "Simple" means that the intervention was solely provided. Where specified, we provided additional information that may be of interest to the reader.

§ Details regarding intervention and comparison are provided; when an RCT design was used and different arms were available, we reported in our review only the information and data related to the intervention/arm of interest [47] (see also Appendix S3). If several control arms were available, and where possible, "usual care" was chosen as control group (See also Appendix S3). Where specified, we provided some additional information about "usual care", that could be of interest to the reader. Five tools tested through RCTs used as comparator either a control website/webApp (Fissler et al [58]; Lin and Wang [68]; Roberto et al [55], Seitz et al [71]) or a video about health (Walsh et al [64]).

¶Where specified, the available and usual healthcare services were involved either to recruit participant ("recruitment") and/or to implement the intervention ("implementation"). In some cases, we identified "partial implementation" (ie. access to the e-tool was offered at health care service to participants with no internet (Schapira et al [63]; Elkin et al [75])), or "possible implementation" (ie, opportunity to contact health professionals was given to participants (Bowen et al [65])).

** Only the information and data related to the "interactive DVD" arm were reported in our review. Population was pooled independent of income in meta-analysis (See also Appendix S3).

†† Parent trial is described in Champion et al's paper [114]. Only the information and data related to the "Web" arm were reported in our review (see Appendix S3).

§ § Reporting a subset analysis performed with the subpopulation of women who were either non adherent to BCS only or were non-adherent also to CRC or/and cervical cancer screening. In the parent trial, women were recruited to be non-adherent to breast and/or cervical and/or colorectal cancer screening [115]
¶¶ We used the arm that was more resembling to other tools reported in the meta-analysis (ie, tailored message intervention (TMI)). Results obtained with the complex tailored intervention (CTI) are reported in Appendix S4.
*** Two comparison conditions were used: providing either no information or basic information (ie, current mammography guidelines, a statement that women between the ages of 40 and 50 years old have a choice to make about when to begin mammography, screening options). The basic information was used as comparison group in our meta-analysis, as it resembled the most to usual care. Intervention (2X3 design) tested different amount of complexity of the tailored messages (ie, trough amount of information (brief vs extended) and arguments to follow the guidelines (didactic, untailored exemplars or tailored exemplars)). We used the “extended amount of information with untailored exemplars” as the intervention group in our meta-analyses (see Appendix S3). Other data are reported in Appendix S4 and Figures S6 and S7.

Table S3. Characteristics of the study populations.

Characteristics*	
Type	➢ General population : Bowen et al [65]; Champion et al [66]; Fissler et al [58]; Klippert et Schaper [60]; Lee et al [54]; Lin and Wang [68]; Mathieu et al [69]; Scariati et al [74]; Seitz et al [71], and -linked to breast cancer screening programme- Reder et Kolip [70]; Roberto et al [55]<br>➢ Patients (ie, participants recruited from hospital of health centre database): Champion et al [56, 57]; Pereira et al [62]<br>➢ "Active patients": identified as "active" with no details (Eden et al [72]), with an office visit for any reason in the year before the start of recruitment (Krist et al, 2012), recruited at the hospital or clinic before a pre-scheduled visit (Elkin et al, 2017; Schapira et al [63]; Walsh et al [64]) or trough patient portals (Eden et al [73]; Henry et al [59]; Krist et al [61, 67])
Gender	➢ Men and women: Henry et al [59], Klippert et Schaper [60], Krist et al [61, 67]; Walsh et al [64]<br>➢ Women: all other studies
Age †	➢ Not specificied: Fissler et al [58]; Henry et al [59]<br>➢ Around 40 y.o.: Eden et al [72]; Elkin et al [75]; Klippert et Schaper [60]; Mathieu et al [69]; Scariati et al [74]; Schapira et al [63]; Seitz et al [71])<br>➢ Around 50 y.o.: Champion et al [56]; Krist et al [67]; Reder et Kolip [70]; Roberto et al [55]; Walsh et al [64]<br>➢ Wider age range: 40-74 y.o. (Eden et al [73]); 35-69 y.o. (Lin and Wang [68]); 45-69 y.o. (Pereira et al [62]); 35-70 y.o. (Bowen et al [65]); 41-65 y.o. (Champion et al, [57]); 50-74y.o (Champion et al [66]); 18-75 y.o. (Krist et al [61])
Women' breast cancer risk other than age	➢ Not assessed : Champion et al, [56,57]; Fissler et al [58]; Henry et al [59]; Klippert et Schaper [60]; Krist et al [61] ; Lee et al [54]; Pereira et al [62]; Schapira et al [63]; Walsh et al [64]<br>➢ Assumed to be average based on study recruitment criteria:<br>• Based on eligibility criteria: absence of prior diagnostic of breast cancer (Bowen et al [65]; Champion et al [66]; Krist et al [67]; Lin and Wang [68]; Mathieu et al [69]; Seitz et al [71]) with either absence of BRCA1 or BRCA2 mutation, (Seitz et al 2016) or prior abnormal screening test results (Krist et al [67]) or with risk comparable to average risk of population (census data) (Bowen et al [65])<br>• Women invited were identified from the invitation lists of the cancer screening programme(s): Reder et Kolip [70]; Roberto et al [55]<br>➢ Restricted to be at average and/or at low risk through the use of BC risk evaluation instrument: Eden et al [72,73]; Elkin et al [75]; Scariati et al [74]
Ethnicity / rurality (where specified)	➢ Korean American: Lee et al [54]<br>➢ From rural counties: Champion et al [66]; Klippert et Schaper [60]<br>➢ Black or/and African-American: 58-71% (Schapira et al [63]), 33-36% (Walsh et al [64])
Non-participation in previous BCS or barriers to participation §	➢ Have not participated in breast cancer screening (Champion et al, [57]; Krist et al [67]; Lee et al [54]; Schapira et al [63]), with no mammography and with no intention to receive mammography within the next 1- to 2-year period (precontemplators) (Lin and Wang [68])<br>➢ Have not participated in breast cancer screening and CRC screening (Champion et al [56]) or in breast and/or CRC or/and cervical (Champion et al [66]) or in at least 5 preventing measures including BCS (Henry et al [59])<br>➢ Barriers due to ethnicity (Lee et al [54]) or rurality (Champion et al [66]; Klippert et Schaper [60])
Other characteristics¶	➢ Level of education was evaluated to be high (ie, 30-50% with university degree) (Champion et al, [56,57]; Reder et Kolip [70]; Roberto et al [55]; Walsh et al [64]), particularly high (ie, > 50% with a university degree) (Krist et al [61]; Lee et al [54]; Lin and Wang [68]; Mathieu et al [69]) or low (Schapira et al [63])<br>➢ 30-45% of the population was evaluated to be with high income (Champion et al, [56,57,66]; Scariati et al [74])

ACS: American Cancer Society; BC: breast cancer; BCS: breast cancer screening by mammography; CRC: colorectal cancer; USPSTF: the United States Preventive Services Task Force; US: United States; y.o.: years old
*We reported characteristics of the populations who were recruited to participate in the study and access the baseline assessment independently on whether or not individuals used the e-tools and to what extend they used the tool. Characteristics are presented by e-tool (ie, by main study report (see Table S2)); pilot results are not presented.
†BCS recommendation was dependent of women age, country or/and county, and time when the study was conducted. In the US, recommendations vary based on the referent guideline: routine mammography for average-risk women was recommended either from 50 and every 2 years 50 (USPSTF, since 2009), or between 45-54 annually and from 55 every 2 years (ACS, since 2015). Younger women aged 40-44 (ACS) or 40-49 (USPSTF) had the option to start screening with a mammogram every year based on choice and if they belong to high-risk group. In Australia, at the time of the reported study, the national programme was inviting women aged 50–69 years by personal letter; however, upon turning 40 or 70, women were eligible to make an appointment if they wish to start screening earlier or continue screening respectively (Mathieu et al [69]). In other Western countries, all average-risk women aged 50 to 69 years are invited every two years.
§ Non-participation was determined within the last 15 or 24 months (Champion et al [56,66]; Krist et al [67]; Lee et al [54]), in the last 15 months or yearly depending on age (Champion et al, [57]), last year (Schapira et al [63]) or over a defined four months period (Henry et al [59]). Barriers were identified based on previous results and/or studies.
¶Assessed on baseline data or in eligibility criteria (ie, only German speakers (Fissler et al [58]) and no women with Turkish background (Reder et Kolip [70])).

Table S4. Detailed characteristics of the e-tools.

Studies*<br>Name of e-tool (if any)	e-tools characteristics and provided features
**Bowen et al, 2011 [65]**	• Tailored WebApp<br>• Multimedia (images, video, sound, animation, different font sizes)<br>• BCS and heath messages related to diet and sport<br>• Tailoring of the site and its interactive features to each participant: based on baseline phone survey<br>• The App assigned women in a risk group (ie, average, mixed, genetic) based on her risk assignment. Depending on the group, risk messages are different, and the sequence to access the risk page is different
**Champion et al, 2016 [57]**	• Tailored DVD<br>• Video, animations<br>• Tailored messages based on responses provided (through the use of the cursor) regarding beliefs, risks factors and barriers
**Champion et al, 2020 [56]**	• Tailored WebApp<br>• Audio, video Audio dialogue<br>• BCS and CCS<br>• Tailored messages based on the individual’s answers about queries regarding knowledge, perceived and actual risk of BC and CRC, and benefits, barriers, and self-efficacy for both BC and CRC screening<br>• An algorithm embedded in the program directed women at higher-than-average risk for CRC to an intervention that encouraged colonoscopy, whereas women at average risk were allowed to select either stool test or colonoscopy followed by a program consistent with their preferred test
**Champion et al, 2022 [66]**	• Tailored DVD<br>• Tailored messages to address perceived barriers, benefits, self-efficacy, risk and knowledge.<br>• Participants could select to view content on one or multiple cancer screening behaviours at the beginning and had the option to re-watch sections or the entire DVD upon completion<br>• Interactive menus accessed by remote control and allowed participants to select response.
**Eden et al, 2015 [72]**<br><br>“Mammopad”	• Tailored mobile device-optimized WebApp (iPad)<br>• Audio video<br>• Confirm eligibility of the women via risk assessment instrument; women indicating the presence of risk factors or current breast symptoms were informed that they were not eligible for the study<br>• Information modules on breast cancer and mammography<br>• The priority setting module guides users through identifying benefits and harms of BCS screening that are most important to her/him and identify questions and concerns to discuss with providers<br>• Provides a customized report summarizing the user’s screening priorities and intentions for screening, concerns, and questions

**Eden et al, 2020 [73]**<br><br>"MammoScreen"	• Tailored WebApp linked to patient portal<br>• The "Mammopad" e-tool was expanded to include age-specific information about screening for two age groups: 40–49 and 50–74 years<br>• Prepare women for discussions about mammography screening, genetic testing, or risk-reducing medications, based on their responses assessment of familial breast cancer risk<br>• Tailored follow-up messages, based on cancer risk estimates, to participant and clinic<br>• Provide a customized report summarizing next steps, priorities, intentions for screening, and questions or concerns about screening options.
**Elkin et al, 2017 [75]**<br><br>"Breast<br><br>Screening Decisions (BSD)"	• Tailored WebApp<br>• The app provided informed consent, and assessed eligibility criteria<br>• BSD users can explore their attitudes toward BCS<br>• Contains a values-clarification exercise<br>• Contains a breast cancer risk assessment based on the Gail model to identify women at higher risk (who were encouraged to speak with their doctors about their breast cancer risk and screening options)<br>• Provide a page summary of the session, with options to save and print the summary document
**Fissler et al, 2015 [58]**	• Webapp, features-with-tailoring e-tool (for some of the tested conditions)<br>• Mimic a real appointment between the participant and AI-based virtual doctor, with communication through text chat<br>• Tailoring features: for each step of communication, the participants could choose their question or answer from a predetermined selection of text modules. The sequence of interactions patient-doctor was different depending on the physician tested condition. Participants in the "salient needs conditions" filled in a needs questionnaire that made salient their needs about BCS (ie, to receive instructive and useful information in a consultation about mammography screening) and well-being (ie, about how important it is to stay healthy and maintain their status of well-being)
**Henry et al, 2016 [59]**<br><br>"Online Personal Action Plan (oPAP)"	• Tailored WebApp added to an existing patient portal (Kaiser Permanente Southern California (KPSC))<br>• The oPAP synthesizes information from EHRs to provide tailored information and emails users if they need preventive care, including cancer screenings, immunizations, and heart health. It features interactive content with links to enable patient actions<br>• The oPAP flags a user's open care gaps— for five different care gaps: HbA1c testing, pneumonia vaccination, and three cancer screenings—BC, colorectal and cervical. For example, the date of a patient's last breast cancer screening and the date of the next recommended screening—and provides information about specific health conditions, such as links to smoking-cessation programs for smokers and to weight management materials for users with elevated BMI
**Klippert et Schaper, 2018 [60]**	• Features-with-tailoring e-tool, providing Facebook posts (6 posts running for 4 days each except the 2 posts with an external link than ran for one week) to address screening barriers specific to Idaho women, e.g. not understanding personal risk for breast cancer and cost of BCS, using material and wording from the Comprehensive Cancer Alliance for Idaho (CCAI) toolkit<br>• Two posts contained external links— one to the post with the engagement survey including intention to perform BCS) and one with the link to the Women's Health Check (WHC) program, which is a state-funded program that helps low-income and uninsured women in Idaho get mammograms

**Krist et al, 2012 [61]**<br><br>"Interactive preventive health record (IPHR)"	• Tailored e-tool: a new patient portal shared by all the practices involved<br>• The IPHR addressed 18 services recommended by the US Preventive Services Task Force including BCS, cervical and prostate cancer screening<br>• Participants also completed a health risk assessment including race-ethnicity, family history, health behaviours and some past test results not contained in theirs EHRs<br>• Personally tailored list of prevention recommendations, with detailed personal messages that explained and referenced relevant details in the patient's history (e.g. prior laboratory test values and dates), included links to evidence-based educational material and decision aids<br>• Summarized the next steps<br>• The system automatically forwarded a summary to the participant clinician
**Krist et al, 2017 [67]**<br><br>"MyPreventiveCare"	• Seems to be approximately the same tool than Krist et al's [61] e-tool<br>• Tailored e-tool : webApp module embedded in an existing patient portal "MyPreventiveCare" used by all the practices involved<br>• Questions about breast, prostate, colorectal cancer screening that assess personal preferences, knowledge, and needs, and patients 'readiness to make a decision;<br>• Provides personalized educational material tailored to patients' stated preferences and decision stage; allows patients to share their preferences and decision needs with their clinician<br>• Prompts to patients and clinicians to use the reported information to make a decision<br>• The summary included whether patients had made a screening decision, the topics they wanted to discuss, their fears and worries, and their preferred level of decision control<br>• Invites patients and clinicians to provide input after appointment
**Lee et al, 2017 [54]**<br><br>"mMammogram"	• Tailored MobileApp<br>• Targeting content: culture-specific emoticons, graphs, images, pictures, and videos in the Korean language<br>• Tailored: content, number, and timing of daily messages adapted to each individual, health navigator for assistance if necessary (ie, navigating cancer screening information, addressing technical problems, and providing transportation and interpretation services); approximately half of the messages requested a reply<br>• To each response to a question or a prompt, regardless of the answer, the woman could earn a digital pink ribbon and collect these ribbons throughout the intervention period<br>• Embedded in an overall computer system controlling five components: (1) a Web-based application to enrol participants, set user preferences, display the global positioning system (GPS) navigation system with area clinic and triggers to book screening appointment information, and upload text and multimedia messages; (2) database to store participant records, rules, and messages sent and received; (3) a program to establish the appropriate timing of messages, determine which messages to send, and process received replies; (4) a text-message delivery or reception platform; and (5) a health navigator for assistance navigating cancer screening information, addressing technical problems, and providing transportation and interpretation services.

**Lin and Wang, 2009 [68]**	• Tailored WebApp with tailoring features<br>• With both complete tailored intervention (CTI) and tailored intervention (TI) e-tools, woman received computer-generated messages tailored to her Decisional Balance for Mammography Inventory (DBMI) responses. In addition, with the CTI e-tool, women receive personalized list of messages and activities (education, personal testimonies, and role modelling) designed to the precontemplation stage of mammography adoption and developed based upon the TTM change<br>• Embedded in an IT system verifying women eligibility, women intention to have a mammography, breast cancer history, mammography history, and ensuring random assignment to intervention
**Mathieu et al, 2010 [69]**	• WebApp, features-with-tailoring e-tool<br>• Include a worksheet to assist women to integrate the outcomes of BCS with their personal values and preferences
**Pereira et al, 2020 [62]**<br><br>"AllAboutBreastCancer"	• Features-with-tailoring e-tool, which is based on a WhatApp group<br>• Topics: risk factors, protective factors, definitions, incidence, clinical signs and symptoms, diagnostic examinations, mammography, myths and truths, and places to seek support. Additional media were created or adapted by the team, according to the demands and dynamics of the group<br>• Daily sessions of text, figure, video, and voice messages during 3 weeks. In total, two videos, 23 images, and four audios were utilized. The content was selected from the Brazilian National Cancer Institute and the Brazilian Ministry of Health websites
**Reder et Kolip, 2017 [70]**	• Webapp, features-with-tailoring e-tool<br>• Static (ie, not interactive) information part (comparing to the brochure, it contains pictograms to support absolute numbers)<br>• With an interactive part, which is the value clarification exercise: women are asked to assign the information items to categories, rate the importance of each information item, and make a choice. A graphical summary of personal responses is provided
**Roberto et al, 2020 [55]**<br>"Donnainformata-Mammografia"	• Webapp, features-with-tailoring e-tool<br>• Static (ie, not interactive) information part and interactive section<br>• Information similar to the brochure; in addition it contains items that are not in the brochure notably absolute numbers for over diagnostic, controversy and disagreement on quantification of harms and benefits (Quantitative estimates from Cochrane Review), and information on prevention (One screen on risk and protective factors with a table comparing things to do and not to do)<br>• Contains a value clarification exercise page, that can be printed: the e-tool lists issues and concerns, e.g. values, experience, and perception of the risk of developing BC, that can affect screening decision, and each woman was asked to state the importance of each item for her
**Scariati et al, 2015 [74]**	• Tailored WebApp<br>• A series of risk stratification questions aimed at identifying women with an increased risk of developing BC; those are directed to appropriate screening and follow-up and do not access the e-tool<br>• Provides a values clarification exercise divided into two parts. In the first one, the woman is asked to identify the ten factors that matter the most to her. In the second part, the woman rates these ten factors into one of three categories of importance for her: most important, moderate, or least important<br>• The value clarification exercise was then captured and included as part of a summary that the woman received

**Schapira et al, 2019 [63]**<br><br>"Breast cancer screening patient decision-aid : BCS-PtDA"	• Tailored WebApp<br>• No video, no audio, with pictograms and untailored exemplars<br>• Similar to Seitz et al's e-tool 'extended condition and untailored exemplars' [71]: 3 exemplars (woman at low, medium and high risk) are displayed to all women in a random order<br>• Provide an interactive summary sheet: intention and value clarification exercise
**Seitz et al, 2016 [71]**	• Tailored WebApp: the highest level of tailoring was provided with tailored exemplars (see below)<br>• The DA component was embedded in a web application in charge of verifying eligibility, ensuring baseline survey, and BC risk calculation, assignment of the participant in the average risk or high risk group, and random assignment to the one of the six conditions (ie, formally different e-tools), within each risk group, and post-survey<br>• Different module depending on the tested condition/e-tool ; all modules contain basic info on mammography (the definition of mammography, a statement that women between the ages of 40 and 50 years old have a choice to make about when to begin mammography, screening options, and recommendations from the ACS and the USPSTF) plus extra information either brief (individualized 10-year and lifetime estimates of their objective risk for developing BC and the risk of an average-risk age-matched woman) or extended (brief info plus how their risk compared to a typical 50-year-old woman, statistics about outcomes of mammography (e.g., rates of false positives and mammogram-detected cancers), and information about effects of mammography on reducing BC mortality). Arguments to have mammography (ie, whether to wait up 50 in the average risk group or "not wait and proceed" in the high risk group), were given in different ways: expository (no exemplars) or with exemplars. Exemplars are examples of women making decisions about mammography, and are either untailored or tailored (with similar age, BC risk, family history of BC, and parity) to the participant
**Walsh et al, 2020 [64]**<br><br>"PreView" (the PREventive<br><br>Video Education in Waiting Rooms Program)	• Tailored Computer App (iPad)<br>• Multimedia (Video and audio)<br>• Simulates a conversation between user and an AI-based virtual doctor<br>• Breast, colon, prostate, cervical cancer and screening education modules<br>• The app questions about demographics, health history, and prior cancer screening, screening stage of change and perceived barriers to screening, and provides individualized messages/video clips based on woman's stage of change and individual screening barriers for each type cancer<br>• Generates a 'Provider Alert' (ie, for each cancer, a printed sheet summarizing the individual patient's screening history, his/her individual readiness to change, and his/her individual barriers (roadblock) and suggestions to the physician for appropriate roadblock-related messages)

BMI: body mass index; BC: breast cancer; CRC: colorectal cancer; BCS breast cancer screening; AI: artificial intelligence; TTM: Transtheoretical Model of Behaviour Change; EHR: electronic health record; webApp : a web-based application (ie, an application software that runs on a web server)
* Characteristics are presented by e-tool (ie, by main study report (see Table S2)); studies reports are presented by alphabetical order (first author). Pilot results are not presented

Note: in all the following figures S1-S8, risk of bias was assessed using the revised Cochrane-risk-of-bias-2 for randomized trials RoB 2 tool [52], with each domain (A: randomization process, B: deviations from the intended protocol, C: missing data, D: outcome measurement, and E: reporting results) and the overall risk (F) evaluated as low (green), moderate (yellow), or high (red).

Figure S1. Participation in breast cancer screening (BCS) assessed at short-term.

A. Efficacy of the e-tools compared to control on women' participation in breast cancer screening (behaviour) measured at short term (ie, up to 6 months)

E-tools did not increase women's participation in BCS at short term compared to the control group (ie, either usual care (Champion et al [56, 57]; Krist et al [61]; Lee et al [54]; Reder and Kolip [70]) or a control website [55])

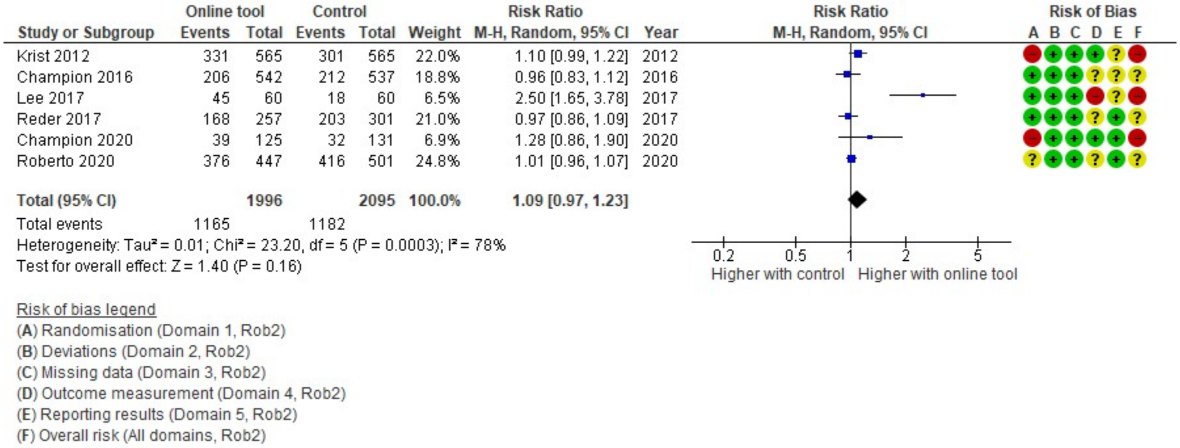

B. Exclusion of Lee et al's study [54] (Mobile e-tool) decreases heterogeneity without changing the results

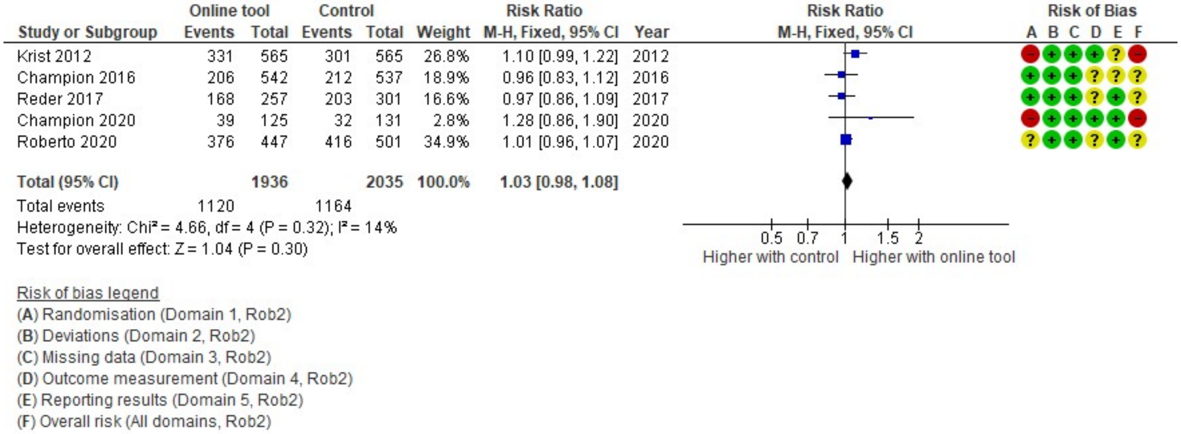

C. Other analysis: exclusion

***Exclusion of Roberto et al's study [55] (website as control): no effect**

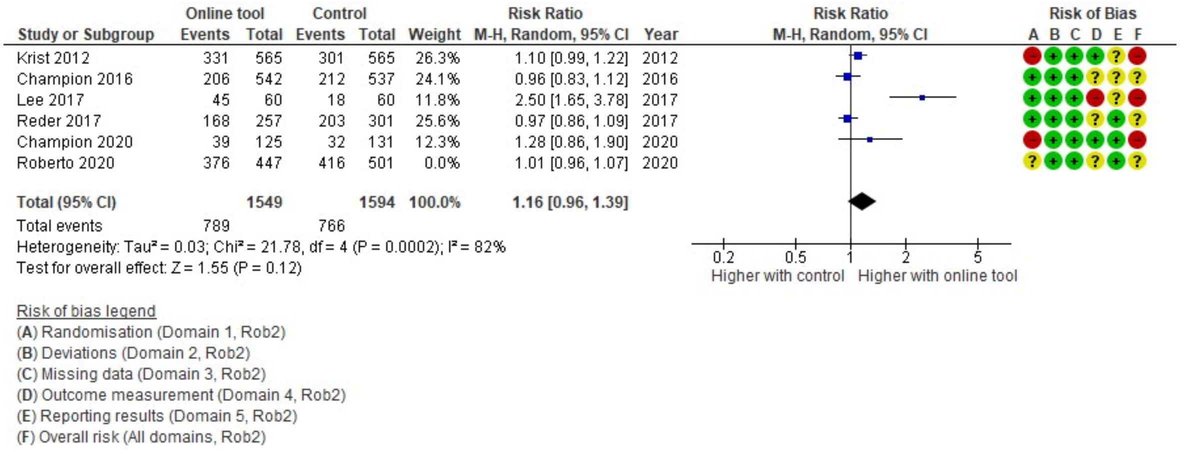

*** Exclusion of Krist et al's e-tool (Portal) [61]: no effect**

D. Subgroup analysis based on the nature of the tools (ie, tailored tools vs features-with-tailoring e-tools): no subgroup difference

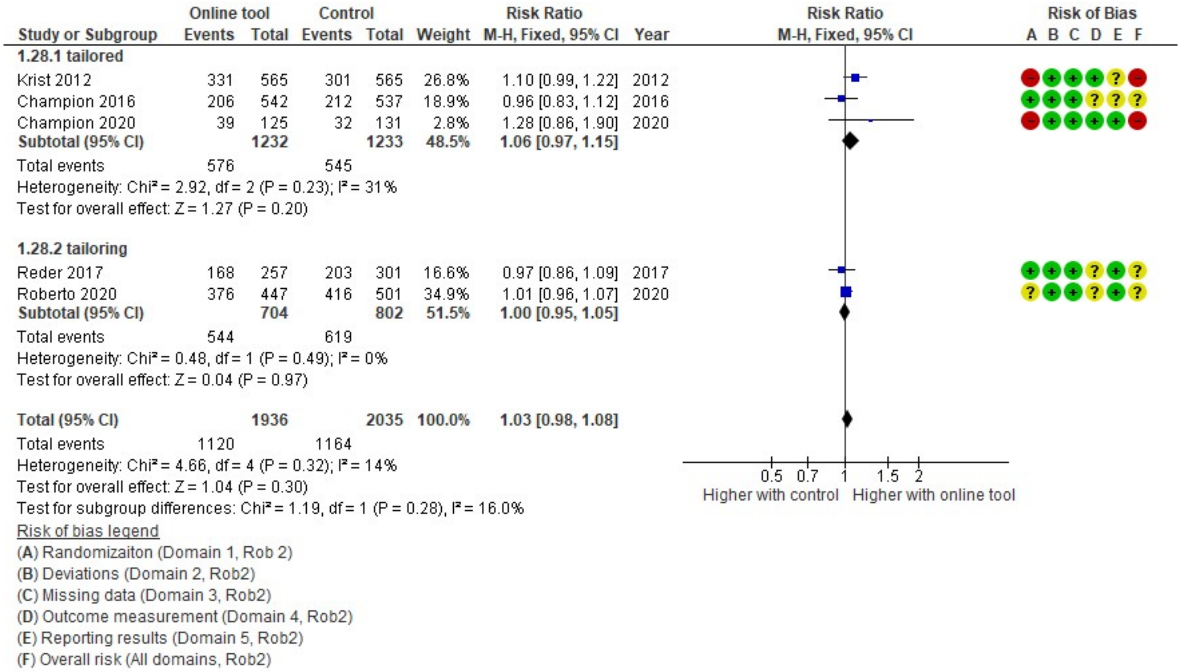

Figure S2. Participation in BCS assessed at long-term: effect of subgroup analysis. Subgroups analysis with different variables did not highlight any subgroup differences (not shown) except for the nature of the tailoring component (below); test for subgroup differences suggests that there is a statistically significant subgroup effect (P < 0.0001) that seems to be quantitative with the participation being higher with tailoring not to risk e-tools compared with tailored to risk e-tools (see Table 2 in the full JMIR manuscript). However, there is a substantial unexplained heterogeneity between the trials of one of the subgroups decreasing the robustness of this result (risk-based group: I^2 = 0%; behaviour-change based group: I^2= 98%).

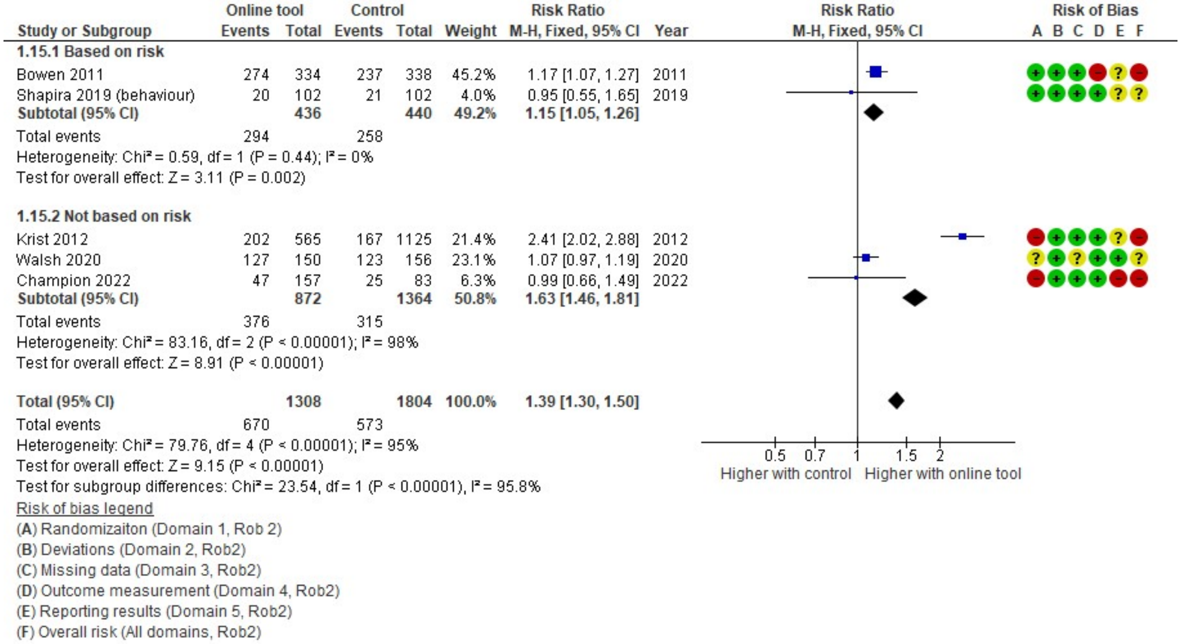

Figure S3. Intention subgroup analysis (ie, tailored tools vs features-with-tailoring e-tools). Here, analyses were performed with Li and Wang's "tailored message intervention" (TMI) e-tool [68] and Seitz et al's "extended information with untailored exemplars" e-tool [71]

	Online tool		Control			Risk Ratio		Risk of Bias					
**Study or Subgroup**	**Events**	**Total**	**Events**	**Total**	**Weight**	**M-H, Random, 95% CI**	**Year**	**A**	**B**	**C**	**D**	**E**	**F**
**1.2.1 Features-with-tailoring e-tools**													
Mathieu 2010	50	117	84	209	2.5%	1.06 [0.81, 1.39]	2010	?	+	+	?	?	?
Reder 2017	190	278	280	390	13.7%	0.95 [0.86, 1.05]	2017	+	+	+	?	+	?
Roberto 2020	461	472	502	529	49.9%	1.03 [1.00, 1.05]	2020	?	+	+	?	+	?
**Subtotal (95% CI)**		**867**		**1128**	**66.1%**	**1.01 [0.93, 1.09]**							
Total events	701		866										
Heterogeneity: Tau² = 0.00; Chi² = 4.13, df = 2 (P = 0.13); I² = 52%													
Test for overall effect: Z = 0.16 (P = 0.87)													
**1.2.2 Tailored e-tools**													
Lin 2009	43	63	29	61	1.8%	1.44 [1.05, 1.96]	2009	?	+	+	+	+	+
Seitz 2016	306	359	293	346	26.4%	1.01 [0.95, 1.07]	2016	?	+	−	+	?	−
Lee 2017	50	60	48	60	5.8%	1.04 [0.88, 1.23]	2017	+	+	+	−	?	−
**Subtotal (95% CI)**		**482**		**467**	**33.9%**	**1.08 [0.93, 1.26]**							
Total events	399		370										
Heterogeneity: Tau² = 0.01; Chi² = 5.34, df = 2 (P = 0.07); I² = 63%													
Test for overall effect: Z = 0.99 (P = 0.32)													
**Total (95% CI)**		**1349**		**1595**	**100.0%**	**1.02 [0.98, 1.06]**							
Total events	1100		1236										
Heterogeneity: Tau² = 0.00; Chi² = 7.21, df = 5 (P = 0.21); I² = 31%													
Test for overall effect: Z = 0.90 (P = 0.37)													
Test for subgroup differences: Chi² = 0.64, df = 1 (P = 0.43), I² = 0%													

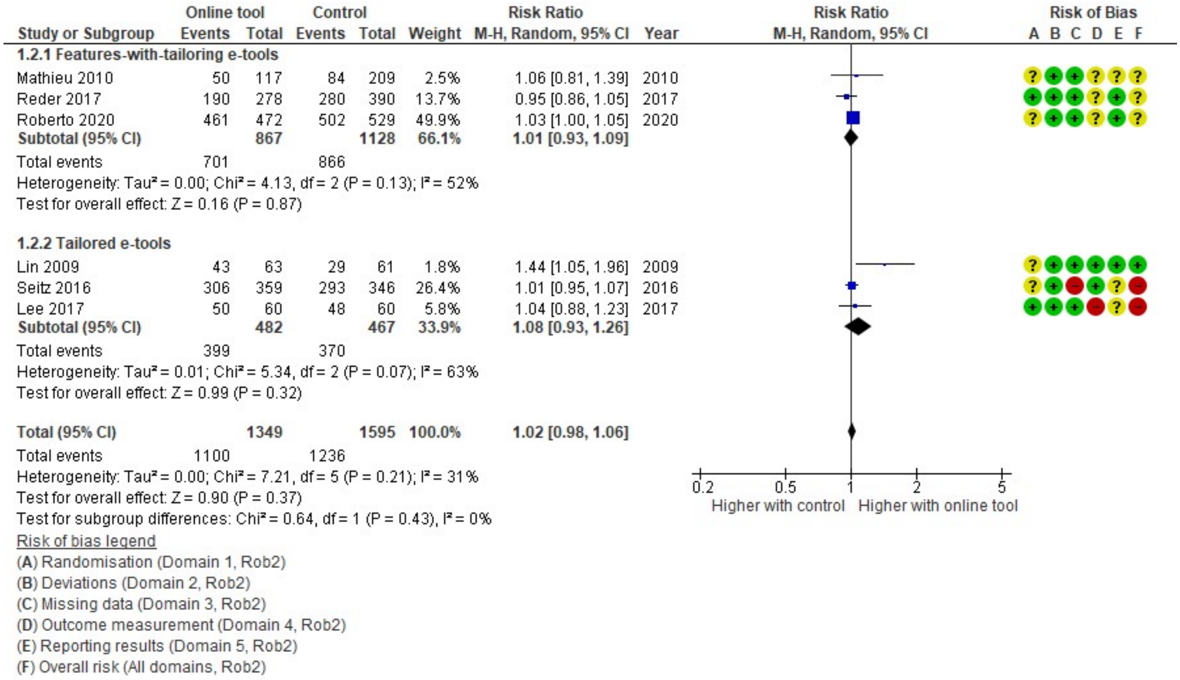

Risk of bias legend
(A) Randomisation (Domain 1, Rob2)
(B) Deviations (Domain 2, Rob2)
(C) Missing data (Domain 3, Rob2)
(D) Outcome measurement (Domain 4, Rob2)
(E) Reporting results (Domain 5, Rob2)
(F) Overall risk (All domains, Rob2)

Figure S4. Adequate knowledge.

A. With all included studies

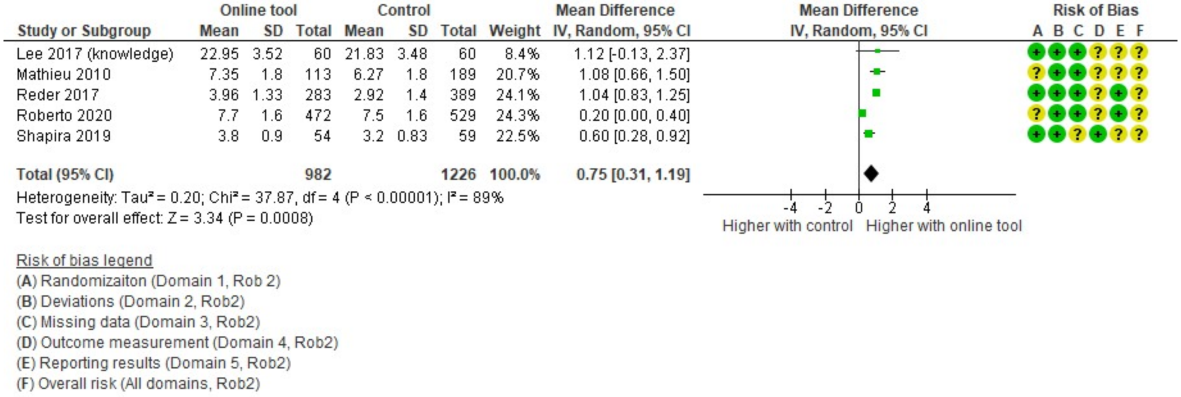

B. Exclusion of Roberto et al's study [55]

Analyses to investigate source of heterogeneity (ie, study exclusion based on specific variables of interest), did not change the result nor decrease heterogeneity, except when Roberto et al's study [55], which used a website as comparator, was excluded:

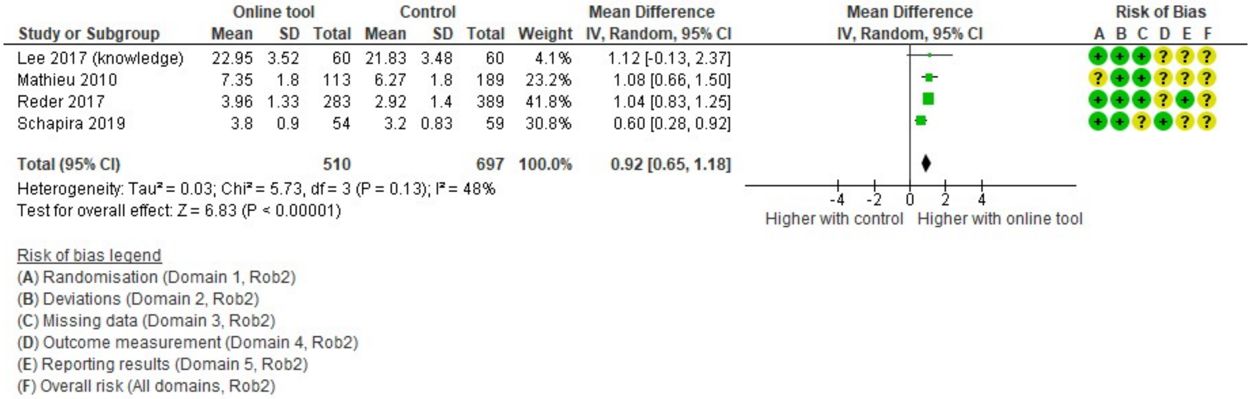

C. Subgrouping analysis including Roberto et al's study [55]

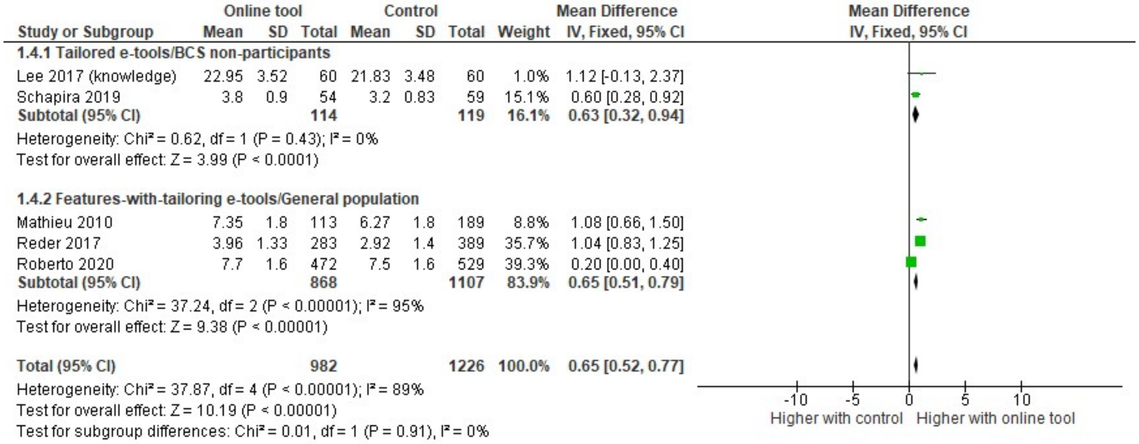

Figure S5. Attitudes: rates of women with positive attitude toward undergoing BCS. Control was usual care except for Roberto et al's study (website) [55]

Study or Subgroup	Online tool Events	Online tool Total	Control Events	Control Total	Weight	Risk Ratio M-H, Fixed, 95% CI	Risk of Bias A	B	C	D	E	F
Mathieu 2010	88	111	144	182	12.7%	1.00 [0.89, 1.13]	?	+	+	?	?	?
Reder 2017	235	283	342	391	33.5%	0.95 [0.89, 1.01]	+	+	+	?	+	?
Roberto 2020	432	472	489	529	53.8%	0.99 [0.95, 1.03]	?	+	+	?	+	?
**Total (95% CI)**		**866**		**1102**	**100.0%**	**0.98 [0.95, 1.01]**						
Total events	755		975									

Heterogeneity: Chi² = 1.40, df = 2 (P = 0.50); I² = 0%
Test for overall effect: Z = 1.32 (P = 0.19)

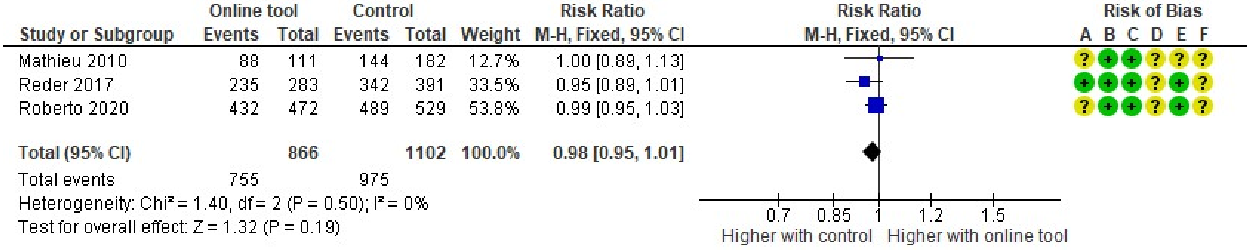

Risk of bias legend
(**A**) Randomisation (Domain 1, Rob2)
(**B**) Deviations (Domain 2, Rob2)
(**C**) Missing data (Domain 3, Rob2)
(**D**) Outcome measurement (Domain 4, Rob2)
(**E**) Reporting results (Domain 5, Rob2)
(**F**) Overall risk (All domains, Rob2)

Figure S6. Worry when using different Seitz et al's tailored messages/e-tools [71]

Brief information plus expository (no exemplars)

Study or Subgroup	Experimental Mean	Experimental SD	Experimental Total	Control Mean	Control SD	Control Total	Weight	Mean Difference IV, Fixed, 95% CI
Seitz 2016	2.44	1.24	351	2.12	1.19	346	93.9%	0.32 [0.14, 0.50]
Shapira 2019	5.4	1.94	54	5	1.88	59	6.1%	0.40 [-0.31, 1.11]
**Total (95% CI)**			**405**			**405**	**100.0%**	**0.32 [0.15, 0.50]**

Heterogeneity: Chi² = 0.05, df = 1 (P = 0.83); I² = 0%
Test for overall effect: Z = 3.64 (P = 0.0003)

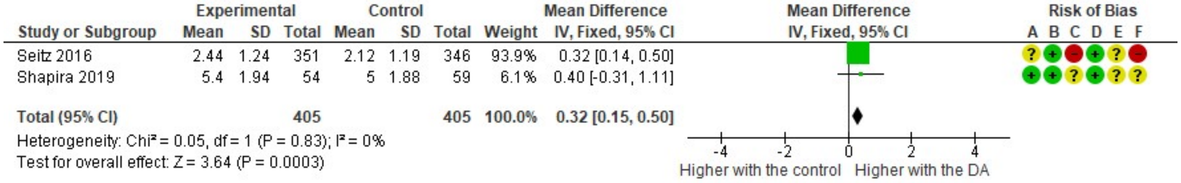

Risk of bias legend
(A) Randomizaiton (Domain 1, Rob 2)
(B) Deviations (Domain 2, Rob2)
(C) Missing data (Domain 3, Rob2)
(D) Outcome measurement (Domain 4, Rob2)
(E) Reporting results (Domain 5, Rob2)
(F) Overall risk (All domains, Rob2)

Brief information plus untailored exemplars

Study or Subgroup	Experimental Mean	Experimental SD	Experimental Total	Control Mean	Control SD	Control Total	Weight	Mean Difference IV, Fixed, 95% CI
Seitz 2016	2.36	1.29	364	2.12	1.19	346	93.7%	0.24 [0.06, 0.42]
Shapira 2019	5.4	1.94	54	5	1.88	59	6.3%	0.40 [-0.31, 1.11]
**Total (95% CI)**			**418**			**405**	**100.0%**	**0.25 [0.07, 0.43]**

Heterogeneity: Chi² = 0.19, df = 1 (P = 0.67); I² = 0%
Test for overall effect: Z = 2.77 (P = 0.006)

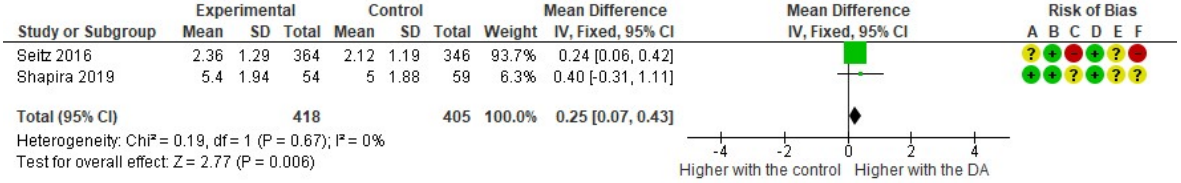

Risk of bias legend
(A) Randomizaiton (Domain 1, Rob 2)
(B) Deviations (Domain 2, Rob2)
(C) Missing data (Domain 3, Rob2)
(D) Outcome measurement (Domain 4, Rob2)
(E) Reporting results (Domain 5, Rob2)
(F) Overall risk (All domains, Rob2)

Brief information plus tailored exemplars

Study or Subgroup	Experimental Mean	Experimental SD	Experimental Total	Control Mean	Control SD	Control Total	Weight	Mean Difference IV, Fixed, 95% CI
Seitz 2016	2.38	1.22	321	2.12	1.19	346	93.7%	0.26 [0.08, 0.44]
Shapira 2019	5.4	1.94	54	5	1.88	59	6.3%	0.40 [-0.31, 1.11]
**Total (95% CI)**			**375**			**405**	**100.0%**	**0.27 [0.09, 0.45]**

Heterogeneity: Chi² = 0.14, df = 1 (P = 0.71); I² = 0%
Test for overall effect: Z = 2.97 (P = 0.003)

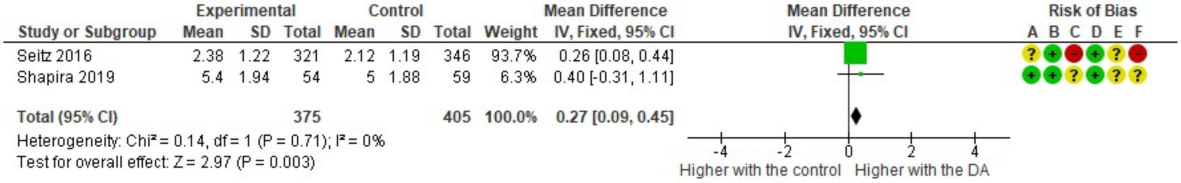

Risk of bias legend
(A) Randomizaiton (Domain 1, Rob 2)
(B) Deviations (Domain 2, Rob2)
(C) Missing data (Domain 3, Rob2)
(D) Outcome measurement (Domain 4, Rob2)
(E) Reporting results (Domain 5, Rob2)
(F) Overall risk (All domains, Rob2)

Extended information with expository (no exemplars)

Study or Subgroup	Experimental Mean	Experimental SD	Experimental Total	Control Mean	Control SD	Control Total	Weight	Mean Difference IV, Fixed, 95% CI	A	B	C	D	E	F
Seitz 2016	2.37	1.2	372	2.12	1.19	346	94.2%	0.25 [0.08, 0.42]	?	+	−	+	?	−
Shapira 2019	5.4	1.94	54	5	1.88	59	5.8%	0.40 [-0.31, 1.11]	+	+	?	+	?	?
**Total (95% CI)**			**426**			**405**	**100.0%**	**0.26 [0.09, 0.43]**						

Heterogeneity: Chi² = 0.16, df = 1 (P = 0.69); I² = 0%
Test for overall effect: Z = 2.99 (P = 0.003)

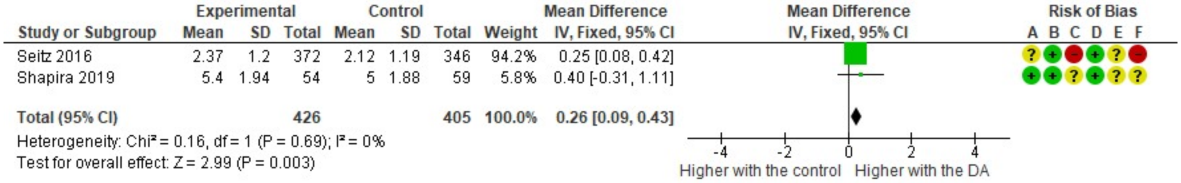

Risk of bias legend
(A) Randomizaiton (Domain 1, Rob 2)
(B) Deviations (Domain 2, Rob2)
(C) Missing data (Domain 3, Rob2)
(D) Outcome measurement (Domain 4, Rob2)
(E) Reporting results (Domain 5, Rob2)
(F) Overall risk (All domains, Rob2)

Extended information with tailored exemplars

Study or Subgroup	Experimental Mean	Experimental SD	Experimental Total	Control Mean	Control SD	Control Total	Weight	Mean Difference IV, Fixed, 95% CI	A	B	C	D	E	F
Seitz 2016	2.31	1.25	352	2.12	1.19	346	93.8%	0.19 [0.01, 0.37]	?	+	−	+	?	−
Shapira 2019	5.4	1.94	54	5	1.88	59	6.2%	0.40 [-0.31, 1.11]	+	+	?	+	?	?
**Total (95% CI)**			**406**			**405**	**100.0%**	**0.20 [0.03, 0.38]**						

Heterogeneity: Chi² = 0.32, df = 1 (P = 0.57); I² = 0%
Test for overall effect: Z = 2.27 (P = 0.02)

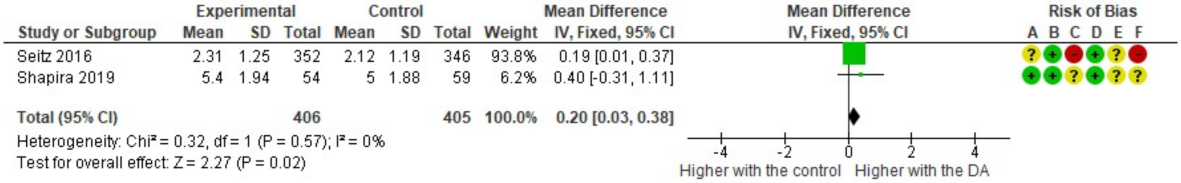

Risk of bias legend
(A) Randomizaiton (Domain 1, Rob 2)
(B) Deviations (Domain 2, Rob2)
(C) Missing data (Domain 3, Rob2)
(D) Outcome measurement (Domain 4, Rob2)
(E) Reporting results (Domain 5, Rob2)
(F) Overall risk (All domains, Rob2)

Figure S7. Accuracy of risk when using different Seitz et al's e-tools/tailored messages [71].

Brief information plus expository (no exemplars)

Study or Subgroup	Experimental Mean	Experimental SD	Experimental Total	Control Mean	Control SD	Control Total	Weight	Mean Difference IV, Random, 95% CI	Risk of Bias A	B	C	D	E	F
Seitz 2016	11.2	25.2	351	10.6	19	346	69.2%	0.60 [-2.71, 3.91]	?	+	-	+	?	-
Shapira 2019	3.3	21.2	54	9.3	25.8	59	30.8%	-6.00 [-14.68, 2.68]	+	+	?	+	?	?
**Total (95% CI)**			**405**			**405**	**100.0%**	**-1.43 [-7.40, 4.54]**						

Heterogeneity: Tau² = 10.55; Chi² = 1.94, df = 1 (P = 0.16); I² = 48%
Test for overall effect: Z = 0.47 (P = 0.64)

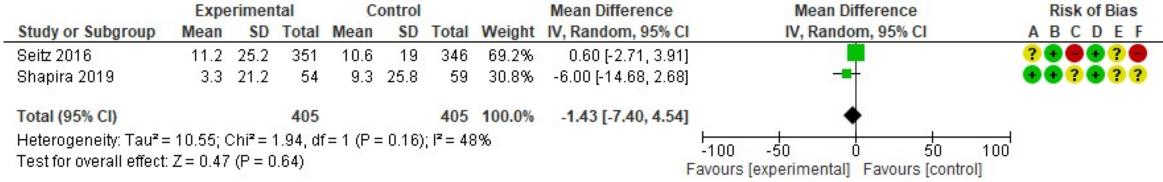

Risk of bias legend
(A) Randomizaiton (Domain 1, Rob 2)
(B) Deviations (Domain 2, Rob2)
(C) Missing data (Domain 3, Rob2)
(D) Outcome measurement (Domain 4, Rob2)
(E) Reporting results (Domain 5, Rob2)
(F) Overall risk (All domains, Rob2)

Brief information plus untailored exemplars

Study or Subgroup	Experimental Mean	Experimental SD	Experimental Total	Control Mean	Control SD	Control Total	Weight	Mean Difference IV, Fixed, 95% CI	Risk of Bias A	B	C	D	E	F
Seitz 2016	9.7	23.5	364	10.6	19	346	88.4%	-0.90 [-4.04, 2.24]	?	+	-	+	?	-
Shapira 2019	3.3	21.2	54	9.3	25.8	59	11.6%	-6.00 [-14.68, 2.68]	+	+	?	+	?	?
**Total (95% CI)**			**418**			**405**	**100.0%**	**-1.49 [-4.44, 1.46]**						

Heterogeneity: Chi² = 1.17, df = 1 (P = 0.28); I² = 15%
Test for overall effect: Z = 0.99 (P = 0.32)

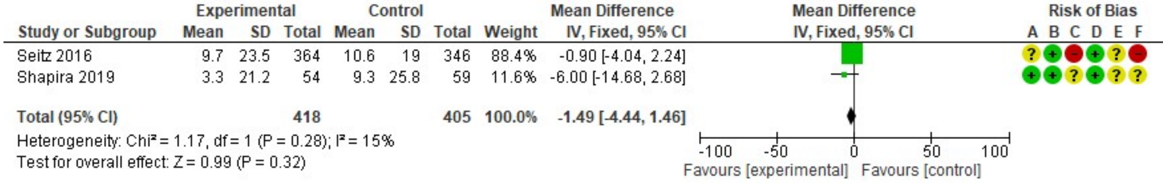

Risk of bias legend
(A) Randomizaiton (Domain 1, Rob 2)
(B) Deviations (Domain 2, Rob2)
(C) Missing data (Domain 3, Rob2)
(D) Outcome measurement (Domain 4, Rob2)
(E) Reporting results (Domain 5, Rob2)
(F) Overall risk (All domains, Rob2)

Brief information plus tailored exemplars

Study or Subgroup	Experimental Mean	Experimental SD	Experimental Total	Control Mean	Control SD	Control Total	Weight	Mean Difference IV, Random, 95% CI	Risk of Bias A	B	C	D	E	F
Seitz 2016	12.6	28.1	321	10.6	19	346	62.6%	2.00 [-1.67, 5.67]	?	+	-	+	?	-
Shapira 2019	3.3	21.2	54	9.3	25.8	59	37.4%	-6.00 [-14.68, 2.68]	+	+	?	+	?	?
**Total (95% CI)**			**375**			**405**	**100.0%**	**-0.99 [-8.58, 6.59]**						

Heterogeneity: Tau² = 20.45; Chi² = 2.77, df = 1 (P = 0.10); I² = 64%
Test for overall effect: Z = 0.26 (P = 0.80)

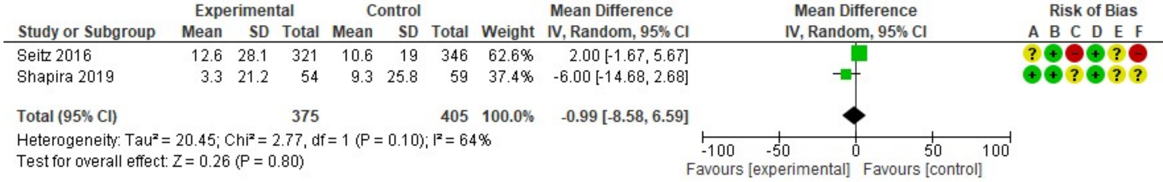

Risk of bias legend
(A) Randomizaiton (Domain 1, Rob 2)
(B) Deviations (Domain 2, Rob2)
(C) Missing data (Domain 3, Rob2)
(D) Outcome measurement (Domain 4, Rob2)
(E) Reporting results (Domain 5, Rob2)
(F) Overall risk (All domains, Rob2)

Extended information with expository (no exemplars)

Study or Subgroup	Experimental Mean	Experimental SD	Experimental Total	Control Mean	Control SD	Control Total	Weight	Mean Difference IV, Fixed, 95% CI	Risk of Bias A	B	C	D	E	F
Seitz 2016	9.7	25.3	372	10.6	19	346	87.6%	-0.90 [-4.16, 2.36]	?	+	-	+	?	-
Shapira 2019	3.3	21.2	54	9.3	25.8	59	12.4%	-6.00 [-14.68, 2.68]	+	+	?	+	?	?
**Total (95% CI)**			**426**			**405**	**100.0%**	**-1.53 [-4.58, 1.52]**						

Heterogeneity: Chi² = 1.16, df = 1 (P = 0.28); I² = 14%
Test for overall effect: Z = 0.98 (P = 0.33)

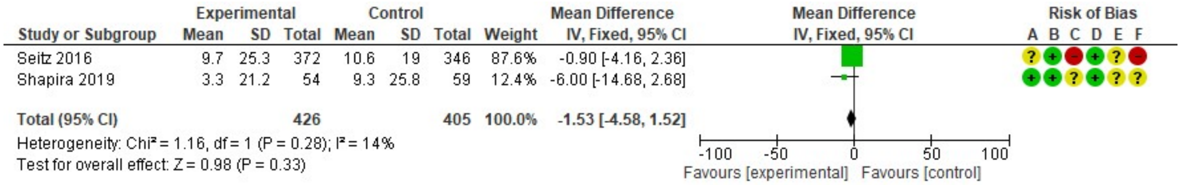

Risk of bias legend
(A) Randomizaiton (Domain 1, Rob 2)
(B) Deviations (Domain 2, Rob2)
(C) Missing data (Domain 3, Rob2)
(D) Outcome measurement (Domain 4, Rob2)
(E) Reporting results (Domain 5, Rob2)
(F) Overall risk (All domains, Rob2)

Extended information with tailored exemplars

Study or Subgroup	Experimental Mean	Experimental SD	Experimental Total	Control Mean	Control SD	Control Total	Weight	Mean Difference IV, Fixed, 95% CI	Risk of Bias A	B	C	D	E	F
Seitz 2016	8.3	23.6	359	10.6	19	346	88.3%	-2.30 [-5.46, 0.86]	?	+	-	+	?	-
Shapira 2019	3.3	21.2	54	9.3	25.8	59	11.7%	-6.00 [-14.68, 2.68]	+	+	?	+	?	?
**Total (95% CI)**			**413**			**405**	**100.0%**	**-2.73 [-5.70, 0.23]**						

Heterogeneity: Chi² = 0.62, df = 1 (P = 0.43); I² = 0%
Test for overall effect: Z = 1.81 (P = 0.07)

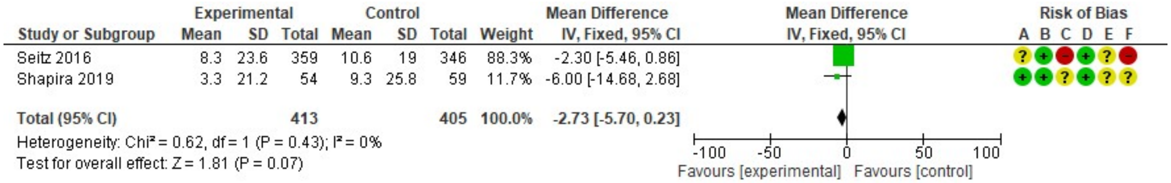

Risk of bias legend
(A) Randomizaiton (Domain 1, Rob 2)
(B) Deviations (Domain 2, Rob2)
(C) Missing data (Domain 3, Rob2)
(D) Outcome measurement (Domain 4, Rob2)
(E) Reporting results (Domain 5, Rob2)
(F) Overall risk (All domains, Rob2)

Figure S8. Informed choice-related dimensions.

A. Adequate knowledge analysis performed with studies for which informed choice results were obtained, with (upper) and without (lower) Roberto et al's study [55] (using website as control)

	DA			Control				Mean Difference
Study or Subgroup	Mean	SD	Total	Mean	SD	Total	Weight	IV, Random, 95% CI
Mathieu 2010	7.35	1.8	113	6.27	1.8	189	30.9%	1.08 [0.66, 1.50]
Reder 2017	3.96	1.33	283	2.92	1.4	389	34.5%	1.04 [0.83, 1.25]
Roberto 2020	7.7	1.6	472	7.5	1.6	529	34.6%	0.20 [0.00, 0.40]
**Total (95% CI)**			**868**			**1107**	**100.0%**	**0.76 [0.13, 1.39]**

Heterogeneity: $Tau^2 = 0.29$; $Chi^2 = 37.24$, df = 2 (P < 0.00001); $I^2 = 95\%$
Test for overall effect: Z = 2.37 (P = 0.02)

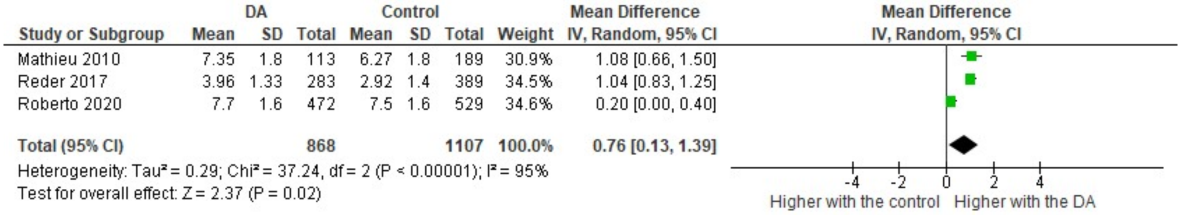

	DA			Control				Mean Difference	Risk of Bias					
Study or Subgroup	Mean	SD	Total	Mean	SD	Total	Weight	IV, Fixed, 95% CI	A	B	C	D	E	F
Mathieu 2010	7.35	1.8	113	6.27	1.8	189	19.8%	1.08 [0.66, 1.50]	?	+	+	?	?	?
Reder 2017	3.96	1.33	283	2.92	1.4	389	80.2%	1.04 [0.83, 1.25]	+	+	+	?	+	?
Roberto 2020	7.7	1.6	472	7.5	1.6	529	0.0%	0.20 [0.00, 0.40]	?	+	+	?	+	?
**Total (95% CI)**			**396**			**578**	**100.0%**	**1.05 [0.86, 1.23]**						

Heterogeneity: $Chi^2 = 0.03$, df = 1 (P = 0.87); $I^2 = 0\%$
Test for overall effect: Z = 11.01 (P < 0.00001)

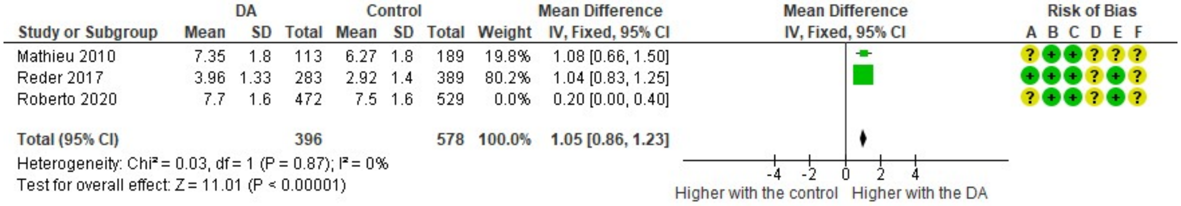

Risk of bias legend
(A) Randomizaiton (Domain 1, Rob 2)
(B) Deviations (Domain 2, Rob2)
(C) Missing data (Domain 3, Rob2)
(D) Outcome measurement (Domain 4, Rob2)
(E) Reporting results (Domain 5, Rob2)
(F) Overall risk (All domains, Rob2)

B. Intention analysis performed with studies for which informed choice results were obtained, with (upper) et without (lower) Roberto's et al's study (using website as control) [55]

	e-tool		Control			Risk Ratio		Risk of Bias					
Study or Subgroup	Events	Total	Events	Total	Weight	M-H, Fixed, 95% CI	Year	A	B	C	D	E	F
Mathieu 2010	50	117	84	209	7.9%	1.06 [0.81, 1.39]	2010	?	+	+	?	?	?
Reder 2017	190	278	280	390	30.4%	0.95 [0.86, 1.05]	2017	+	+	+	?	+	?
Roberto 2020	461	472	502	529	61.7%	1.03 [1.00, 1.05]	2020	?	+	+	?	+	?
**Total (95% CI)**		**867**		**1128**	**100.0%**	**1.01 [0.97, 1.05]**							
Total events	701		866										

Heterogeneity: $Chi^2 = 4.13$, df = 2 (P = 0.13); $I^2 = 52\%$
Test for overall effect: Z = 0.41 (P = 0.68)

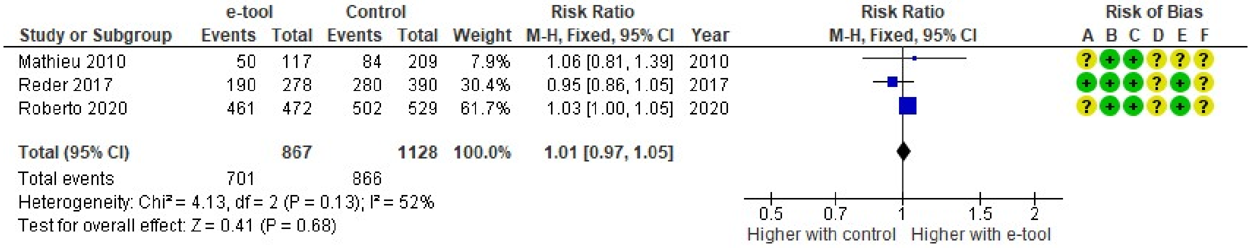

Risk of bias legend
(A) Randomisation (Domain 1, Rob2)
(B) Deviations (Domain 2, Rob2)
(C) Missing data (Domain 3, Rob2)
(D) Outcome measurement (Domain 4, Rob2)
(E) Reporting results (Domain 5, Rob2)
(F) Overall risk (All domains, Rob2)

	e-tool		Control			Risk Ratio		Risk of Bias					
Study or Subgroup	Events	Total	Events	Total	Weight	M-H, Fixed, 95% CI	Year	A	B	C	D	E	F
Mathieu 2010	50	117	84	209	20.6%	1.06 [0.81, 1.39]	2010	?	+	+	?	?	?
Reder 2017	190	278	280	390	79.4%	0.95 [0.86, 1.05]	2017	+	+	+	?	+	?
Roberto 2020	461	472	502	529	0.0%	1.03 [1.00, 1.05]	2020	?	+	+	?	+	?
**Total (95% CI)**		**395**		**599**	**100.0%**	**0.97 [0.88, 1.08]**							
Total events	240		364										

Heterogeneity: Chi² = 0.62, df = 1 (P = 0.43); I² = 0%
Test for overall effect: Z = 0.51 (P = 0.61)

Risk Ratio
M-H, Fixed, 95% CI
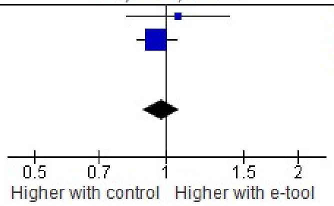

Risk of bias legend
(A) Randomisation (Domain 1, Rob2)
(B) Deviations (Domain 2, Rob2)
(C) Missing data (Domain 3, Rob2)
(D) Outcome measurement (Domain 4, Rob2)
(E) Reporting results (Domain 5, Rob2)
(F) Overall risk (All domains, Rob2)

Note: The references cited in this "Multimedia Appendix 1" are numbered as in the full JMIR manuscript.

References

47. Deeks JJ HJ, Altman DG (editors). Chapter 10: Analysing data and undertaking meta-analyses In: Higgins JPT TJ, Chandler J, Cumpston M, Li T, Page MJ, Welch VA editors. Cochrane Handbook for Systematic Reviews of Interventions version 64 (updated August 2023) Cochrane Available from wwwtrainingcochraneorg/handbook. 2023. ISBN.

54. Lee H, Ghebre R, Le C, Jang YJ, Sharratt M, Yee D. Mobile Phone Multilevel and Multimedia Messaging Intervention for Breast Cancer Screening: Pilot Randomized Controlled Trial. JMIR mHealth and uHealth. 2017;5(11):e154. doi: 10.2196/mhealth.7091.

55. Roberto A, Colombo C, Candiani G, Satolli R, Giordano L, Jaramillo L, et al. A dynamic web-based decision aid to improve informed choice in organised breast cancer screening. A pragmatic randomised trial in Italy. British Journal of Cancer. 2020;123(5):714-721. doi: 10.1038/s41416-020-0935-2.

56. Champion VL, Christy SM, Rakowski W, Lairson DR, Monahan PO, Gathirua-Mwangi WG, et al. An RCT to Increase Breast and Colorectal Cancer Screening. Am J Prev Med. 2020;59(2):e69-e78. PMID: 32690203. doi: 10.1016/j.amepre.2020.03.008.

57. Champion VL, Rawl SM, Bourff SA, Champion KM, Smith LG, Buchanan AH, et al. Randomized trial of DVD, telephone, and usual care for increasing mammography adherence. Journal of health psychology. 2016;21(6):916-926. doi: 10.1177/1359105314542817.

58. Fissler T, Bientzle M, Cress U, Kimmerle J. The Impact of Advice Seekers' Need Salience and Doctors' Communication Style on Attitude and Decision Making: A Web-Based Mammography Consultation Role Play. JMIR cancer. 2015;1(2):e10. doi: 10.2196/cancer.4279.

59. Henry SL, Shen E, Ahuja A, Gould MK, Kanter MH. The Online Personal Action Plan: A Tool to Transform Patient-Enabled Preventive and Chronic Care. Am J Prev Med. 2016;51(1):71-77. PMID: 26826751. doi: 10.1016/j.amepre.2015.11.014.

60. Klippert H, Schaper A. Using Facebook to communicate mammography messages to rural audiences. Public health nursing (Boston, Mass). 2018:1-8. doi: 10.1111/phn.12556.

61. Krist AH, Woolf SH, Rothemich SF, Johnson RE, Peele JE, Cunningham TD, et al. Interactive preventive health record to enhance delivery of recommended care: a randomized trial. Annals of family medicine. 2012;10(4):312-319. doi: 10.1370/afm.1383.

62. Pereira AAC, Destro JR, Picinin Bernuci M, Garcia LF, Rodrigues Lucena TF. Effects of a WhatsApp-Delivered Education Intervention to Enhance Breast Cancer Knowledge in Women: Mixed-Methods Study. JMIR mHealth and uHealth. 2020;8(7):e17430-e17430. PMID: 32706726. doi: 10.2196/17430.

63. Schapira MM, Hubbard RA, Seitz HH, Conant EF, Schnall M, Cappella JN, et al. The Impact of a Risk-Based Breast Cancer Screening Decision Aid on Initiation of Mammography Among Younger Women: Report of a Randomized Trial. MDM policy & practice. 2019;4(1):2381468318812889. PMID: 30729166. doi: 10.1177/2381468318812889.

64. Walsh J, Potter M, Salazar R, Ozer E, Gildengorin G, Dass N, et al. PreView: a Randomized Trial of a Multi-site Intervention in Diverse Primary Care to Increase Rates of Age-Appropriate Cancer Screening. Journal of General Internal Medicine. 2020;35(2):449-456. doi: 10.1007/s11606-019-05438-0.

65. Bowen DJ, Robbins R, Bush N, Meischke H, Ludwig A, Wooldridge J. Effects of a Web-based intervention on women's breast health behaviors. Translational behavioral medicine. 2011;1(1):155-164. doi: 10.1007/s13142-011-0028-0.
66. Champion VL, Monahan PO, Stump TE, Biederman EB, Vachon E, Katz ML, et al. The Effect of Two Interventions to Increase Breast Cancer Screening in Rural Women. Cancers (Basel). 2022;14(18). PMID: 36139515. doi: 10.3390/cancers14184354.
67. Krist AH, Woolf SH, Hochheimer C, Sabo RT, Kashiri P, Jones RM, et al. Harnessing Information Technology to Inform Patients Facing Routine Decisions: Cancer Screening as a Test Case. Annals of family medicine. 2017;15(3):217-224. doi: 10.1370/afm.2063.
68. Lin Z, Wang S. A tailored Web-based intervention to promote women's perceptions of and intentions for mammography. Journal of Nursing Research (Taiwan Nurses Association). 2009;17(4):249-260. doi: 10.1097/JNR.0b013e3181c15a38.
69. Mathieu E, Barratt AL, McGeechan K, Davey HM, Howard K, Houssami N. Helping women make choices about mammography screening: an online randomized trial of a decision aid for 40-year-old women. Patient education and counseling. 2010;81(1):63-72. doi: 10.1016/j.pec.2010.01.001.
70. Reder M, Kolip P. Does a decision aid improve informed choice in mammography screening? Results from a randomised controlled trial. PloS one. 2017;12(12):e0189148. doi: 10.1371/journal.pone.0189148.
71. Seitz HH, Gibson L, Skubisz C, Forquer H, Mello S, Schapira MM, et al. Effects of a risk-based online mammography intervention on accuracy of perceived risk and mammography intentions. Patient education and counseling. 2016;99(10):1647-1656. doi: 10.1016/j.pec.2016.05.005.
72. Eden KB, Scariati P, Klein K, Watson L, Remiker M, Hribar M, et al. Mammography Decision Aid Reduces Decisional Conflict for Women in Their Forties Considering Screening. Journal of women's health (Larchmt). 2015;24(12):1013-1020. doi: 10.1089/jwh.2015.5256.
73. Eden KB, Ivlev I, Bensching KL, Franta G, Hersh AR, Case J, et al. Use of an Online Breast Cancer Risk Assessment and Patient Decision Aid in Primary Care Practices. J Womens Health (Larchmt). 2020;29(6):763-769. PMID: 32159424. doi: 10.1089/jwh.2019.8143.
74. Scariati P, Nelson L, Watson L, Bedrick S, Eden KB. Impact of a decision aid on reducing uncertainty: Pilot study of women in their 40s and screening mammography Clinical decision-making, knowledge support systems, and theory. BMC Medical Informatics and Decision Making. 2015;15(1). doi: 10.1186/s12911-015-0210-2.
75. Elkin EB, Pocus VH, Mushlin AI, Cigler T, Atoria CL, Polaneczky MM. Facilitating informed decisions about breast cancer screening: development and evaluation of a web-based decision aid for women in their 40s. BMC medical informatics and decision making. 2017;17(1):29. doi: 10.1186/s12911-017-0423-7.
76. Lin ZC, Effken JA. Effects of a tailored web-based educational intervention on women's perceptions of and intentions to obtain mammography. Journal of clinical nursing. 2010;19(9-10):1261-1269. doi: 10.1111/j.1365-2702.2009.03180.x.
77. Arora M, Gerbert B, Potter MB, Gildengorin G, Walsh JM. PRE-VIEW: Development and Pilot Testing of An Interactive Video Doctor Plus Provider Alert to Increase Cancer Screening. ISRN preventive medicine. 2013;2013. doi: 10.5402/2013/935487.
78. Seitz HH, Schapira MM, Gibson LA, Skubisz C, Mello S, Armstrong K, et al. Explaining the effects of a decision intervention on mammography intentions: The roles of worry, fear and perceived susceptibility to breast cancer. Psychology & health. 2018;33(5):682-700. doi: 10.1080/08870446.2017.1387261.
79. Gathirua-Mwangi WG, Monahan PO, Stump T, Rawl SM, Skinner CS, Champion VL. Mammography Adherence in African-American Women: Results of a Randomized Controlled Trial. Annals of behavioral medicine : a publication of the Society of Behavioral Medicine. 2016;50(1):70-78. doi: 10.1007/s12160-015-9733-0.

80. Lin ZC, Effken JA, Li YJ, Kuo CH. Designing a tailored Web-based educational mammography program. Computers, informatics, nursing : CIN. 2011;29(1):16-23. doi: 10.1097/NCN.0b013e3181f9db1c.
81. Lee HY, Lee MH, Gao Z, Sadak K. Development and Evaluation of Culturally and Linguistically Tailored Mobile App to Promote Breast Cancer Screening. Journal of clinical medicine. 2018;7(8). doi: 10.3390/jcm7080181.
82. Klein KA, Watson L, Ash JS, Eden KB. Evaluation of risk communication in a mammography patient decision aid. Patient Educ Couns. 2016;99(7):1240-1248. PMID: WOS:000377934200021. doi: 10.1016/j.pec.2016.02.013.
83. Skinner CS, Buchanan A, Champion V, Monahan P, Rawl S, Springston J, et al. Process outcomes from a randomized controlled trial comparing tailored mammography interventions delivered via telephone vs. DVD. Patient education and counseling. 2011;85(2):308-312. doi: 10.1016/j.pec.2010.10.024.
84. Reder M, Soellner R, Kolip P. Do Women With High eHealth Literacy Profit More From a Decision Aid on Mammography Screening? Testing the Moderation Effect of the eHEALS in a Randomized Controlled Trial. Frontiers in public health. 2019;7:46. doi: 10.3389/fpubh.2019.00046.

111. Reeves B.C., Deeks J.J., Higgins J.P.T., Shea B., Tugwell P., G.A. W. Chapter 24: Including non-randomized studies on intervention effects. In: Higgins JPT, Thomas J, Chandler J, Cumpston M, Li T, Page MJ, Welch VA (editors). Cochrane Handbook for Systematic Reviews of Interventions version 6.3 (updated February 2022). Cochrane, 2022. Available from www.training.cochrane.org/handbook.
. 2022.
112. Lefebvre C GJ, Briscoe S, Featherstone R, Littlewood A, Marshall C, Metzendorf M-I, Noel-Storr A, Paynter R, Rader T, Thomas J, Wieland LS. Chapter 4: Searching for and selecting studies. In: Higgins JPT TJ, Chandler J, Cumpston M, Li T, Page MJ, Welch VA editors. Cochrane Handbook for Systematic Reviews of Interventions version 63 (updated February 2022) Cochrane Available from wwwtrainingcochraneorg/handbook. 2022. ISBN.
113. Craig P, Dieppe P, Macintyre S, Michie S, Nazareth I, Petticrew M. Developing and evaluating complex interventions: the new Medical Research Council guidance. BMJ. 2008;337:a1655. doi: 10.1136/bmj.a1655.
114. Champion VL, Christy SM, Rakowski W, Gathirua-Mwangi WG, Tarver WL, Carter-Harris L, et al. A Randomized Trial to Compare a Tailored Web-Based Intervention and Tailored Phone Counseling to Usual Care for Increasing Colorectal Cancer Screening. Cancer Epidemiol Biomarkers Prev. 2018;27(12):1433-1441. PMID: 30181203. doi: 10.1158/1055-9965.Epi-18-0180.
115. Biederman E, Baltic R, Katz ML, Rawl S, Vachon E, Monahan PO, et al. Increasing breast, cervical, and colorectal cancer screening among rural women: Baseline characteristics of a randomized control trial. Contemp Clin Trials. 2022;123:106986. PMID: 36328234. doi: 10.1016/j.cct.2022.106986.
